# Nuclear Architecture Organized by Rif1 Underpins the Replication-Timing Program

**DOI:** 10.1016/j.molcel.2015.12.001

**Published:** 2016-01-21

**Authors:** Rossana Foti, Stefano Gnan, Daniela Cornacchia, Vishnu Dileep, Aydan Bulut-Karslioglu, Sarah Diehl, Andreas Buness, Felix A. Klein, Wolfgang Huber, Ewan Johnstone, Remco Loos, Paul Bertone, David M. Gilbert, Thomas Manke, Thomas Jenuwein, Sara C.B. Buonomo

**Affiliations:** 1 Mouse Biology Unit, EMBL Monterotondo, Via Ramarini 32, 00015 Monterotondo, Italy; 2 Department of Biological Science, Florida State University, Tallahassee, FL 32306, USA; 3 Max Planck Institute of Immunbiology and Epigenetics, Stubeweg 51, 79108 Freiburg, Germany; 4 European Molecular Biology Laboratory, European Bioinformatics Institute, Wellcome Trust Genome Campus, Cambridge CB10 1SD, UK; 5 Genome Biology and Developmental Biology Units, European Molecular Biology Laboratory, Meyerhofstrasse 1, 69117 Heidelberg, Germany; 6 Wellcome Trust–Medical Research Council Stem Cell Institute, University of Cambridge, Tennis Court Road, Cambridge CB2 1QR, UK; 7 Genome Biology Unit, European Molecular Biology Laboratory, Meyerhofstrasse 1, 69117 Heidelberg, Germany

## Abstract

DNA replication is temporally and spatially organized in all eukaryotes, yet the molecular control and biological function of the replication-timing program are unclear. Rif1 is required for normal genome-wide regulation of replication timing, but its molecular function is poorly understood. Here we show that in mouse embryonic stem cells, Rif1 coats late-replicating domains and, with Lamin B1, identifies most of the late-replicating genome. Rif1 is an essential determinant of replication timing of non-Lamin B1-bound late domains. We further demonstrate that Rif1 defines and restricts the interactions between replication-timing domains during the G1 phase, thereby revealing a function of Rif1 as organizer of nuclear architecture. Rif1 loss affects both number and replication-timing specificity of the interactions between replication-timing domains. In addition, during the S phase, Rif1 ensures that replication of interacting domains is temporally coordinated. In summary, our study identifies Rif1 as the molecular link between nuclear architecture and replication-timing establishment in mammals.

## Highlights

• Most late-replicating regions are marked by Rif1 (RADs)
• Late replication in RADs is differentially regulated where Lamin B1 is stably bound
• Rif1 constrains inter-domain contacts within the same replication timing in G1
• Rif1 coordinates the replication timing of interacting domains

## Graphical Abstract

**Figure:**
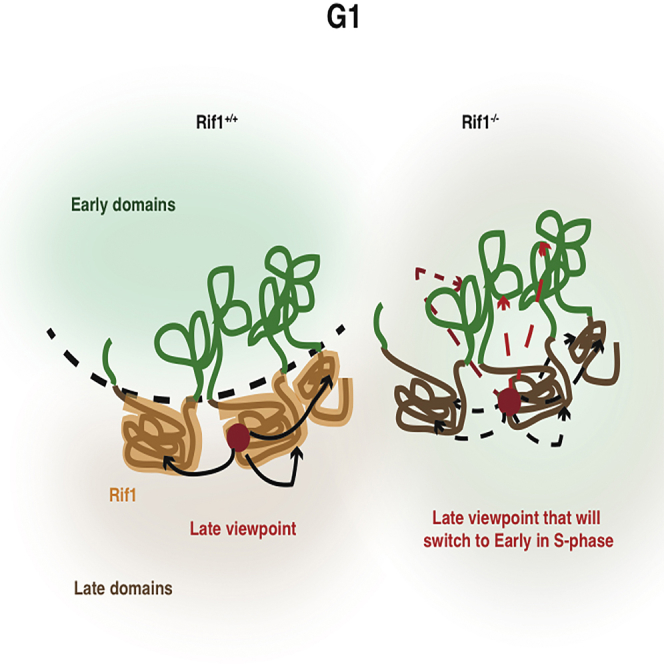

## Teaser

At replication-timing establishment, chromatin domains are forced to interact only with regions sharing the same replication timing. Foti et al. demonstrate that Rif1 is responsible for this constraint because Rif1 deletion leads to loss of spatial limitations followed by replication-timing program disruption. Therefore, Rif1 links nuclear architecture and replication-timing establishment.

## Introduction

The eukaryotic genome is organized into domains whose replication follows a cell-type distinctive temporal order that is defined when the associated replication origins are activated during the S phase (reviewed in Rhind and Gilbert, 2013). In yeast, several origin-binding DNA replication factors are available in limiting amounts. Their interaction either promotes or antagonizes the activation of the loaded helicases at each origin and determines the probability, and thus the order, of firing (Mantiero et al., 2011, Patel et al., 2006, Tanaka et al., 2011, Wu and Nurse, 2009). These findings demonstrate that the execution of the DNA replication-timing (RT) program is controlled at the level of individual origins during the S phase. In contrast, the establishment of the RT program is arranged in mammalian cells in the early G1 phase before the specification of the origins, during the timing decision point (TDP) (Dimitrova and Gilbert, 1999). The TDP coincides with the completion of three-dimensional (3D) chromatin re-organization, after mitosis, suggesting a role for higher-order chromatin organization in defining the temporal DNA replication program (Dileep et al., 2015). Genome-wide analysis of DNA replication domain distribution highlights a striking coincidence with the 3D organization of the chromatin domains (Pope et al., 2014, Ryba et al., 2010, Yaffe et al., 2010). For instance, replication domains precisely overlap with topologically associating domains (TADs), chromatin units defined by high a frequency of interactions, which provides a method of replication domain identification independent of RT (Pope et al., 2014). In addition, changes in RT generally coincide with spatial re-localization of genomic loci relative to the nuclear periphery (Hiratani et al., 2008) and re-organization of chromatin contacts with neighboring loci, allowing maintenance of preferential interactions between domains displaying the same RT (Takebayashi et al., 2012). The establishment of the RT program is therefore independent of the individual origins and may be linked to the spatial organization of the chromatin in the nucleus.

Little is known about the molecular components involved in the establishment of the RT program. In budding yeast, Fhk1/2 influence genome-wide RT by controlling replication origin clustering (Knott et al., 2012). Taz1 in fission yeast counteracts the activation of about half of the late chromosomal origins (Tazumi et al., 2012), while in human cells, polymerase Θ is involved in RT by a yet-unknown mechanism (Fernandez-Vidal et al., 2014). We and others have recently shown that Rif1 is a genome-wide regulator of RT across evolution (Cornacchia et al., 2012, Davé et al., 2014, Hayano et al., 2012, Hiraga et al., 2014, Lian et al., 2011, Mattarocci et al., 2014, Peace et al., 2014, Yamazaki et al., 2012). Rif1 was originally discovered in budding yeast as a negative regulator of telomere length (Hardy et al., 1992), although this role is not conserved in mammals (Buonomo et al., 2009). The telomere-length and RT regulatory functions of *S. cerevisiae* Rif1 are likely connected. Suddenly shortening the telomere induces switching of their late RT to the early S phase (Bianchi and Shore, 2007). Recently, Rif1 was found to cooperate with the protein phosphatase 1 (PP1) to control RT in budding and fission yeast by counteracting origin activation by Dbf4-dependent kinase (Davé et al., 2014, Hiraga et al., 2014, Mattarocci et al., 2014). However, the exact mechanism of this function is still unclear, because Rif1 could not be detected at the origins of replication.

Because RT can be envisaged as a two-stage program, the genome-wide alterations that we observed following Rif1 knockout in fibroblasts (Cornacchia et al., 2012) could reflect defective establishment, an execution step, or both. In this study, we set out to elucidate at what stage and how Rif1 controls the RT program. Because the establishment takes place over large chromosomal regions while the execution occurs at the level of individual origins, analyzing Rif1 genome-wide distribution could provide an important indication of the stage at which it performs its function. We show that Rif1 coats the late-replicating genome, forming large Rif1-associated domains (RADs) with a substantial degree of overlap with Lamin B1-associated domains (LADs). Much evidence associates Rif1 and the lamina (this work; Cornacchia et al., 2012, Yamazaki et al., 2013), a structure with a well-documented role in the organization of chromatin architecture (reviewed in Shimi et al., 2010). We hypothesized that Rif1 could be an organizer of nuclear architecture, linking the establishment of RT with chromatin organization within the nuclear volume. In this study, we provide evidence that Rif1 coordinates inter-domain interactions before S phase and that its loss results first in disorganization of inter-domain chromatin contacts, followed by loss of a stable and reproducible RT program.

## Results

### Rif1 Controls RT across Different Cell Types

**Figure 1:**
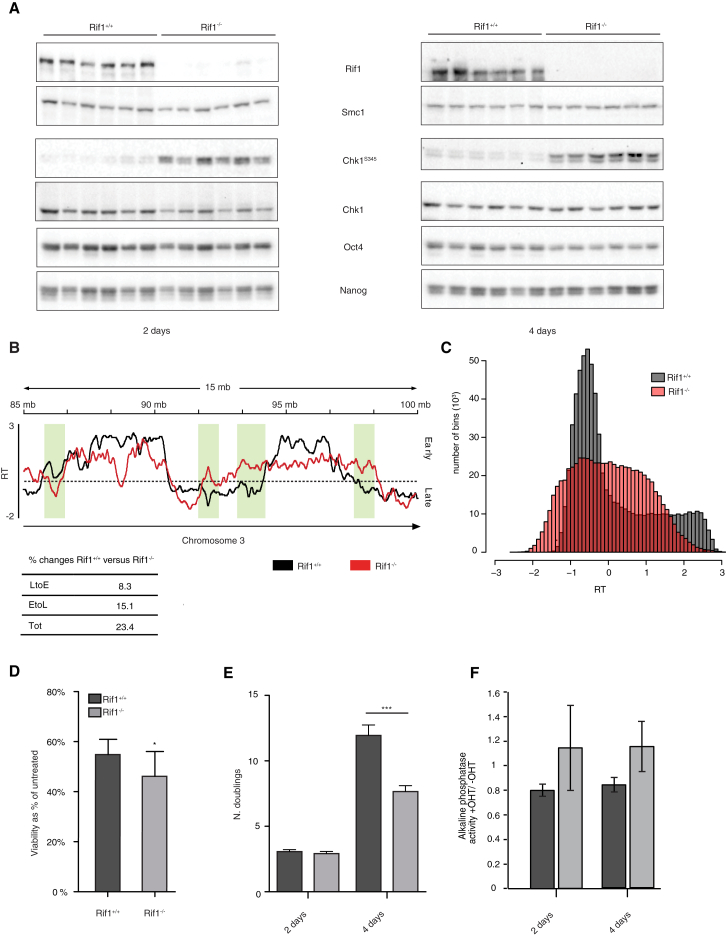
Cell Cycle Responses to Rif1 Deficiency in ESCs (A) Western blot analysis of the Rif1 deletion time course in six independent Rif1^−/−^ and Rif1^+/+^ cells lines. Left and right panels show 2 and 4 days, respectively, after Cre induction. Smc1 is the loading control. (B) Loess smoothed representative RT profiles averaged from two Rif1^+/+^ and four Rif1^−/−^ ESC lines. RT = log_2_(early/late). Regions showing RT switches are shadowed in green. The table summarizes the percentage of RT changes. (C) Using tiles of 60 bp, the genome-wide distribution of the RT scores is shown for averages of two Rif1^+/+^ and four Rif1^−/−^ lines in ESCs. (D) The 3-(4,5-dimethylthiazol-2-yl)-2,5-diphenyltetrazolium bromide (viability) assay 4 days after Cre induction. Shown are the averages from triplicates of six independent Rif1^+/+^ versus Rif1^−/−^ ESCs from three experiments. Error bars indicate SDs, and p values were calculated by t test. (E) Cell proliferation measured as the averages from triplicates of six independent Rif1^+/+^ versus Rif1^−/−^ ESCs from three experiments (paired t test, ^∗∗∗^p < 0.0001). Error bars indicate SDs. (F) Results 2 and 4 days after Cre-induction cells were assayed for alkaline phosphatase activity. +OHT, Rif1^+/+^ or Rif1^F/F^ ESCs treated with 4-hydroxytamoxifen for the indicated duration; −OHT, untreated cells. The average of two biological replicas assayed in triplicates is shown. The error bars indicate SDs. The t test reveals no significant difference. See also Figure S1.

Mouse embryonic stem cells (ESCs) are an ideal model system for the genome-wide study of RT control because of the high percentage of S-phase cells that permit isolation of sufficient material to study the mechanism of Rif1 function and the possible role in nuclear architecture organization. Cre-mediated deletion of Rif1 in ESCs (Figure 1A) induces genome-wide, bi-directional RT switches (Figure 1B), with loss of the typical early/late bi-modal distribution (Figure 1C). The unimodal distribution of genome-wide RT in Rif1^−/−^ cells, centered at zero, suggests loss of temporal resolution of origin firing or, more extremely, that most genomic positions analyzed have the same probability of replicating in the early or late fraction. These data suggest the loss of a stable and reproducible program. In addition, as in primary mouse embryonic fibroblasts (pMEFs) (Cornacchia et al., 2012), deletion of Rif1 induces high levels of fragmentation of the larger replication domains (Figure S1A).

We have previously shown that Rif1 deletion in pMEFs induces the G1/S checkpoint (Cornacchia et al., 2012) and arrests proliferation (Buonomo et al., 2009) because of checkpoint activation (Figure S1B). In contrast, in ESCs, Rif1 deficiency activates the DNA replication checkpoint response (phosphorylation of Chk1 on Ser345; Figure 1A; Figure S1C), decreasing cell viability (Figure 1D) but not arresting proliferation (Figure 1E; Figure S1D).

Because chronic exposure to DNA damage induces differentiation of ESCs (Lin et al., 2005, Qin et al., 2007), we assayed the pluripotency state of Rif1^+/+^ and Rif1^−/−^ ESCs at various stages after Rif1 deletion. We did not detect significant alterations in the levels of expression of the pluripotency markers Oct4 and Nanog (Figure 1A) or in alkaline phosphatase activity (Figure 1F) within the time frame of our experiment (Figures S1E–S1G). In summary, our data indicate that the core function of Rif1 in RT regulation is conserved across different cell types (i.e., ESCs and MEFs), although the cell-type-specific response to its deletion can vary depending on the checkpoint activated.

### Rif1-Bound Domains Identify the Late-Replicating Genome

**Figure 2:**
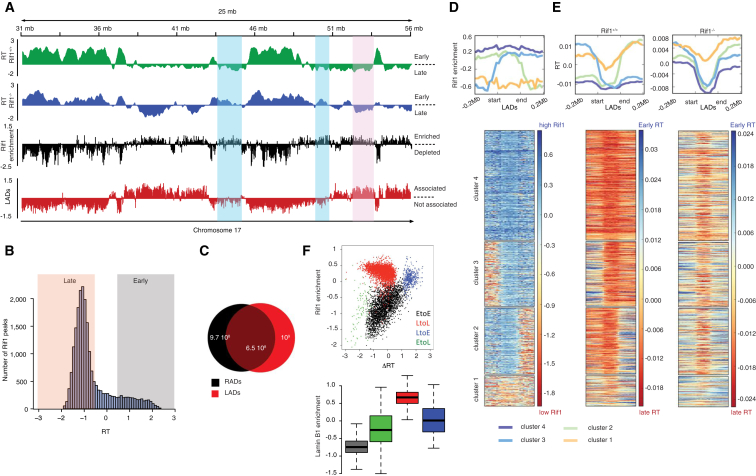
ChIP-Seq Analysis of Genome-wide Rif1 Occupancy in ESCs (A) Representative profile from chromosome 17, comparing RT (RT = log_2_(early/late)) averaged from two Rif1^+/+^ and four Rif1^−/−^ ESCs, with Rif1 distribution from one representative out of three replicas (enrichment = log_2_(ChIP/input)) and LADs. Shadowed in blue are late-replicating RAD-LB^−^, switching to early replication in Rif1^−/−^. In red is highlighted one example of late-replicating RAD-LB^+^, where Rif1 deletion does not affect RT. (B) Distribution of the RT score over the Rif1 binding profile in one representative ESC line out of three. Late-replicating domains (RT ≤ 0.5) are shadowed pink, and early ones are in gray (RT ≥ 0.5). (C) Venn diagram indicating the overlap in base pairs between LADs and RADs, as defined by the EDD algorithm. One representative out of two independent cell lines analyzed is shown. (D) Meta-analysis of Rif1 distribution over LADs. Flanking regions of ±0.2 Mb (non-LADs) were included around the start and end of each LAD. ChIP-seq data were obtained and analyzed from three independent ESC lines. The results presented are from one representative line. The heatmap shows four classes of LADs that were obtained from unsupervised clustering of the Rif1 data and correspond to the different distributions of Rif1 around the LAD boundaries. (E) Replication status of LADs is shown for Rif1^+/+^ and Rif1^−/−^ ESCs. The LADs are ordered in the same way as the cluster solution of Rif1 enrichment in (D). (F) Scatter plot showing Rif1 enrichment relative to the RT changes (ΔRT = Rif1^−/−^ − Rif1^+/+^) and, boxplot showing Lamin B1 association for regions switching (EtoL and LtoE) or not switching (EtoE and LtoL) their RT upon Rif1 deletion. See also Figure S2.

Having validated that the core function of Rif1 is conserved in ESCs, we analyzed its genome-wide occupancy. Rif1’s distribution overlaps extensively with late-replicating regions, while it is generally depleted from early replicating domains (Figures 2A and 2B; Figures S2A and S2B). Because late-replicating regions associate with the nuclear lamina (Hansen et al., 2010, Peric-Hupkes et al., 2010), RADs largely correspond to genomic regions associated with the nuclear lamina (LADs) (Figure 2A) (Peric-Hupkes et al., 2010) and, more specifically, overlap with LADs that are invariant among cell types (cLADs) (Figure S2B) (Meuleman et al., 2013). We confirmed the association of Rif1 and the nuclear lamina by immunofluorescence, where Rif1 is enriched at the nuclear periphery in proximity with Lamin B1 (Figure S2C), and by co-immunoprecipitation of Rif1 and Lamin B1 (Figure S2D). The overlap between RADs and LADs is extensive (Figure 2C). Together, RADs and LADs constitute 73% of all late-replicating regions (Figure S2E), distinguishing two types of late-replicating domains: those that are bound concomitantly by Rif1 and Lamin B1 (RAD-LB^+^) and those that are mostly bound by Rif1 alone (RAD-LB^−^). In contrast, domains marked by Lamin B1 alone tend to have a less defined timing of replication and therefore cannot be strictly classified as late domains (cluster 1 in Figures 2D and 2E). Among the RAD-LB^+^, late RT is independent of Rif1, or is controlled either redundantly or independently of both Rif1 and Lamin B1, because these regions maintain their RT in Rif1 null cells (Figures 2A, 2D, and 2E; late to late [LtoL] in Figure 2F; Figures S2F and S2G). By contrast, RAD-LB^−^ constitute most of the late-replicating regions that switch to early replication in Rif1 null cells (Figures 2A, 2D, and 2E; late to early [LtoE] in Figure 2F; Figures S2F and S2G). Finally we hypothesize that the RT changes that occur within the Rif1-devoided early domains in response to Rif1 deletion (Figure 2A; early to late [EtoL] in Figure 2F) are indirect consequences of the increased competition for limiting S-phase promoting factors due to the earlier replication of RAD-LB^−^ domains, in agreement with what was recently reported for yeast Sir2 (Yoshida et al., 2014). In summary, our data show that Rif1 resides on large domains, remarkably coinciding with late-replicating genomic regions and LADs. This type of distribution suggests a possible role for Rif1 in the 3D organization of the mouse late-replicating genome.

### A Small Fraction of Rif1 Is Focally Enriched at CpG-Rich TSSs

**Figure 3:**
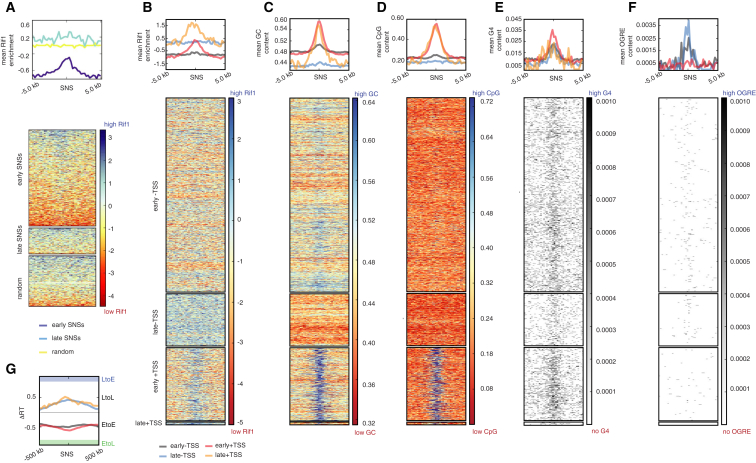
Rif1-Bound Early SNSs Are Not Enriched in EtoL Regions (A) Representative heatmap showing the distribution of the Rif1 signal around SNSs on chromosome 11 for one out of three ESC lines analyzed. For comparison, a random set of loci was chosen from the same region of chromosome 11. (B–F) Analysis of Rif1 enrichment, GC and CpG content, and G4 and OGRE association of early and late SNSs stratified by their overlap with TSSs for one representative out of three ESC lines analyzed. The order of SNSs is identical for all heatmaps. The SNSs were classified as early or late depending on the average score of their replication domains (late: RT < −0.5, early: RT > 0.5, 200 kb bins). (B) Rif1 enrichment (enrichment = log_2_(ChIP/input)) ±5 kb at the center of SNSs. Rif1 presence depends on SNS association with TSSs in early domains and for a very small number of late-replicating, overlapping SNSs and TSSs. (C and D) Analysis of SNSs’ GC and CpG content. TSS-associated SNSs (same cluster displaying Rif1 enrichment) feature high CG (C) and CpG (D) content. Mean CpG content = CpG/(GC/2)^2^. (E) G4 motif instances are plotted as black lines in a discretized matrix at the center of SNSs. The heatmap and average profile reveal that SNSs show a prevalence of G4 motifs in all groups, independent of their replication status or overlap with TSSs. (F) As in (E), but for the less abundant OGRE motif. There is no clear association with SNSs, but there is on average a slight preference for origins without TSSs. (G) Changes of RT (ΔRT = Rif1^−/−^ − Rif1^+/+^) within 500 kb at the center of SNSs. Regions that upon Rif1 deletion change their RT of more than ±1 (LtoE and EtoL; ΔRT > +1 and ΔRT < −1) are considered switching while the others (−1 < ΔRT < +1) are not (EtoE and LtoL). TSS-associated SNSs (same cluster displaying Rif1 enrichment) have the same ΔRT as SNSs that are not associated with TSSs.

**Figure 4:**
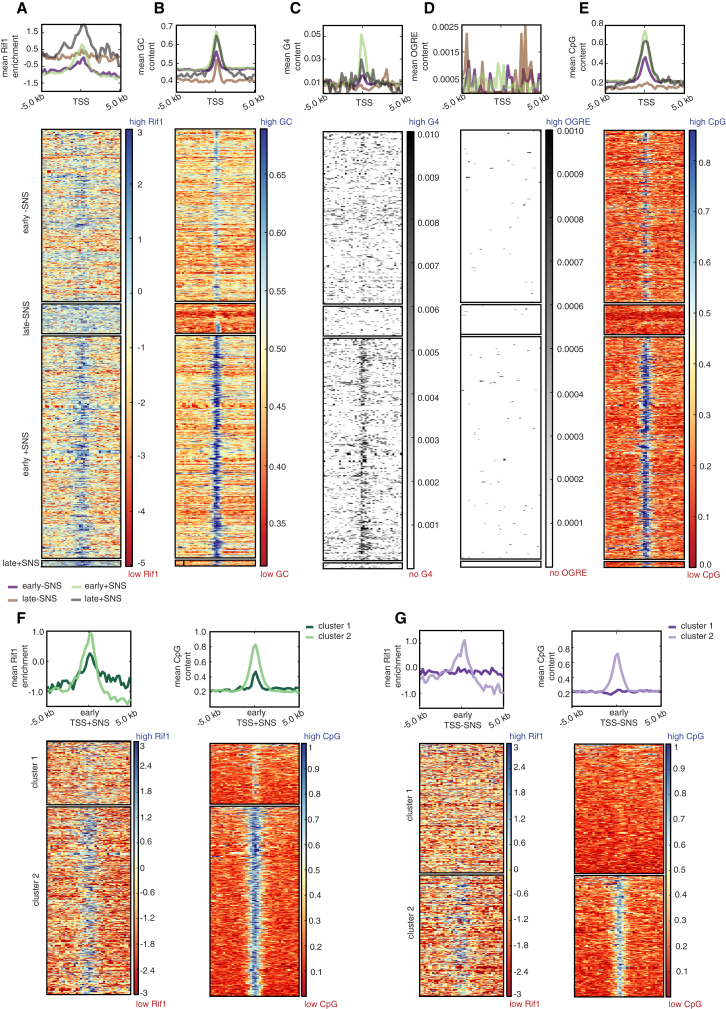
Rif1 Is Associated with CpG-Rich TSSs (A–E) Rif1-bound TSSs in the region of chromosome 11, where SNSs have been mapped, were subdivided based on their overlap with SNSs or lack thereof. Data from one representative ESC line out of three analyzed are shown. (A) The mean Rif1’s enrichment at early TSSs is independent of their association with SNSs. However, SNS presence contributes to better enrichment. (B and E) GC content and CpG ratio surrounding TSSs, respectively. Both features are largely independent of RT of the TSSs and their association with SNSs (apart from the group of late TSSs without SNSs). (C and D) Motif content for G4s and OGREs surrounding TSSs, respectively. A clear enrichment of G4s can be observed around early TSSs with SNSs, while the OGRE motif does not correlate with any of the predefined groups. (F and G) Unsupervised clustering of the two largest TSS groups from the investigated region of chromosome 11, based on the CpG ratio. (F) Early TSSs overlapping with SNSs form two clusters differing in their CpG content. Rif1’s enrichment in each cluster is proportional to the corresponding CpG ratio. (G) Early TSSs not associated to SNSs are clearly divided in CpG-rich and no-CpG clusters. Rif1 is enriched only at CpG-rich TSSs.

While most Rif1 is found in RADs, a fraction of Rif1 is distributed in the form of sharp enrichments in both early and late-replicating regions, as determined by MACS (see Supplemental Information). The sharp peaks formed by mouse Rif1 could represent a fraction of the protein specifically bound to the origins of replication. We therefore compared Rif1 binding to the 2,405 potential DNA replication origins (small nascent strands [SNSs]) mapped on a section of chromosome 11 (Cayrou et al., 2011). Rif1 sharp peaks are associated with 303 (12.6%) SNSs, showing a focal increase with respect to a depleted background in early domains and a broad enrichment in late domains (Figure 3A). This is distinct from fission yeast, where although not bound to, Rif1 tends to be closer to late origins (Hayano et al., 2012). To inspect the association of Rif1 with mouse origins more thoroughly, we integrated Rif1 sharp signals with additional genomic features that have been associated with mammalian origins of replications, such as transcription start sites (TSSs) (Cadoret et al., 2008, Sequeira-Mendes et al., 2009), GC/CpG content, and sequence motifs, such as G quadruplexes (G4s) (Besnard et al., 2012) and the origin G-rich repeated elements (OGREs) (Cayrou et al., 2012). Rif1 is enriched in correspondence with TSS-associated SNSs (Figure 3B), GC rich (Figure 3C), and CpG rich (Figure 3D). The presence of G4s (Figure 3E) or OGREs (Figure 3F) seems instead to be only generically related with presence of SNSs (the former) or TSS-free SNSs (the latter). To investigate whether the preference of Rif1 for TSS-associated, GC/CpG-rich origins is due to a general tendency of Rif1 to bind TSSs, we also performed the inverse analysis. We considered Rif1 distribution around the TSSs on the region of chromosome 11 where SNSs were mapped and stratified them depending on their replication status and association with SNSs. We found that early TSSs in general and late TSSs overlapping with SNSs (a very small number) show an increase of Rif1 (Figure 4A). Rif1’s enrichment is correlated with the GC content (Figure 4B) and, to a lesser extent, with the presence of G4 motifs (Figure 4C), while the presence of OGRE motifs does not show any correspondence (Figure 4D). Most importantly, CpG content seems to be a good predictor of Rif1 enrichment, especially in early replicating regions (Figure 4E). More detailed analysis of the correlation between Rif1 enrichment levels and CpG content revealed that, irrespective of the association with SNSs, CpG-rich TSSs are enriched for Rif1 (Figures 4F and 4G). These are often highly transcribed regions more prone to ChIP artifacts (Teytelman et al., 2013). It would therefore be important to associate Rif1 presence on this small and specific subset of origins with a function. However, Rif1 binding to these SNSs before Cre induction bears no consequences for the RT changes induced by Rif1 deletion in the region. The probability to switch to late replication in Rif1 null cells for SNSs associated or not to TSSs (and Rif1) is indistinguishable (Figure 3G).

In summary, most base pairs covered by Rif1 is in the shape of late domains (Figures 2A and 2B) that are mostly depleted of mapped SNSs. The bulk of mapped origins resides in early domains and is depleted of Rif1, except for a small subset of CpG-rich TSSs-associated SNSs. However, this subgroup does not show any specific response to Rif1 deletion, and Rif1 peaks in early domains do not fit the functional model drawn based on the yeast data. Because our analysis was limited to a section of chromosome 11, we cannot formally exclude that Rif1 could bind to a specific subclass of replication origins. However, overall, our data argue against the idea that Rif1’s control of RT is exerted primarily at the level of individual origins.

### Long-Term Proliferation in Absence of Rif1 Leads to Gene Expression Changes

Rif1 association to a large set of TSSs (Figure S3A; Table S1) could alternatively hint at a function during gene expression regulation. We have therefore analyzed the effect of Rif1 loss on an ESC’s gene expression profile, revealing a progressive mild deregulation (Figures S3B and S3C). However, our analysis could not identify any specific link between Rif1 distribution and deregulated genes or their function (Figures S3D and S3E). Because Rif1 deletion in pMEFs does not affect gene expression (Cornacchia et al., 2012), these data suggest that Rif1 is not directly essential for the control of gene expression.

The explanation for such a mild and generic effect on gene expression (and possibly on RT) could be that Rif1 deficiency could induce epigenetic alterations that, in turn, would independently affect both processes. We have therefore analyzed the genome-wide profile of some histone modifications that have been linked to gene expression regulation (H3K4me3, H3K27me3, and H3K9me3), to heterochromatin assembly (H3K9me3 and H4K20me3), or potentially to origins activity (H3K4me3, especially for early origins; H3K9me3 for late origins [reviewed in Rivera et al., 2014]; and H4K20me3 [Beck et al., 2012]) 2 days after Rif1 deletion. As in pMEFs (Cornacchia et al., 2012), we found no effect of Rif1 deficiency on total levels of modified histones in ESCs (data not shown). In addition, their genome-wide distribution (Figures S4A–S4D) or amount localized to specific regions like TSSs (Figures S4E–S4G), SNSs (Figure S5A), or major satellites (Figures S5B and S5C) is unchanged. In summary, we have found no evidence that the effects of Rif1 deficiency could be immediately attributable to an impact on the epigenetic landscape of ESCs.

The effect of Rif1 deficiency on gene expression is apparent only in cell types that, like HeLa (Yamazaki et al., 2012) or ESCs (this work), do not respond to its deletion by p21 upregulation or cell growth arrest. Accordingly, large T antigen-mediated inhibition of the p21-mediated growth arrest in Rif1-deficient MEFs also mildly alters the transcriptome composition (Figure S5D), suggesting that changes in gene expression are secondary to proliferation. In summary, although the correlation between the presence of the few detectable Rif1 sharp peaks and that of several TSSs raises intriguing questions about the relationship between the regulation of DNA replication and transcription, we could not identify at this stage any general functional requirement for Rif1 in the transcriptional control of Rif1-bound TSSs.

### Rif1 Organizes Inter-RT Domain Contacts

**Figure 5:**
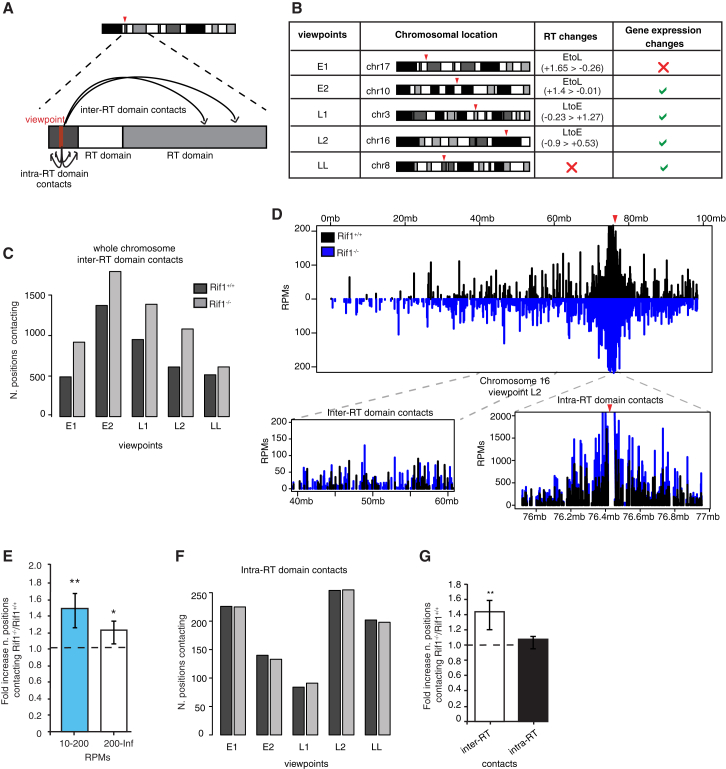
Rif1 Deficiency Affects Inter-RT Domain Interactions in ESCs (A) Schematic representation of the chromatin contacts, highlighting the distinction between inter- and intra-RT domain interactions. Contacts are positions consistently identified by the r3Cseq software package analysis of 4C-seq data in the two replicates for each Rif1^+/+^ and Rif1^−/−^ ESC line. (B) Chromosomal location of each viewpoint and associated properties: RT, region associated with RT switches; gene expression changes, region within 1 Mb of a gene whose expression is affected by Rif1 deletion. (C) Plots showing the total number of same-chromosome contacts per viewpoint. (D) Contacts for the viewpoint L2 (red arrowhead). The whole of chromosome 16 is shown, with the insets displaying zoom-in views of the RT domain around the viewpoint (intra-RT domain interactions) and of a more distal region (inter-RT domain interactions). (E) Ratio (fold increase) between the total of positions, with the number of RPMs indicated on the x axis in Rif1^−/−^ versus Rif1^+/+^ (dashed line), averaged over all viewpoints. Positions are grouped by the supporting number of RPMs as indicated on the x axis. The increase of the number of interactions in both the mid- to low-RPM range (10–200) and the high range (200–information [Inf]) in Rif1^−/−^ is significant, as determined by paired t test (^∗∗^p = 0.006, ^∗^p = 0.03). The error bars indicate SDs. (F) Plots showing the total number of interactions per viewpoint inside the corresponding RT domain. (G) Ratio (fold increase) between the number of interactions averaged over all viewpoints, as shown in (C) and (F), in Rif1^−/−^ over Rif1^+/+^ ESCs (dashed line), taking into consideration the whole genome or only the interactions taking place within the RT domain (paired t test, ^∗∗^p = 0.006). Error bars indicate SDs. See also Figures S5 and S6.

To test whether Rif1 could be an organizer of nuclear 3D architecture, we compared nuclear organization of replication domain interactions in Rif1^+/+^ and Rif1^−/−^ ESCs by circularized chromosome conformation capture sequencing (4C-seq) (Figure 5A). We chose five viewpoints and probed their genome-wide contacts. Three of them are located in regions displaying both RT and transcriptional changes upon Rif1 deletion, while the remaining two represent loci that show either only RT or only gene expression changes (Figure 5B). In each case, Rif1 deficiency significantly increased the total number of positions found in spatial proximity to the viewpoint (contacts) (Figures 5C and 5D). We reasoned that a loss of structured chromatin contacts could result in an increase of random or quasi-random lower-frequency interactions. Compared to the controls, in Rif1 null ESCs, the viewpoints established additional contacts both at high-frequency (200–information) and at low- to mid-frequency (10–200) RPMs (reads per million), where RPM expresses the calibrated number of reads per position and is therefore roughly proportional to the frequency of identification of each contact within the library (Figure 5E; Figure S5E). However, in agreement with our prediction, the gains were particularly significant within the low- to mid-frequency range.

To explore whether Rif1’s role in chromatin organization could be involved in defining the boundaries of a single RT domain, we analyzed whether the 3D organization of the single RT domain is affected by Rif1 deletion. We identified the boundaries of the RT domains encompassing the viewpoints by comparison of RT profiles derived from different cell types (see Supplemental Experimental Procedures). Unlike the number of positions interacting with the viewpoint over the length of the chromosome, contacts within the replication domain are not affected (Figures 5D and 5F). The different outcome of Rif1 deficiency on the total versus the intra-domain contacts (Figures 5D and 5G) suggests that the definition of the replication domain remains unaffected. Instead, the organization of inter-domain interactions has been lost. To independently validate the 4C data and appreciate their qualitative behavior, we performed three further analyses. First, we validated several contact frequency increases by 3C-qPCR (Figure S5F). Second, we used 3D fluorescence in situ hybridization (FISH) to validate the increased proximity of one of the contacts (Figure S5G). Third, an independent analysis of the 4C-seq data with FourCSeq (Figure S6) (see Supplemental Experimental Procedures) (Klein et al., 2015), confirmed a consistent difference between the two conditions. Altogether, these results establish Rif1 as a spatial organizer of chromatin.

### Rif1 Controls 3D Chromatin Organization in the G1 Phase

**Figure 6:**
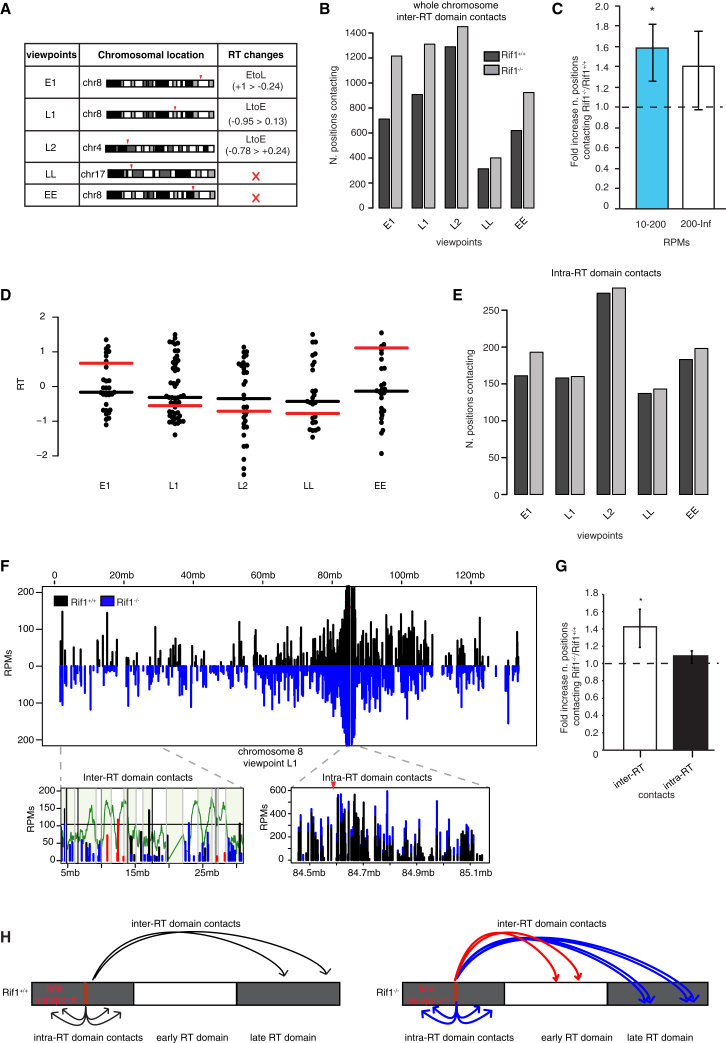
Rif1 Deletion Affects Nuclear Architecture during the G1 Phase Contacts are positions consistently identified by the r3Cseq software package analysis of 4C-seq data in the two replicates for each Rif1^+/+^ and Rif1^−/−^ pMEF line in the first G1 phase after deletion. One representative experiment out of two is shown. (A) Summary of the chromosomal location of each viewpoint and associated features. RT, region associated with RT switches. In pMEFs, there are no gene expression changes induced by Rif1 deletion. (B) Plots showing the total number of same-chromosome interactions per viewpoint. (C) Ratio (fold increase) between the total of positions in Rif1^−/−^ versus Rif1^+/+^ (dashed line). Positions are divided in two classes depending on the number of supporting RPMs. The increase of the number of positions in the low- to mid-RPM range (10–200) in Rif1^−/−^ is significant, as determined by paired t test (^∗^p = 0.02). Error bars indicate the SDs. (D) Distribution of RT (RT = log_2_(early/late)) of the 4C-seq contacts within the TADs that are interacting with the indicated viewpoints, specifically in Rif1^−/−^ pMEFs in the G1 phase. The black line indicates their median RT. The red line is the median RT of the TADs that interact with the viewpoint in both synchronized Rif1^+/+^ and Rif1^−/−^ pMEFs in Figure S7D and is placed as a reference to appreciate the difference. (E) Plots showing the total number of contacts per viewpoint inside the corresponding RT domain. (F) Contacts for the viewpoint L1 (red arrowhead). The whole of chromosome 8 is shown, with the insets displaying zoom-in views of the RT domain around the viewpoint (right) or a more distal region (left). The insets also show the distributions of TADs in the same regions (gray lines and alternate green shadowing). The RT domain (right) is fully enclosed in a single TAD. In the inset showing the distal region (left), the contacts mapping in TADs that selectively interact with the viewpoint in Rif1^−/−^ cells are shown in red. In green, the RT profile of synchronous Rif1^+/+^ pMEFs is shown. (G) Ratio between the number of interactions averaged over all viewpoints, as shown in (B) and (E), in Rif1^−/−^ over Rif1^+/+^ pMEFs (dashed line) calculated for the chromosome hosting the viewpoint (in cis) or only inside the RT domain (paired t test, ^∗^p = 0.01). Error bars indicate SDs. (H) Schematic interpretation of the data in (B), (E), and (F), illustrating the gain of inter-RT domain interactions (arrows) and the loss of RT specificity of some acquired contacts in Rif1^−/−^ pMEFs for a putative viewpoint (red). The interactions established by the viewpoint in Rif1^+/+^ are represented by black arrows; the ones established by Rif1^−/−^ cells that fall into TADs shared with Rif1^+/+^ are blue. The new contacts established by the viewpoint exclusively in Rif1^−/−^ cells and that fall into gained TADs are represented by red arrows. See also Figure S7.

The loosening of spatial control of chromatin interactions observed in Rif1^−/−^ ESCs could be a direct consequence of Rif1 deficiency or a secondary effect of RT changes. To discriminate between these two possibilities, chromatin architecture must be analyzed in the first G1 phase after Rif1 deletion before the first round of replication and the onset of RT deregulation. This experiment is not feasible in ESCs, because they cannot be arrested at any cell cycle stage long enough to obtain sufficient levels of Rif1 deletion, which requires approximately 2 days. We had previously used pMEFs synchronized and deleted in G0 phase to show that failure to re-express Rif1 upon re-entry into G1 phase induces RT deregulation during the first S phase (Figure S7A) (Cornacchia et al., 2012). We therefore employed the same system to try to understand whether the observed altered chromatin organization caused by Rif1 deletion follows or precedes RT deregulation (Figure S7B). Analogous to ESCs, we chose five 4C-seq viewpoints (Figure 6A). Remarkably, we found that Rif1^−/−^ pMEFs, like cycling ESCs, show an increased number of chromosomal positions contacting the viewpoint at a low to medium frequency (10–200 RPMs) and this is already observable during the first G1 phase after deletion (Figures 6B and 6C; Figure S7C).

This synchronization strategy enables us to uncouple the effect of Rif1 deletion on nuclear architecture from its effects on the timing of DNA replication, allowing us to analyze the relation between the changes of chromatin contacts caused in G1 phase by Rif1 deletion and the changes of RT in the following S phase. To this end, we first grouped into TADs the positions interacting with the viewpoint in G1 phase, identified by 4C-seq in synchronized pMEFs. By this classification, we can understand whether the increase of chromatin contacts that we observe in Rif1^−/−^ cells in G1 phase is limited within the same units (TAD) already interacting with the viewpoint in Rif1^+/+^ cells. Alternatively, it could be accompanied by the creation of contacts between the viewpoint and the additional TADs and, if so, we can analyze their RT compared to the viewpoint. Chromatin contacts are normally established among regions (Takebayashi et al., 2012) and more specifically between TADs (Pope et al., 2014) with the same RT. Our analysis reveals that a subset of the specific contacts established in the Rif1^−/−^ cells reflects the creations of novel interactions with additional TADs, while others are common to Rif1^+/+^ and null cells. The RT of the TADs shared between Rif1^+/+^ and Rif1^−/−^ cells is the same as the RT of the viewpoint (Figure 6F, inter-domain RT interactions; Figure S7D, black boxplots), and it shifts toward zero only as Rif1^−/−^ cells undergo S phase (Figure S7D, gray boxplots). This suggests that during the first S phase, the coordination of RT between the TAD containing the viewpoint and its interactors is lost, with some TADs shifting RT like the viewpoint while others do not. However, because the RT of the TADs that specifically interact with the viewpoint only in Rif1^−/−^ cells is already shifted toward zero in the G1 phase (Figure 6D, black bar), the new contacts formed before S phase must be random. These results show that loss of definition of the organization of chromatin contacts induced by Rif1 deficiency precedes the RT changes. More importantly, the newly acquired interactions do not conform to the RT of the viewpoint (Figure 6F, red lines), linking the change of contacts with the following change of RT. Our data support the hypothesis that Rif1-dependent stable and compartmentalized chromatin contacts in G1 phase could instruct a functional RT program in S phase. In addition, the 3D nuclear substructures defined by Rif1 in the G1 phase will undergo a coordinated replication in the following S phase.

Consistent with ESCs, the pMEFs showed no significant change in the number of positions interacting with the viewpoint within the boundaries of the replication domain (Figures 6E and 6F, intra-RT domain interactions), indicating that Rif1 deficiency mostly affects the organization of inter-domain contacts (Figure 6G). In agreement with this conclusion, we observed that RT switches in Rif1 null pMEFs take place in correspondence with the RT domain borders as developmentally defined by the alignment of RT profiles of multiple cell lineages (Figure S7E). This suggests that Rif1 deficiency does not affect the identity of the minimal RT unit.

In summary, by employing this synchronization strategy, we have been able to demonstrate that Rif1 is necessary for the determination of chromatin architecture; i.e., it limits the contacts between RT domains (Figure 6H) in G1 phase, independent of replication. These results suggest the possibility that Rif1’s earliest function is concurrent with the establishment of RT.

## Discussion

Although the temporal organization of the initiation of DNA replication was first reported 57 years ago (Taylor, 1958, Taylor, 1960), the genetic and molecular control underlying this process remains unclear. Consequently, its biological significance remains undetermined. At the genomic level, RT is dictated by a regional compartmentalization into chromatin domains that replicate simultaneously, known as RT domains (Pope et al., 2014). Here we sought to understand Rif1’s molecular function and interrogated its possible involvement in bridging RT and nuclear architecture. Chromatin immunoprecipitation sequencing (ChIP-seq) analysis of Rif1 genome-wide distribution reveals that in ESCs Rif1 displays a domain type of binding (RAD), covering large (∼1 Mb), late-replicating genomic regions, which largely overlap with LADs (RAD-LB^+^). Accordingly, Rif1 both co-localizes and co-immunoprecipitates with Lamin B1. Altogether, these data suggest a possible chromatin-organizing function of Rif1 during RT definition. In agreement with this hypothesis, using 4C-seq analysis, we show here that deletion of Rif1 affects chromatin contacts between different replication domains. This is not an indirect result of cell proliferation in the presence of deregulated RT but rather an immediate consequence of the absence of Rif1. The effect of Rif1 deficiency on nuclear architecture is already evident in synchronized pMEFs in the first G1 phase after deletion, and thus before changes of RT are enacted. Our data indicate that Rif1’s effect on RT control ensues from its function of defining chromatin interactions during the G1 phase. Rif1 couples stable nuclear sub-compartments with specific RT. The first consequence of Rif1 deletion is the weakening of nuclear sub-compartmentalization, followed by a loss of coordinated replication (e.g., fragmentation of larger replication domains) and the acquisition of new and unstable RT, at least for a fraction of the genome. Within the RADs, we could distinguish two sub-types of late-replicating domains, set apart by different levels of Lamin B1 binding. Rif1 is essential for late replication only in RAD-LB^−^. These data indicate that additional levels of control, in the context of the structural subnuclear unit defined by the LADs, render RT in these regions resistant to the changes induced by Rif1 deficiency. What distinguishes EtoE (early-to-early) from EtoL domains remains unclear. However, the reproducibility of the early domains switching to late replication suggests the existence of underlying yet unknown regional features.

Recently, it has been shown that Rif1’s function during RT control in yeast is mediated through its interaction with PP1 (Davé et al., 2014, Hiraga et al., 2014, Mattarocci et al., 2014). Because mammalian Rif1 also harbors two potential PP1-interaction motifs (Sreesankar et al., 2012) and was identified among PP1α interactors (Trinkle-Mulcahy et al., 2006), it will be interesting to determine whether this interaction has a function in chromatin 3D organization and/or determine the timing of origin firing in mammalian cells. If mammalian Rif1 functions mainly as PP1 adaptor, we can envisage two molecular mechanisms by which Rif1 could link 3D nuclear organization to RT control. Rif1 could be a molecular hub that couples nuclear architecture and RT by affecting both processes in parallel, for example, by targeting PP1 to substrates independently involved in these two processes. Alternatively, Rif1 could instruct RT at two levels. First, by organizing the architecture of early and late domains in the G1 phase, Rif1 could set one level of RT control through 3D compartmentalization of origins. Subsequently, it could schedule their firing by PP1-mediated MCM4 de-phosphorylation. Although more work is needed to clarify these molecular aspects, our data support the latter view, because Rif1 deficiency affects the RT specificity of chromatin contacts before S phase. In our view, this is suggestive of a hierarchical, rather than a parallel, independent effect of Rif1 on nuclear architecture and RT. We propose that Rif1 could define in 3D the late domains at the time of RT establishment in G1 phase and then translate this organization into a threshold for origin initiation in S phase by controlling a regional recruitment of high levels of PP1, as the yeast data also suggest, within the non-LAD late regions.

Overall, our data point to Rif1 being the molecular link between chromatin 3D organization and RT determination. This view is backed by the partial information available concerning its structure. It was recently shown that budding yeast Rif1 can tetramerize (Shi et al., 2013). Multimerization and direct DNA binding have also been shown for the mammalian protein (Sukackaite et al., 2014, Xu et al., 2010; S.C.B.B., unpublished data), suggesting that Rif1 could form a nuclear network organizing chromatin loops and their reciprocal positioning in the nuclear volume. Rif1 has also been shown to function during DNA repair (Buonomo et al., 2009, Chapman et al., 2013, Di Virgilio et al., 2013, Feng et al., 2013, Silverman et al., 2004, Wang et al., 2009, Xu et al., 2010, Zimmermann et al., 2013). Our findings could reconcile these apparently diverse functions attributed to Rif1, as controlling and limiting the number and spatial distribution of chromatin interactions could be part of how Rif1 contributes to regulating repair.

### Replication Timing and Transcription: Two Sides of the Same Coin

In the recent years, it has been shown that the organization of DNA RT is a cell-type-specific signature as unique as the gene expression profile and that it undergoes profound remodeling during development. These features parallel the epigenetic and gene expression regulations, but a direct, universal link among these three biological pathways has yet to be found. Although the paradigm of “expressed gene equals early replicating domain” while “silenced gene equals late-replicating domain” is generally true, genome-wide studies have shown that there are many significant exceptions (Hiratani et al., 2010, Rivera-Mulia et al., 2015). Changing the transcriptional status does not always imply a change in RT, and vice versa. Here we show that the establishment of chromatin organization during the G1 phase affects the order of replication of different domains. Changing nuclear architecture induces alterations of RT but in time also translates into changes of the gene expression profile, in agreement with subnuclear positioning having a well-established role in modulating gene expression (Andrulis et al., 1998, Finlan et al., 2008, Mattout et al., 2011, Peric-Hupkes et al., 2010, Reddy et al., 2008, Zullo et al., 2012). Our data also indicate that these changes can take place with the epigenetic landscape hardly being affected, at least in the short term. Nuclear architecture is therefore the common determinant for both gene expression and RT. This finding is an important step toward an understanding of the complex and confusing relationship between the two processes that are linked, though not by causality. Our data imply that RT regulation and nuclear architecture are intricately connected to the extent that it could render difficult to uncouple the question of the biological significance of the RT program from the role of gene expression in establishing cell identity. For future studies addressing this fundamental issue, our work identifies the architectural organization of the Rif1-sensitive fraction of the genome (RAD-LB^−^) as a uniquely dynamic component in which gene expression regulation and timing of replication probably integrate, converging to the determination of cell identity.

## Experimental Procedures

### Rif1 ChIP

Rif1^FH/FH^ and Rif1^+/+^ ESCs have been cross-linked first with 2 mM disuccinimidyl glutarate for 45 min and then in 1% formaldehyde (FA) for 10 min. Immunoprecipitation was performed using the Roche anti-hemagglutinin antibody. See Supplemental Experimental Procedures for full details.

### ChIP-Seq for Histone Modifications

Rif1^−/−^ and Rif1^+/+^ ESCs have been cross-linked in 1% FA for 10 min. Immunoprecipitation was performed using anti-H3K4me3, anti-H3K9me3, anti-H3K27me3, or anti-H4K20me3 antibodies. See Supplemental Experimental Procedures for full details.

### 4C-Seq

ESCs or pMEFs were cross-linked in 2% FA. Primary restriction digest was performed by incubation with HindIII, and secondary with was performed by incubation DpnII. Libraries have been sequenced in 100 bp single-end mode. See Supplemental Experimental Procedures for full details.

## Author Contributions

R.F. performed most of the experiments and the 4C-seq analysis; S.G. performed 3D FISH, ChIP-qPCR, and ChIP-seq; D.C. contributed to ESC derivation and performed ChIP-seq and immunoprecipitation; V.D. performed the RT experiment and alignment of profiles, helped designing the 4C-seq primers, and analyzed Rif1 distribution in relation to RT switches; A.B.-K. taught the way to perform the ChIP-seq; E.J. and R.L. advised on the 4C-seq analysis; A.B., F.A.K., and W.H. performed the FourCSeq analysis; T.M. and S.D. analyzed the ChIP-seq data; D.M.G. critically read the manuscript and contributed with scientific advice; and T.J. and P.B. hosted members of the S.C.B.B. lab during training. S.C.B.B. designed the experiments and wrote the manuscript.

## Accession Numbers

The accession number for the chromosome conformation capture data from Rif1 conditional knockout and Rif1 wild-type mouse ESCs reported in this paper is Array Express: E-MTAB-3500. The accession number for the chromosome conformation capture from synchronized Rif1 pMEFs reported in this paper is Array Express: E-MTAB-3505. The accession number for the gene expression profiles from Rif1 conditional knockout and Rif1 wild-type large T immortalized MEFs reported in this paper is Array Express: E-MTAB-3501. The accession number for the gene expression profiles from Rif1 conditional knockout and Rif1 wild-type mouse ESCs reported in this paper is Array Express: E-MTAB-3503. The accession number for the genome-wide occupancy (ChIP-seq) data from Rif1^FH^ in mouse ESCs reported in this paper is Array Express: E-MTAB-3502. The accession number for the RT profiles from Rif1 conditional knockout and Rif1 wild-type mouse ESCs reported in this paper is Array Express: E-MTAB-3506. The accession number for the genome-wide distribution (ChIP-seq) of histone modifications from Rif1 conditional knockout and Rif1 wild-type mouse ESCs reported in this paper is Array Express: E-MTAB-3743.

## Supplemental Information

Document S1. Supplemental Experimental Procedures, Figures S1–S7, and Tables S1–S4

Document S2. Article plus Supplemental Information
