# Supplementary material for: Nuclear Architecture Organized by Rif1 Underpins the Replication-Timing Program
Source: Mol Cell. 2016 Jan 21;61(2):260–73. doi: 10.1016/j.molcel.2015.12.001 (PMC4724237; doi:10.1016/j.molcel.2015.12.001)
Supplement: Document S1. Supplemental Experimental Procedures, Figures S1–S7, and Tables S1–S4 [file mmc1.pdf]

Molecular Cell

Supplemental Information

Nuclear Architecture Organized by Rif1 Underpins the Replication-Timing Program

Rossana Foti, Stefano Gnan, Daniela Cornacchia, Vishnu Dileep, Aydan Bulut-Karslioglu, Sarah Diehl, Andreas Buness, Felix A. Klein, Wolfgang Huber, Ewan Johnstone, Remco Loos, Paul Bertone, David M. Gilbert, Thomas Manke, Thomas Jenuwein, and Sara C.B. Buonomo

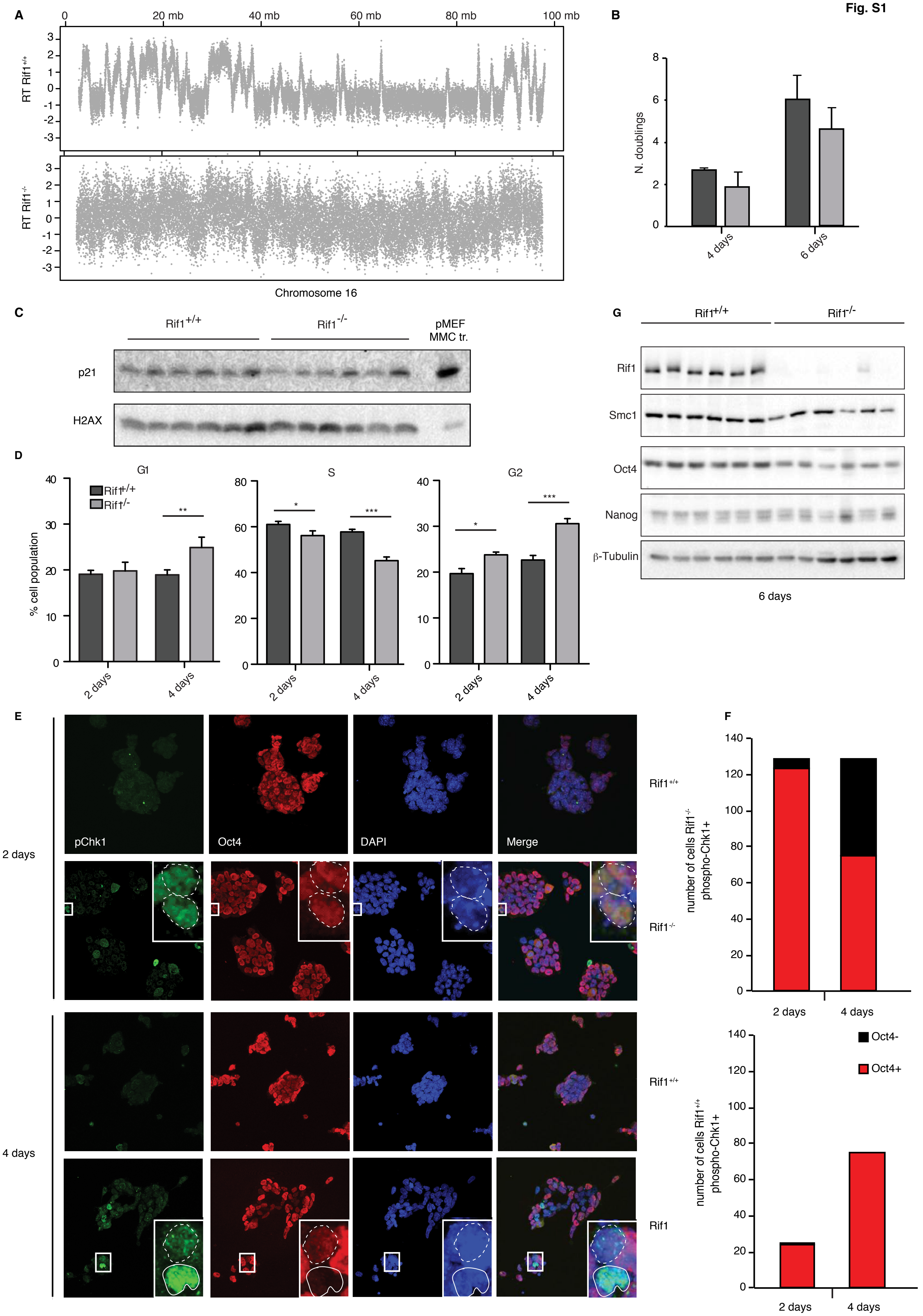

Fig. S1
A
0
20 mb
40 mb
60 mb
80 mb
100 mb
RT Rif1+/+
RT Rif1-/-
Chromosome 16
B
N. doublings
4 days
6 days
C
Rif1+/+
Rif1-/-
pMEF
MMC tr.
p21
H2AX
D
G1
S
G2
Rif1+/+
Rif1-/-
% cell population
2 days
4 days
G
Rif1
Smc1
Oct4
Nanog
β-Tubulin
6 days
E
pChk1
Oct4
DAPI
Merge
2 days
4 days
F
number of cells Rif1-/- phospho-Chk1+
number of cells Rif1+/+ phospho-Chk1+
Oct4-
Oct4+

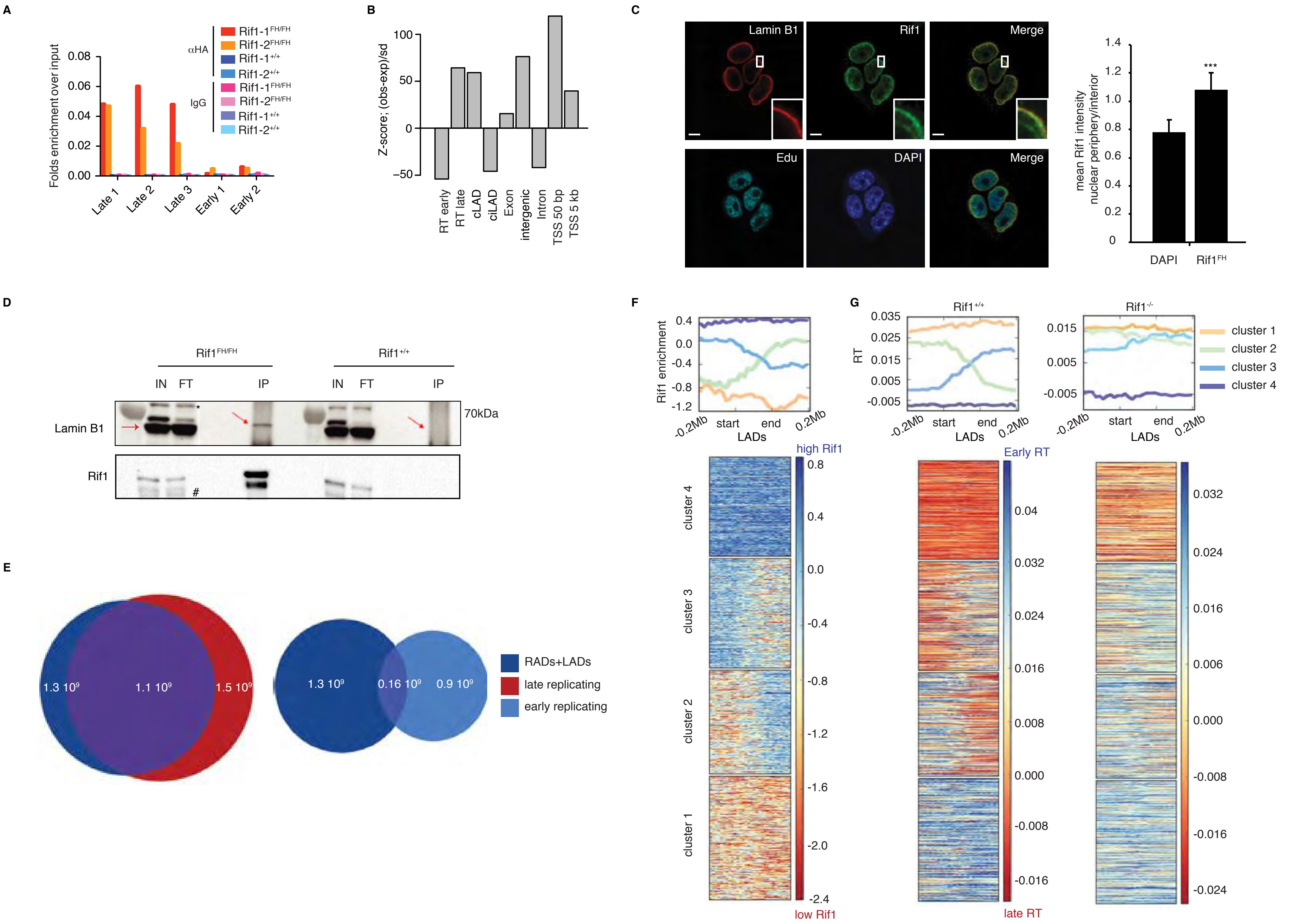
A
Folds enrichment over input
αHA
IgG
Late 1
Late 2
Late 3
Early 1
Early 2
B
Z-score; (obs-exp)/sd
RT early
RT late
cLAD
ciLAD
Exon
intergenic
Intron
TSS 50 bp
TSS 5 kb
C
Lamin B1
Rif1
Merge
Edu
DAPI
mean Rif1 intensity nuclear periphery/interior
DAPI
***
D
IN
FT
IP
Lamin B1
Rif1
70kDa
#
E
1.3 10⁹
1.1 10⁹
1.5 10⁹
0.16 10⁹
0.9 10⁹
RADs+LADs
late replicating
early replicating
F
Rif1 enrichment
start
end
LADs
high Rif1
low Rif1
cluster 1
cluster 2
cluster 3
cluster 4
G
RT
Early RT
late RT

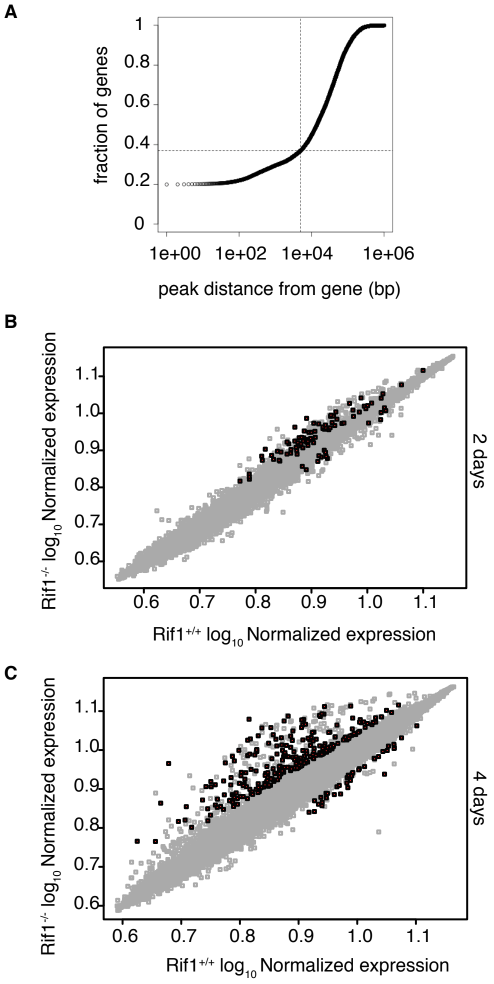

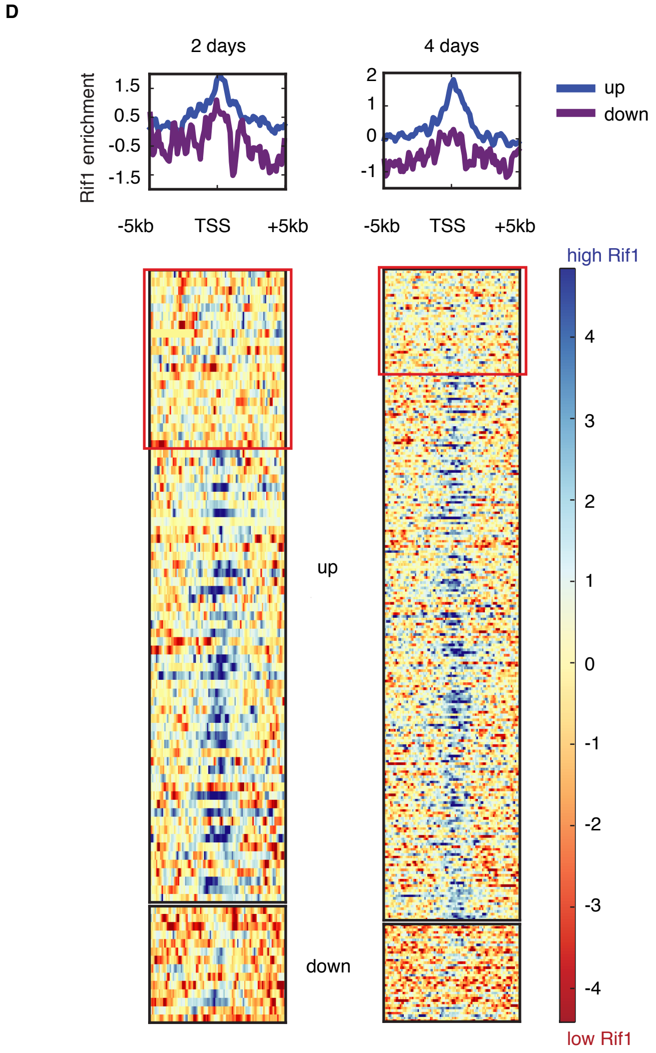

E

Top-scoring GO categories

GO categories	P
Metabolic processes	0.09
mRNA splicing	0.4
RNA processing	0.7
Developmental processes	1.00

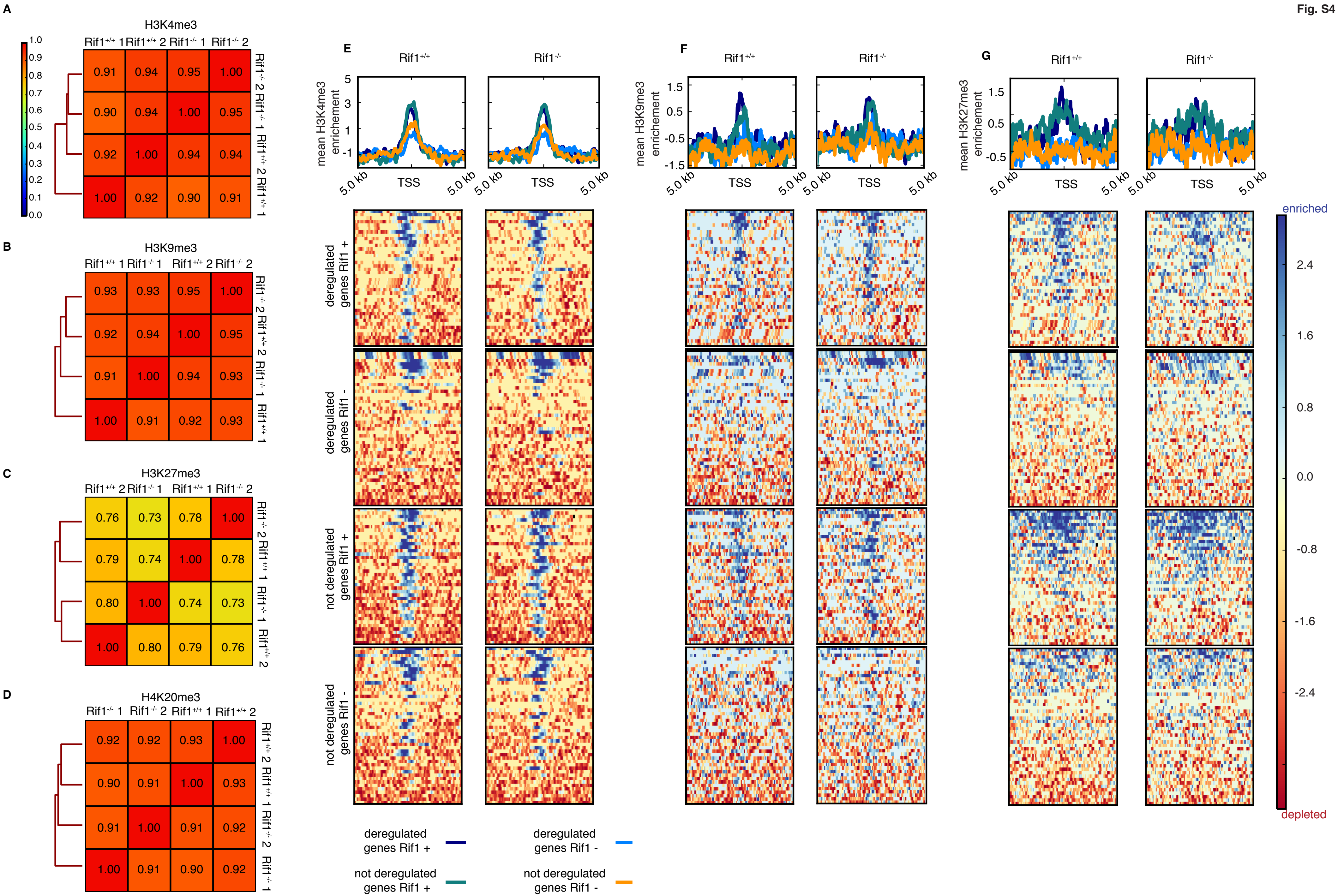

A
H3K4me3
Rif1+/+ 1 Rif1+/+ 2 Rif1-/- 1 Rif1-/- 2
0.91 0.94 0.95 1.00
0.90 0.94 1.00 0.95
0.92 1.00 0.94 0.94
1.00 0.92 0.90 0.91
Rif1-/- 2 Rif1-/- 1 Rif1+/+ 2 Rif1+/+ 1
1.0 0.9 0.8 0.7 0.6 0.5 0.4 0.3 0.2 0.1 0.0
B
H3K9me3
Rif1+/+ 1 Rif1-/- 1 Rif1+/+ 2 Rif1-/- 2
0.93 0.93 0.95 1.00
0.92 0.94 1.00 0.95
0.91 1.00 0.94 0.93
1.00 0.91 0.92 0.93
Rif1-/- 2 Rif1+/+ 2 Rif1-/- 1 Rif1+/+ 1
C
H3K27me3
Rif1+/+ 2 Rif1-/- 1 Rif1+/+ 1 Rif1-/- 2
0.76 0.73 0.78 1.00
0.79 0.74 1.00 0.78
0.80 1.00 0.74 0.73
1.00 0.80 0.79 0.76
Rif1-/- 2 Rif1+/+ 1 Rif1-/- 1 Rif1+/+ 2
D
H4K20me3
Rif1-/- 1 Rif1-/- 2 Rif1+/+ 1 Rif1+/+ 2
0.92 0.92 0.93 1.00
0.90 0.91 1.00 0.93
0.91 1.00 0.91 0.92
1.00 0.91 0.90 0.92
Rif1+/+ 2 Rif1+/+ 1 Rif1-/- 2 Rif1-/- 1
E
Rif1+/+
Rif1-/-
mean H3K4me3 enrichement
5 3 1 -1
5.0 kb TSS 5.0 kb
F
mean H3K9me3 enrichement
1.5 0.5 -0.5 -1.5
G
mean H3K27me3 enrichement
1.5 0.5 -0.5
deregulated genes Rif1 +
deregulated genes Rif1 -
not deregulated genes Rif1 +
not deregulated genes Rif1 -
enriched
2.4 1.6 0.8 0.0 -0.8 -1.6 -2.4
depleted

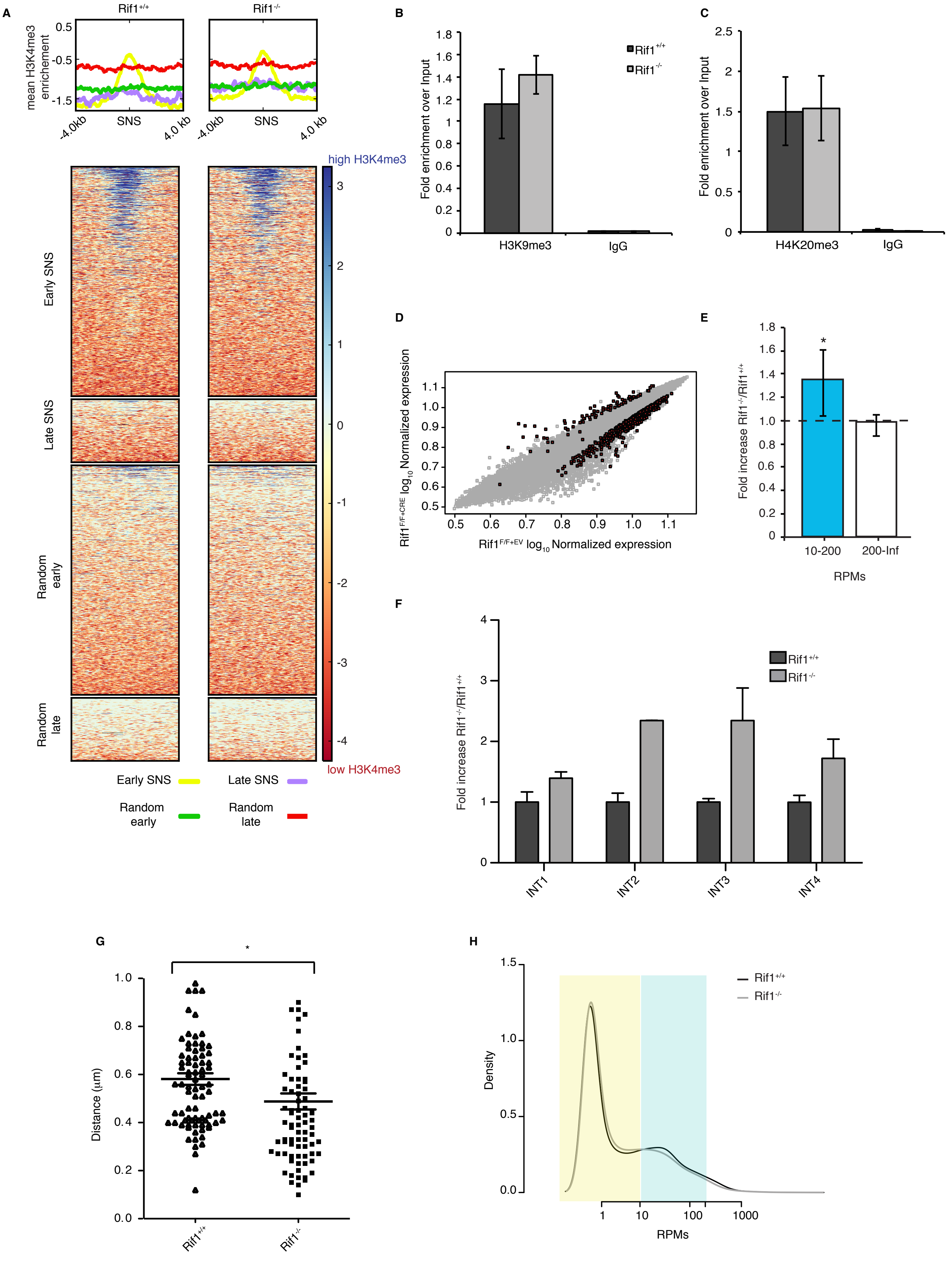
A
Rif1+/+
Rif1-/-
mean H3K4me3 enrichement
0.5
-0.5
-1.5
-4.0kb
SNS
4.0 kb
high H3K4me3
low H3K4me3
Early SNS
Late SNS
Random early
Random late
3
2
1
0
-1
-2
-3
-4
B
Fold enrichment over Input
Rif1+/+
Rif1-/-
H3K9me3
IgG
C
H4K20me3
D
Rif1F/F+CRE log10 Normalized expression
Rif1F/F+EV log10 Normalized expression
E
Fold increase Rif1-/-/Rif1+/+
10-200
200-Inf
RPMs
F
INT1
INT2
INT3
INT4
G
Distance (μm)
H
Density
RPMs
1
10
100
1000

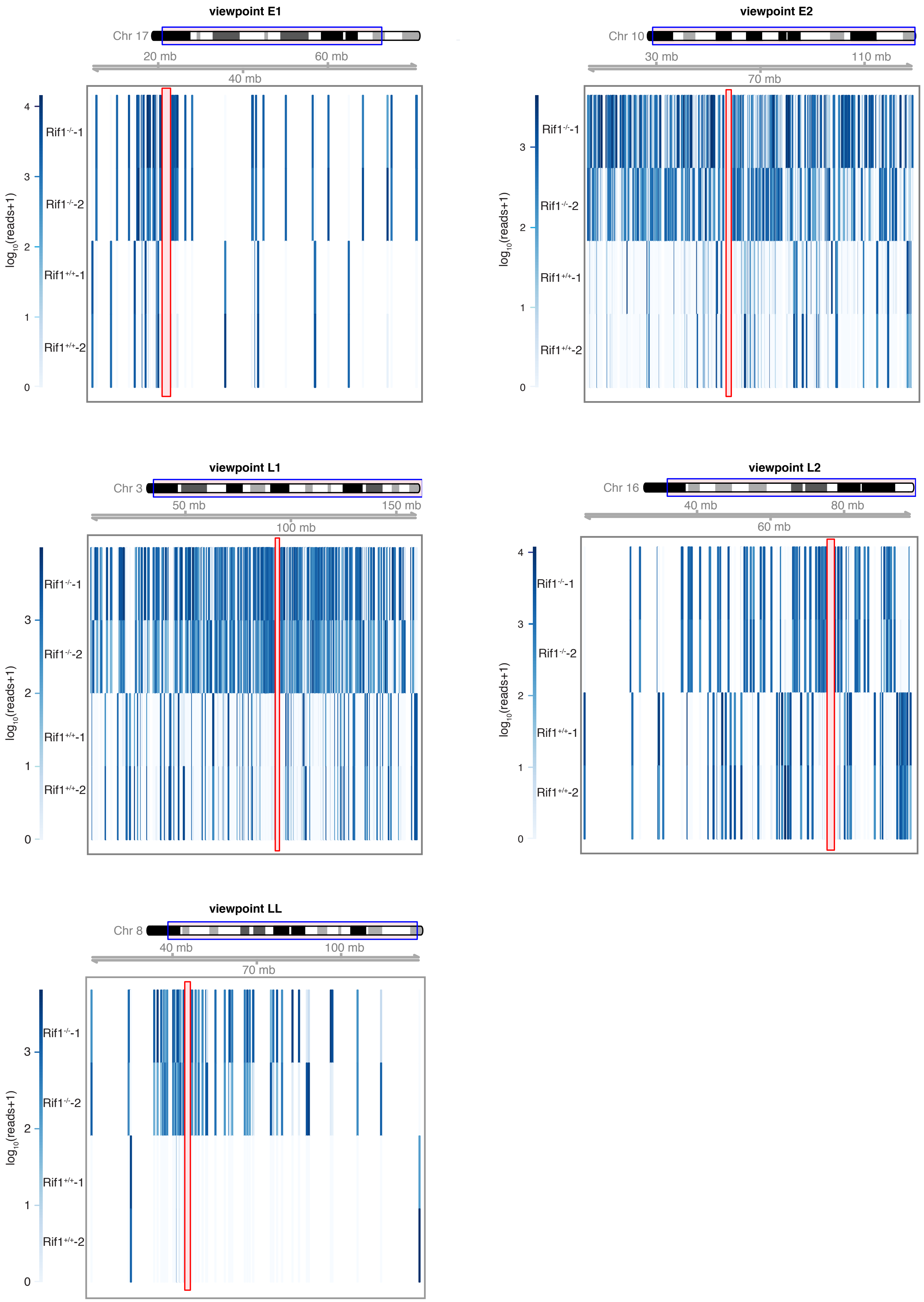
viewpoint E1
Chr 17
20 mb
60 mb
40 mb
log10(reads+1)
Rif1-/--1
Rif1-/--2
Rif1+/+-1
Rif1+/+-2
viewpoint E2
Chr 10
30 mb
110 mb
70 mb
viewpoint L1
Chr 3
50 mb
150 mb
100 mb
viewpoint L2
Chr 16
40 mb
80 mb
60 mb
viewpoint LL
Chr 8
40 mb
100 mb
70 mb

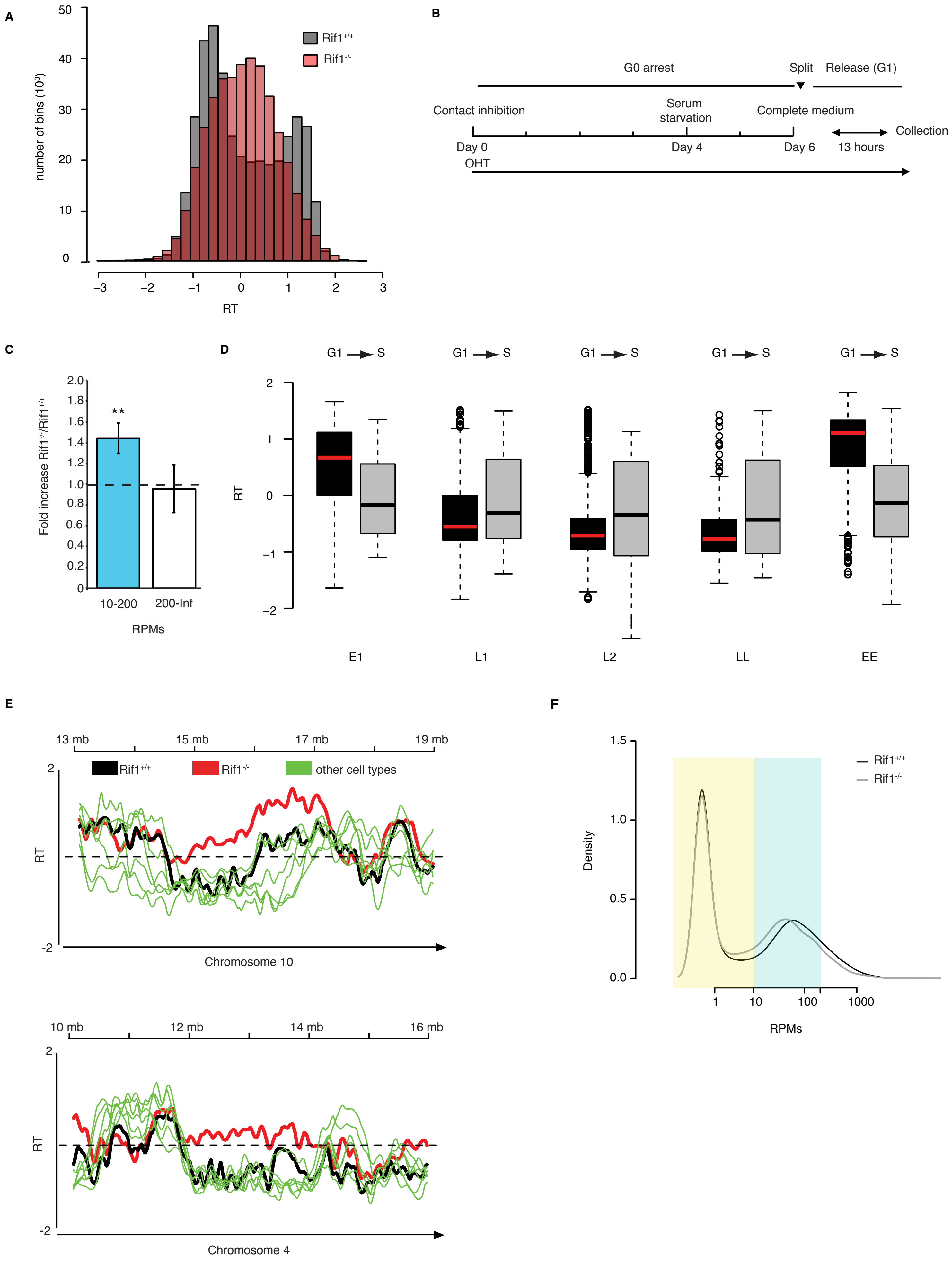
A
number of bins (10³)
Rif1+/+
Rif1-/-
RT
B
G0 arrest
Split
Release (G1)
Contact inhibition
Serum starvation
Complete medium
Collection
Day 0
Day 4
Day 6
13 hours
OHT
C
Fold increase Rif1-/-/Rif1+/+
**
10-200
200-Inf
RPMs
D
G1 S
RT
E1
L1
L2
LL
EE
E
13 mb
15 mb
17 mb
19 mb
Rif1+/+
Rif1-/-
other cell types
RT
Chromosome 10
10 mb
12 mb
14 mb
16 mb
RT
Chromosome 4
F
Density
Rif1+/+
Rif1-/-
RPMs

Supplemental Figure Legends

Figure S1: Rif1 null ESCs and LT-MEFs do not arrest upon Rif1 deletion and ESCs start differentiating upon a sustained DNA damage response. Related to Fig. 1. A Replication-timing plot of normalized, non-smoothed data from one representative Rif1$^{+/+}$ and one Rif1$^{-/-}$ ESC line. **B** To determine if the G1/S checkpoint function is responsible for the decreased proliferation of pMEFs in response to Rif1 deletion, we infected pMEFs with SV40 large-T antigen (LT) to abolish this checkpoint. Rif1$^{+/+}$ or Rif1 conditional (Rif1$^{F/F}$) cells infected with an empty (EV) or Cre-encoding (Cre) vector were compared. Shown are the averages from triplicates of three independent Rif1$^{+/+}$ versus Rif1$^{-/-}$ LT-MEFs, from one representative experiment out of two. Error bars indicate standard deviations. LT-immortalization alleviates the proliferation arrest of Rif1 deficient pMEF but instead activates the DNA replication checkpoint, as seen in ESCs (Buonomo et al., 2009). **C** Western blot showing p21 levels upon Rif1 deletion in six independent Rif1$^{-/-}$ and Rif1$^{+/+}$ cells lines four days after Cre induction. H2AX is used as loading control. pMEF treated with Mitomycin C (MMC) are used as positive control of p21 induction. Rif1 deletion in ESCs does not induce activation of the G1/S checkpoint, as monitored by p21 upregulation. **D** Analysis of cell cycle distribution from six independent Rif1$^{+/+}$ versus Rif1$^{-/-}$ ESCs. The results of three independent experiments are shown. Cells were pulsed for 1 hour with EdU, fixed and analyzed by FACS. Error bars represent standard deviations. t-test was used to calculate P values (***P*<0.001; *** *P*<0.0001). **E** Two and four days after Cre-induction cells were stained for Chk1^{Ser345} phosphorylated (green) and Oct4 (red). Insets show examples of single cells. Four days after Cre-induction Rif1$^{-/-}$ ESCs with higher levels of damage start down-regulating Oct4. **F** Distribution of the

Oct4 positive and negative ESCs among Chk1^{Ser345} phosphorylated positive population. 130 cells showing Chk1^{Ser345} phosphorylated signal were counted for Rif1$^{-/-}$ ESCs. For Rif1$^{+/+}$ the entire population of cells positive for Chk1^{Ser345} phosphorylated signal detectable on the slide was counted. **G** Western blot analysis of Oct4 and Nanog levels six days after Rif1 deletion in six independent Rif1$^{-/-}$ and Rif1$^{+/+}$ ESC lines shows that the loss of pluripotency becomes evident in the population at this later stage. These results explain the loss of pluripotency previously reported in long-term Rif1 knock-down ESCs (Dan et al., 2014).

Figure S2: Rif1 genome-wide distribution and lamin B1. Related to Fig. 2. A qPCR confirmation of selected Rif1 binding sites on two independent Rif1$^{FH/FH}$ and Rif1$^{+/+}$ ESC lines. The enrichment of the signal in the sample immunoprecipitated either by anti-HA antibody or by IgG control is normalized over the input. The primers were designed over three regions that showed enrichment of Rif1 in the ChIP-seq (Late 1 to 3) and two regions that did not (Early 1 and 2). **B** Enrichment analysis of Rif1 peaks in the indicated regions for one representative ESC line out of three analyzed. The expected values were calculated based on shuffled peaks. **C** Immunofluorescence for lamin B1 and Rif1. In Cyan EdU staining identifies S-phase cells. DAPI in blue. Scale bar=5μm. The bar chart shows the quantification of Rif1 enrichment at the nuclear periphery versus the interior. Quantification of the DAPI distribution has been used as negative control. The error bars indicate standard deviations. (***P<0.0001 was calculated by t-test). Ten different fields have been analyzed. **D** Rif1 immunoprecipitates lamin B1. IN=input, 1% of total extract; FT=flow through; IP=immunoprecipitated proteins. *=cross-reacting band. #= Rif1 degradation product. The red arrow indicates lamin B1 band. **E** Venn diagram indicating

the overlap in base pairs between LADs+RADs and late or early replicating regions. **F** Meta-analysis of Rif1 distribution (Rif1 enrichment=$\log_2$(Rif1 enrichment/input)) with respect to randomized regions (LADs shuffled over the genome). As for the LADs (Fig. 2D), an unsupervised clustering yields 4 distinct classes of random regions (with high or low Rif1 signal and the respective boundaries). However, compared to Fig. 2D, the cluster sizes are inverted, pointing to the preferential association of LADs with strong Rif1 signal. Notice that the RT-behavior of random regions is also markedly different from LADs. **G** Replication timing distribution (RT=$\log_2$(early/late)) is shown for Rif1$^{+/+}$ and Rif1$^{-/-}$ ESCs, with respect to the randomized regions and in the same order as in **F**.

Figure S3: Rif1 does not control gene expression directly. Related to Fig. 4. A For each UCSC gene the Rif1 signal with the shortest distance was determined. This plot shows the cumulative distribution of these distances. Notice that 20% of all TSS overlap directly with a peak (distance=0). Data from one representative out of three ESC lines analyzed. **B and C** Time course of gene expression changes upon Rif1 deletion in ESCs. Microarray analysis of total RNA two (**B**) and four days (**C**) after the induction of Rif1 deletion. Scatter plots show normalized expression values of RNA between four Rif1$^{-/-}$ and four Rif1$^{+/+}$ cell lines. Each of the red squares represents a transcript whose level varies more then two folds (log fold change >1) with an associated P<0.05. Only 0.7% and 2.5% of the Rif1-bound TSSs are associated with genes whose expression is affected by Rif1 deletion after two and four days of Cre-treatment respectively. **D** Heatmaps showing the distribution of Rif1-signal in a 10kb region around the TSSs of those genes whose expression is up-regulated (up) or down-regulated (down) upon Rif1 deletion (two and four days after Cre induction). The genes are sorted from the most (top) to the least

deregulated (bottom). Note that the top-deregulated genes indicated by the red boxes do not display Rif1 associated to the TSS. **E** Statistical analysis of the distribution of deregulated genes among GO categories from PANTHER. Table summarizing the GO categories most enriched among the differentially expressed genes. We could not identify any gene ontology (GO) category-specific enrichment suggestive of a link between gene expression profile changes and the DNA damage response or differentiation processes.

Figure S4: Rif1 deletion does not immediately impact on the epigenetic landscape. Related to Fig. 4.

A-D Spearmann correlation plots of the genome-wide distribution of H3K4me3 (**A**), H3K9me3 (**B**), H3K27me3 (**C**), H4K20me3 (**D**) calculated comparing data from two Rif1$^{+/+}$ and two Rif1$^{-/-}$ lines. **E-G** Heatmaps showing +/- 5kb around TSSs the distribution for H3K4me3 (**E**), H3K9me3 (**F**) and H3K27me3 (**G**) respectively in one representative Rif1$^{+/+}$ and Rif1$^{-/-}$ ESC lines out of two analyzed. TSSs are stratified accordingly to Rif1 presence and clustered on the basis of the association with genes differentially affected upon Rif1 deletion.

Figure S5: Rif1 deletion does not affect H3K4me3 distribution around SNSs or H3K9me3 and H4K20me3 enrichments at major satellites, but increases the number of chromatin contacts. Related to Fig. 4, Fig. S3 and Fig. 5. **A** Heatmaps showing H3K4me3 distribution +/- 5kb around SNSs mapped on chromosome 11 in one representative Rif1$^{+/+}$ and Rif1$^{-/-}$ line out of two analyzed. SNSs were clustered accordingly to their replication timing. **B and C**. ChIP-qPCR quantification of H3K9me3 (**B**) and H4K20me3 (**C**) levels at major satellites in Rif1$^{-/-}$ and Rif1$^{+/+}$ ESCs. The average of three ESC lines per genotype in two independent experiments is shown. The error bars

represent standard deviations. **D** LT-MEF deficient for Rif1 function display mildly de-regulated gene expression. Microarray analysis of total RNA from three independent Rif1$^{F/F}$ lines infected with either Cre-encoding or empty vectors. The scatter plot shows normalized expression values of RNA. Each red square represents a transcript whose level varies more then two folds (log fold change >1) with an associated P<0.05 between Rif1 wild type (Rif1$^{F/F}$+EV) and null (Rif1$^{F/F}$+Cre) cells. **E** Ratio (fold increase) of the average number of fragments containing the indicated RPM's ranges in two individually considered Rif1$^{-/-}$ versus two Rif1$^{+/+}$ (dashed line) ESCs calculated over all the viewpoints. The increase of fragments containing 10-200 RPMs in Rif1$^{-/-}$ is statistically significant as calculated by two ways ANOVA test based on conditions and viewpoints (*P=0.04). The error bars indicate standard deviations. In this figure all fragments which belong to either class (10-200 or above 200RPMs) are used for the calculation, while Fig. 5E is based on those interactions consistently identified in all replicates within a condition as calculated by the r3Cseq software package. **F** 3C-qPCR confirmation of four selected interactions identified by 4C-Seq for two viewpoints. In each case Rif1$^{-/-}$ ESCs show increased contacts, confirming the 4C-Seq results. The data were obtained from two independent Rif1$^{+/+}$ and Rif1$^{-/-}$ ESC lines. The Rif1$^{-/-}$ enrichment normalized over the input is expressed as fold increase over the normalized Rif1$^{+/+}$ enrichment. **G** Analysis by 3D FISH of the average distance between the two probes indicated in the Supplemental Experimental Procedures, one on the viewpoint E1 on chromosome 17 and the other on one of its most frequent interactions gained in Rif1$^{-/-}$ ESCs (*P=0.0125). Analysis of the average distance between these two probes in Rif1$^{-/-}$ versus Rif1$^{+/+}$ ESCs has confirmed the results obtained by 4C-Seq, showing a decreased average distance between the

viewpoint E1 and the distal interacting point. The analysis was performed blindly and the error bars indicate the standard error median. Wilcoxon test was used to calculate the P value. **H** Relative distribution of the fragments containing the indicated RPMs obtained by combining all ESCs $Rif1^{+/+}$ (black line) and $Rif1^{-/-}$ (grey line) reads, over all the viewpoints, normalizing the library size to 1 million. The bimodal distribution allows the identification of 10 RPMs as cutoff to exclude interactions falling within the noise range (yellow shadowing). The blue shadowing corresponds to the 10-200 RPMs interval shown in blue in **B**.

Figure S6: Rif1 deletion increases the number of chromatin contacts. Related to Fig. 5. Heat maps showing the $\log_{10}$(reads+1) of the positions (blue lines) identified as significantly different between two $Rif1^{+/+}$ and two $Rif1^{-/-}$ ESC lines from the FourCSeq software (FDR < 0.1 and p-value < 0.001, cf. supplemental materials). The 2Mb region around the viewpoint is indicated in pink.

Figure S7: Failure to re-express Rif1 in G1 leads to nuclear architectural changes prior to S-phase. Related to Fig. 6. A Average genome-wide distribution of replication timing, RT score=$\log_2$(early/late), from Nimblegen tiling arrays with average tile size of 50bp. Shown is the distribution for $Rif1^{+/+}$ and $Rif1^{-/-}$ in pMEFs during the first S-phase after Rif1 deletion. **B** Scheme of the experimental setting employed. OHT=4-hydroxytamoxifen. **C** Ratio (fold increase) of the average number of fragments containing the indicated RPM's ranges in two individually-considered $Rif1^{-/-}$ versus two $Rif1^{+/+}$ (dashed line) pMEFs calculated over all the viewpoints. The increase of fragments containing 10-200 RPMs in $Rif1^{-/-}$ is statistically significant as calculated by two ways ANOVA test based on conditions and viewpoints (***P*=0.009). The error bars indicate

standard deviations. In this figure all fragments which belong to either class (10-200 or above 200 RPMs) are used for the calculation, while Fig. 6C is based on those interactions consistently identified in all replicates within a condition as calculated by the r3Cseq software package. **D** Boxplot showing the distribution of replication-timing values of the interactions within the TADs shared between Rif1$^{+/+}$ and Rif1$^{-/-}$ for the indicated viewpoints. The black boxplots show the distribution of the replication timing of the shared chromatin interactions plotted against the Rif1$^{+/+}$ replication timing. As expected, these interactions fall in the same replication timing as the viewpoint. The gray boxplots show the distribution of the replication timing of the shared chromatin interactions plotted against the Rif1$^{-/-}$ replication timing. The shift of the median indicates that the switch of replication timing of the viewpoint in the first S-phase after Rif1 deletion is concomitant with replication timing shifts of only a portion of the interacting TADs. **E** Exemplary plots of replication-timing changes (RT=$\log_2$(early/late)) between one Rif1$^{+/+}$ and one Rif1$^{-/-}$ pMEFs during the first cell cycle (Cornacchia et al., 2012) along with corresponding replication-timing profiles in other published cell types (http://www.replicationdomain.com/). **F** Relative distribution of the fragments containing the indicated RPMs obtained by combining all pMEFs Rif1$^{+/+}$ (black line) and Rif1$^{-/-}$ (grey line) reads, over all the viewpoints, normalizing the library size to 1 million. The bimodal distribution allows the identification of 10 RPMs as cutoff to exclude interactions falling within the noise range (yellow shadowing). The blue shadowing corresponds to the 10-200 RPMs interval shown in blue in **C**.

Table S1

	Early	Late	Undefined RT	TOTAL
**TSS/no Rif1 peak**	15,131	7,297	1,777	24,205
**TSS/overlapping Rif1 peak**	3,171	2,522	328	6,021
**TSS**	18,302	9,819	2,105	30,226

Table S2

Primer	Sequence 5' – 3'	Location (mm9)	Description
P1 fwd	ACTTAGGGCAGCCCAATTCC	chr5:45840960-45841085	Late 1
P1 rev	TAGGTGTTCCCTAGGCCTCC		
P2 fwd	AGAACTGGAGGAACGCCTTG	chr3:93247689-93247796	Late 2
P2 rev	TCCTGCCTCTCTTGCCTTTG		
P3 fwd	TGCTCTCGCTTGGTGACATT	chr6:21164727-21164854	Late 3
P3 rev	AGGTTAGGCAAAGGCGTCTC		
D1 fwr	TCCAAAGGGCTTGCATCACT	chr5:31465642-31465751	Early 1
D1 rev	ATGCAGTGCTATGGGGTCAC		
D2 fwr	GACCACGTTTCTTGCCGTTC		Early 2
D2 rev	GCAGTGATGTCCACAAGGGA	chr2:119252415-119252507	

Major satellite Primers	Sequence 5' – 3'
MajSatFwd	GACGACTTGAAAAATGACGAAATC
MajSatRev	CATATTCCAGGTCCTTCAGTGTGC

Table S3

Illumina adaptors:

P5: 5'-AATGATACGGCGACCACCGAGATCTACACTCTTTCCCTACACGACGCTCTTCCGATCT-3'

P7: 5'-CAAGCAGAAGACGGCATACGAGATCGGTCTCGGCATTCCTGCTGAACCGCTCTTCCGATCT-3'

ESC viewpoints	HindIII	DpnII
E1	**P5-CCGTAT** AGCTTCTTGCTGTCAAGCTT	**P7-**TGGAGTGCTGATGAAAGTCA
E2	**P5-TGAGTG** GTGTTCTTGGTTGGAAGCTT	**P7-**GCCCAGCACCATAGAAACTG
L1	**P5-CATTCA** TCCCTCGCTCTCACAAGCTT	**P7-**CCCTCATAGAAGGTGCCATT
L2	**P5-ATTATA** AAACCCAACTTGCCAAGCTT	**P7-**CACGATGTGCTTCCTGTCAT
LL	**P5-GGCCAC** TGGATAGTGGTCTAAAGCTT	**P7-**GGCTCCAGTGACATCCATG

pMEF viewpoints	HindIII	DpnII
E1	**P5-GGCCAC** GCCCAGCTTGTTCGAAGCTT	**P7**-CGTAAGGGTTGGTGAGATGG
L1	**P5-CATTCA** TGTGGCATGTATGGAAGCTT	**P7**-CTCAGTGGAGCCAAGAAACC
L2	**P5-GAGAGT** CCAGAAGGTTCTTACAAAGCTT	**P7**-CTCAGTGGAGCCAAGAAACC
LL	**P5-CCGTAT** AGCTTCTTGCTGTCAAGCTT	**P7**-TGGAGTGCTGATGAAAGTCA
EE	**P5-TGAGTG** CAGTTAGCAAGGACCAAGCTT	**P7**-CCTGGTGGCAGAGAACTTTA

Table S4

Primer	Sequence 5'- 3'	Location interaction (mm9)	Description
Int. 1 fwd	TGTTTATAAGCCCTCAGGTACATA	chr17:21762323 -21772199	Viewpoint E1
Int. 1 rev	GACAAATCACAACAGGAGAAGTAATAG		
Int. 2 fwd	ATTCCTATGTTTATAAGCCCTCAG	chr17:21714258 -21717665	Viewpoint E1
Int. 2 rev	AGCGGTCCTTGCCAATTATG		
Int. 3 fwd	ATTCCTATGTTTATAAGCCCTCAG	chr17:21096804 -21100021	Viewpoint E1
Int. 3 rev	AGCTAGAGCTATTTCAGTGGAAAC		
Int. 4 fwd	TCAGGAGACAACTTCGTGGAG	chr16:77299025 -77301113	Viewpoint L2
Int. 4 rev	TGAAAAGATGAAGAGGCAAAGG		
Rnr2-1*	TAGGGATAACAGCGCAATCC		Gen. DNA quant.
Rnr2-2*	GACTTTAATCGTTGAACAAACGAAC		
XBP1-1**	GCCCTCCCTGAAAATAAGGA		Ligation control
XBP1-2**	GACTTCTCACCTGGGCCTACA		
HindIII-neg fwd	CCCATTCCTGAGGGTTAGCA		Gen. DNA quant.
HindIII-neg rev	GAAGGAGTGAGAACCGTGCA		
HindIII fwd	GTCATGCTGCCACAGTCTCT		Digestion efficiency
HindIII rev	CCTTCAAGGTTCAGGGGACT		

Taqman probes	Sequence
E1	5'FAM-AAGCTTGACAGCAAGAAGCTCTGGTTT-3'TAMRA
L2	5'FAM-AAGCTTGGCAAGTTGGGTTTAATCTCC-3'TAMRA
RNR2*	5'FAM-CGACCTCGATGTTGGATCAGGACATCC-3'TAMRA
XBP**	5'FAM-AAAGCTTGCACCCTGCTTTAGTGGCC-3'TAMRA

BAC Viewpoint	BAC Interaction
E1: RP23-355H20	1: RP23-234E12
E1: RP23-355H20	2: RP23-234E12
E1: RP23-355H20	3: RP23-209P21
L2: RP23-323D13	4: RP23-325L9

*: Medvedovic, J. *et al*. Flexible long-range loops in the VH gene region of the Igh locus facilitate the generation of a diverse antibody repertoire. *Immunity* **39**, 229-244 (2013).

: Splinter, E. *et al*. The inactive X chromosome adopts a unique three-dimensional conformation that is dependent on Xist RNA. *Genes & development* **25, 1371-1383 (2011).

Supplemental Table Legends

Table S1: Related to Fig. S3. Contingency table for occurrences of 30,226 TSS in replication-timing domains, with/without overlapping Rif1 signal in one representative out of three lines analyzed. We observe 25% (2,522/9,819) of TSS embedded in late replicating domains and 17.3%. (3,171/18,302) in early being covered by a Rif1 signal. Therefore the association of Rif1 to late TSSs shows a slight but significant enrichment ($P<10^{-34}$) compared to the genome-wide expectation of 20%. Similarly, the fraction of TSS with a peak is higher in late domains (41.9%=2,522/6,021) compared to the expectation (32.4%=9,819/3,0226).

Table S2: Related to Fig. S2. List of the primers used for the ChIP validation by qPCR. Primers were designed on region with different Rif1 intensities and non-repetitive. The primers for the qPCR validation of the modified histones ChIPs are from (Lehnertz et al., 2003).

Table S3: Related to Experimental Procedures. List of the primers used for 4C-Seq library preparation. Sequences of the standard Illumina primers are reported (P5 and P7) and are the same for each of the viewpoints specific primers. The tables summarize the sequences of the viewpoint specific part of the primers. In bold is the barcode employed for multiplexing during sequencing. HindIII=forward primers; DpnII=reverse primers.

Table S4: Related to Experimental Procedures and Fig. S5. List of the primers and Taqman probes used for the 3C-qPCR. Primers were designed on the selected interactions indicated. List of BACs used to build control libraries for testing the primers.

Supplemental Experimental Procedures

Mouse lines

$Rif1^F$ and $Rif1^{FH}$ mouse lines were described respectively in (Buonomo et al., 2009; Cornacchia et al., 2012). The $Rif1^F$ mice were crossed with the strain B6;129 gt(ROSA)26Sor$^{tm1(cre/Esr1)Nat}$/J (Jackson laboratory) to introduce the inducible Cre.

Derivation of embryonic stem cell lines

Derivation of ESCs was carried out according to the protocol described by (Bryja et al., 2006) with the following changes. Blastocyst outgrowth was cultured on passage 1 MMC-treated pMEFs (feeders) plated at a density of about 350,000 cells/cm^2. During the first passages disaggregation was performed with 0.25% trypsin. Once established, ESCs colonies were expanded using 0.05% trypsin, on feeders plated at a density of about 175,000 cells/cm^2 in ES medium=Knockout-DMEM (Gibco 10829-018), containing 15% heat-inactivated fetal bovine serum, 1% Penicillin/Streptomycin (Gibco 15070063), 1% L-Glutamine (Gibco 25030-081), 1% non-essential amino acids (Gibco 11140-050), 0.1mM 2-Mercaptoethanol (Gibco 31350-010) and supplemented with 20 ng/ml leukemia inhibitory factor (LIF, EMBL Protein Expression and Purification core facility). Gelatin adaptation was performed by thawing ESCs frozen with feeders directly onto gelatinized plate (0.1% bovine gelatin Sigma G9391 in PBS) in ES medium, supplemented with 1μM MEK inhibitor PD0325901 and 3μM GSK3 inhibitor CH99021 (The University of Dundee, Dept. of Biochemistry, Medical Sciences Institute). ESCs were then cultured until stable growth was achieved. Cells were cultured at 37°C in 7.5% CO_2.

Cell manipulations

Derivation of pMEFs and synchronization experiments were carried out according to (Cornacchia et al., 2012). Immortalization by Large T antigen, infection with retroviruses carrying empty vector or Cre and growth curves were all performed as described in (Buonomo et al., 2009). Time courses of Rif1 deletion by Cre induction in ESCs were done as follows. Gelatin-adapted ESCs were freshly thawed and cultured for two passages prior to deletion. At the third splitting, 200nM 4-hydroxytamoxifen (OHT, Sigma H7904) was added to the medium. Medium was then replaced every two days with freshly added OHT. ESCs proliferation was evaluated by dividing the number of cells collected from duplicate wells for each of the six clones two and four days after plating by the number of cells plated at day 0. The experiment was repeated three times. LT-MEFs growth was evaluated by dividing the number of cells collected in triplicate wells at four and six days after plating by the number of cells plated at day 0. Two $Rif1^{+/+}$ and three $Rif1^{F/F}$ LT-MEF lines infected by a Cre-encoding retrovirus were used. The MTT assay (Roche 1465007) was performed according to the manufacturer instructions, plating 500 cells/well of a 96 well plate in quadruplicate for all six clones. The quantitative alkaline phosphatase assay (Millipore SCR066) was performed according to the manufacturer instructions, plating 20,000 cells/reaction in triplicate for two clones. The experiment was performed once.

Replication timing

The analysis was performed as described in (Ryba et al., 2011). Briefly, asynchronously cycling cells were pulse labeled with the nucleotide analog 5-bromo-2-deoxyuridine (BrdU). The cells were sorted into early and late S-phase fractions on the basis of DNA content using flow cytometry. BrdU-labeled DNA from each fraction was

immunoprecipitated with an anti-BrdU antibody, amplified, differentially labeled and co-hybridized to a whole-genome comparative genomic hybridization microarray. The data from each channel was normalized using Limma package (Smyth, 2004). The log_2(early/late) value was smoothed and plotted against the position along the chromosome to generate RT profiles. Comparison of replication-timing profiles derived from different cell types allows the identification of replication-timing domains defined as the unit of regulation of replication timing (400-800 kb) (Takebayashi et al., 2012). In this comparison replication-timing domains will coincide with the unit of replication-timing changes between cell types. This also highlights that when the changes in replication timing resulting from Rif1 deletion encompass more than a megabase, likely multiple adjacent smaller domains with similar replication timing are being affected. Percentage of replication timing changes in Fig. 1B is calculated as the number of probes on the array that change by a factor more then 1 versus the total number of probes. The genome-wide distributions of replication timing in Fig. 1C and Fig. S7A were calculated directly from the RT-scores on each tile of the array. For ES cells the tiles have a size of 60bp, for pMEF cells the average size is 50bp. Replication timing data are available in the ArrayExpress database (www.ebi.ac.uk/arrayexpress) under accession number E-MTAB-3506.

Transcriptome analysis

Total RNA was isolated using the TRIzol reagent (Invitrogen #15596018) following the manufacturer's instructions and treated with DNase (Promega RQ1 RNase-Free DNase: M6101). RNA quality was assessed on the Agilent 2100 Bioanalyzer (Agilent Technologies) using the RNA6000 Nanokit (#50671511 Nano), and RNA quantity was

measured with ND-1000 NanoDrop spectrophotometer. 1μg of RNA sample was used for microarray analysis on Affymetrix Mouse Gene 1.0ST array (Affymetrix). Robust multi-array average (RMA) normalization was applied. Normalized data were then filtered based on the Affymetrix detection call so that only probes that had a Present call in at least one of the arrays were retained. The transcriptome analysis of LT-MEF was then performed as in (Cornacchia et al., 2012). In brief, cells were infected four times every 12 hours with either a Cre encoding retrovirus or empty vector, then selected for four days with hygromycin 90 μg/mL and collected for analysis. Deregulated genes whose expression is changed as a consequence of Cre expression rather then Rif1 deletion have been excluded by parallel microarray analysis of RNA from three Rif1$^{+/+}$ lines infected with either Cre-encoding (Cre) or empty vectors (EV). CEL files were imported in GeneSpringGX 11.5 software and expression values were filtered by percentile (20-100). t-test with an asymptotic P value and no correction was applied for statistical analysis. ESCs transcriptome analysis was performed in R using Limma package (Smyth, 2004). In both cases, a fold change cutoff of 2 was applied to detect the significantly differentially expressed genes. Microarray data are available in the ArrayExpress database (www.ebi.ac.uk/arrayexpress) under accession number E-MTAB-3503 for the ES cells and accession number E-MTAB-3501 for LT-MEF.

ChIP

Chromatin immunoprecipitation was performed accordingly to (Bulut-Karslioglu et al., 2012) with the following modifications. For Rif1 ChIP, Rif1$^{FH/FH}$ and Rif1$^{+/+}$ ESCs were harvested from four 15cm dishes at 80% confluency. Cells were first cross-linked using 2mM disuccinimidyl glutarate (DSG Synchem UG & Co. KG # BC366) in PBS for 45

min. at room temperature while rotating, washed twice in PBS, followed by 10 min. of additional crosslinking in 1% formaldehyde. After 5 min. quenching in 0.125M glycine at room temperature, chromatin fragmentation was performed using either a Covaris S2 or Vibrocell VCX 400 to produce a distribution of fragments enriched between 200 and 600bp. Chromatin was quantified by ND-1000 NanoDrop spectrophotometer. Immunoprecipitation was performed with anti-HA antibody (Roche monoclonal 3F10 # 11867423001) using 0.5μg of anti-HA antibody and 50μl of Dynabeads protein G (Invitrogen 100.03D) per chromatin corresponding to 25μg of DNA. The reason the anti-FLAG antibody was not used in parallel is because the double crosslinking process renders the epitope unrecognizable for the antibody.

For modified histone's ChIP-seq 2 $Rif1^{-/-}$ and 2 $Rif1^{+/+}$ ESC lines were harvested from one 15cm dishes 2 days after Rif1 deletion was induced. Cells were only cross-linked in 1% formaldehyde. Chromatin fragmentation was performed using either a Covaris S220 to produce a distribution of fragments enriched between 200 and 500bp. Chromatin was quantified by Qbit (Life Technologies Q32851). Immunoprecipitation was performed by incubating 5μg of chromatin over night in the cold with either anti-H3K4me3 (0.2μg), or anti-H3K9me3 (2.4μg), or anti-H3K27me3 (6μg), or anti-H4K20me3 (0.4μg) antibodies or IgG and 30μl of Dynabeads protein G (Invitrogen 100.03D) per ChIP. Retrieved DNA was quantified by Qbit (Life Technologies Q32851). From the control immunoprecipitation ($Rif1^{+/+}$ ESCs for Rif1 and IgG for histone ChIPs) was not possible to retrieve enough DNA to produce a library. The primers used for qPCR validation of the ChIP data are indicated in the Table S2 (Lehnertz et al., 2003).

ChIP sequencing and analysis

ChIP-seq libraries were prepared with NEBNext ChIP-Seq Library Prep Master Mix Set for Illumina (NEB#E6240S/L), except that bead purification was employed (Agencourt AMPure XP beads, Beckman Coulter A63881). Size selection was performed on 2% agarose gels (E-Gel Invitrogen G6610-02). For Rif1 ChIPs, generally 13 PCR cycles for 10ng starting material were applied. We titrated the number of cycles in order to obtain the best quality libraries and set on 13 because is the number that gave the least amount of PCR artifacts (duplication rate). Libraries were barcoded with the NEBNext Multiplex Oligos for Illumina (Index Primers Set 1, NEB#E7335S/L).

The paired-end reads of all ChIP-seq samples were mapped with bowtie2 ((Langmead and Salzberg, 2012) version 2.0.5) against the mouse genome mm8 with a maximum fragment length of 1000bp. MACS (https://pypi.python.org/pypi/MACS2) (with the --broad option) was used to define specifically enriched regions for Rif1. To correlate the Rif1 peaks with other genomic annotations, we determined the number of enriched regions overlapping with replication domains, cLADs and ciLADs (Meuleman et al., 2013), exons and intergenic regions obtained from the UCSC table browser. The expected overlap and the variance for a given annotation were estimated from 50 random sets of shuffled regions with the same number of features and the same extent. A standard Z-score was calculated; Z=(expected-observed)/(standard deviations). Since the peak calling alone does not fully reflect regions of broad Rif1-enrichment, we also utilized the EDD tool (Lund et al., 2014) with default parameters to detect larger domains. Independently of peak and domain calling, we calculated genome-wide profiles of Rif1 enrichment, $\log_2$(ChIP/Input), for windows of 50bp and normalized with respect to

different sequencing depths. These profiles provide the basis for visualization and downstream analysis with deepTools (Ramirez et al., 2014) to determine the behavior of Rif1-enrichement around selected genomic features, such as the origins of replications.

Rif1 ChIP-seq data are available in the ArrayExpress database (www.ebi.ac.uk/arrayexpress) under accession number E-MTAB-3502. Modified histone's ChIP-seq data are available under accession number E-MTAB-3743.

Models for Origin of Replications

The small nascent strands (SNSs) were obtained from the deori database ((Gao et al., 2012) version 2.1), which includes all 2405 SNSs on chromosome 11 from the study by Mechali et al. (56613063-117015900; mm8) (Cayrou et al., 2011). Based on the domain prediction from replication timing data, the SNSs were classified into 1873 early-replicating SNSs (RT>0.5) and 532 late-replicating SNSs (RT<-0.5). 1000 random regions were chosen as control. To supplement the analysis of origins with a motif-based analysis, we obtained the annotation of two complementary models (G4s and OGREs) from the Mechali's group (personal communication, (Cayrou et al., 2012)). We identified all the motif instances and examined their occurrence in flanking region of +/- 4kb around TSSs and SNSs. DeepTools (Ramirez et al., 2014) were used to visualize and summarize Rif1 data and other features around TSSs and SNSs. We generally chose flanking regions of +/-5 kb, except for replication-timing data that are defined for much larger domain. We therefore chose in this case much larger flanking region of +/- 500kb. In most cases we use predefined groups of feature depending on the replication-timing

status, but in Fig 5F and G we used unsupervised clustering (kmeans with k=2) to distinguish CpG-high and CpG-low regions.

LAD

The Lamin B1 associated domains (LADs) for mouse ESC were obtained from the supplementary material of (Peric-Hupkes et al., 2010). For randomized analysis we also generated a shuffled version of these locations with an identical size distribution and excluding original instances of LADs. Using DeepTools ((Ramirez et al., 2014)) we generated genome-wide profiles and heatmaps as for other genomic features (SNSs, G4), but here we added a flanking region of +/- 0.2 Mb. The unsupervised clustering of the heatmaps was done based on the Rif1-data and the replication time was shown in the same order.

Rif1 ChIP and RT change analysis

Replication timing and Rif1 ChIP data were averaged into 200kb windows and LAD score were calculated for the windows by using the predict function in R. Windows that have RT above 0.5 were defined as Early (E) and below -0.5 were defined as Late (L). Rif1 ChIP enrichment was plotted against replication timing differences ($\Delta RT = Rif1^{-/-} - Rif1^{+/+}$) for windows that either remain Early/Late in both $Rif1^{+/+}$ and $Rif1^{-/-}$ ESCs (EtoE and LtoL) or for regions that switch replication timing upon conditional deletion of Rif1 (EtoL and LtoE; $\Delta RT>+1$ and $\Delta RT<-1$). Lamin B1 association distribution for EtoE, LtoL, EtoL and LtoE were represented as boxplots.

4C-Seq

Chromosome conformation capture was performed as described in (Splinter et al., 2012; Stadhouders et al., 2013) with the following changes. 1 or 2 x10^7 cells (ESC and pMEF respectively) were collected for each experiment and cross-linked in 2% formaldehyde. Cell were lysed for 20 min. at 4°C, followed by addition of 0.3% SDS. After 10 min. at 65°C, the lysates were incubated for 1 hour at 37°C. For the first restriction digest, 1000U of HindIII in total were added for two overnight, distributed as follows: 200U for 4 hours, 200U for the first overnight, 200U for 4 hours, 200U for the second, 200U for the last 4 hours. Before the ligation step, HindIII was heat-inactivated. Following steps have been performed as described in (Splinter et al., 2012). DpnII restriction enzyme was chosen as four-cutter. Synchronized MEFs DNA was digested for two over nights by DpnII. PCR reaction was performed as described in (Splinter et al., 2012), except that Takara Ex Taq DNA Polymerase Hot-start version (Takara RR006B) and 600ng of total template in three reactions of 50 µl each were used. The primers employed to build the library are indicated in the Table S3. Six samples per lane were pooled according to molarity and loaded on a HiSeq 2000 in 100bp single-end mode. Analysis of the 4C-Seq has been performed as described in (Thongjuea et al., 2013). In brief, sequence reads were mapped to the genome. Unaligned reads were tested for the restriction enzyme cutting site and if applicable trimmed and mapped again. Uniquely mapped reads were used for downstream analysis. The number of reads per restriction fragments was counted by applying the method “WholeReads” (Thongjuea et al., 2013). Regions with less then 50 reads were removed from the analysis first. The number of remaining reads was then normalized “PowerLaw fitting curve” (Thongjuea et al., 2013). The normalized number of reads is indicated as RPMs and it is a measure of how many times each interaction is

detected in the library (frequency). Each restriction fragment with at least one RPM and shared by both biological replicates was define as an interaction ("intersection method" (Thongjuea et al., 2013)). After the intersection, we have additionally eliminated from our following analysis any interaction represented by less then 10 RPMs. This cutoff was established based on the distribution of the RPMs in each individual replica and for each of the viewpoints. In Fig. S5H (ESCs) and Fig. S7F (pMEFs) the cumulative curves of RPMs distribution obtained by averaging the biological duplicates across all the viewpoints are shown as representative summary of the behavior of each individual sample. By employing this method we define the significance of the interactions by their presence in all the biological replicas, followed by their frequency above a background established on the individual samples before intersection, to increase its stringency. This approach, rather then applying a statistical threshold, allows us to take into account also lower frequency interactions. The total number of interactions is calculated as the sum of all restriction fragments showing at least 10 RPMs. The number of interactions inside the replication domain corresponding to each of the viewpoint has been calculated as the sum of restriction fragments showing at least 10 RPMs within the coordinates of the border of the replication-timing domain. A replication-timing domain is defined as a continuous chromosomal region showing the same replication timing. For most of the viewpoints this corresponds also to the replication-timing domains as defined by alignment (ENCODE database on http://genome.ucsc.edu) with exception of viewpoint L1 and E1 for ESCs and L2 in MEFs. In these regions the replication-timing alignment data are not sufficiently clear to define the presence of eventual subdomains. In these cases we therefore considered a single domain the whole region displaying the same replication

timing within 1Mb. 4C-seq data for both ES cells and LT-MEF are available in the ArrayExpress database (www.ebi.ac.uk/arrayexpress) under accession numbers E-MTAB-3500 and E-MTAB-3505 respectively.

4C-Seq and RT changes analysis

The list of topological associating domains (TADs) for mouse ESC was obtained from (Dixon et al., 2012). The interactions *in cis*, outside of the replication domain and with at least 10 RPMs were intersected with TADs. On this basis, interactions present in TADs that are common between Rif1$^{+/+}$ and Rif1$^{-/-}$ were classified as shared, irrespective of the specific fragend mapped. Each TAD contains indeed multiple fragends. TADs containing exclusively Rif1$^{-/-}$-specific interactions were classified as Rif1-null specific. RT score was then assigned to the interactions grouped as above as described in (Ryba et al., 2011).

3C-qPCR

The 3C library was prepared as for the 4C, but without DpnI digest and following ligation. The quality controls and the qPCR assays were performed according to (Hagege et al., 2007). Primers were designed over four different interactions mapped in two independent viewpoints in ESCs. The list of the primers and Taqman probes employed is in the Table S4. The BACs used to build control libraries are also listed in Table S4.

FourCSeq analysis

The 4C-Seq data were also analyzed according to the FourCSeq Bioconductor software package (see http://bioinformatics.oxfordjournals.org/content/31/19/3085). The FourCSeq software uses calibrated and variance-stabilized read count data as input for

the statistical test to identify fragments on the in cis chromosome which differ between the conditions. Sequence reads were mapped to the genome. Unaligned reads were tested for the restriction enzyme cutting site and if applicable trimmed and mapped again. Uniquely mapped reads were used for downstream analysis. In the filtering step fragments with an average read count of at less than 5 RPMs across all samples and fragments within 10000 nucleotides of the viewpoint were removed. To fit the decay trend the asymmetric monotone fit option was chosen. To determine statistically different fragments between the two conditions a threshold of 0.001 for the P-value and of 0.1 for the FDR were applied.

Immunofluorescence and image analysis

Rif1$^{FH/FH}$ ESCs were grown on gelatinized coverslips over-night. Cells were fixed in 4% paraformaldehyde in PBS for 10 min. at room temperature and stored at 4°C in PBS and Sodium Azide. For staining, coverslips were permeabilized in 0.5% Triton X-100 at room temperature for 10 min., and then processed as described in (Buonomo et al., 2009), except that the primary antibodies were incubated 90 min. Images were acquired using a Leica confocal TCS SP5 microscope with a X63 1.4 NA oil objective and run by LAS AF Software (Leica). Contrast adjustment and cropping were performed in Image J. Figures were composed in Illustrator (Adobe). The image analysis of Rif1 enrichment at the nuclear periphery was performed using the free open-source software CellProfiler (Carpenter et al., 2006). In brief, we segmented the whole-nuclear area by a manual threshold of the DAPI image and the nuclear-periphery region by a manual threshold of a top-hat filtered lamin B1 image. We then defined the nuclear-interior by removing the nuclear-periphery mask from the whole-nucleus mask. We measured the mean Rif1 and

DNA intensities in the nuclear periphery and the nuclear interior and computed the ratio. In order to control for the quality of the input data, we only took into account images were at least 95% of all the pixels in the nuclei region showed no saturation.

Protein extracts and Immunoprecipitation

For Fig. 1A, S1C and G proteins were extracted and analyzed as described in (Buonomo et al., 2009).

For Fig. S2D nuclei from $Rif1^{FH/FH}$ and $Rif1^{+/+}$ ESCs were resuspended in cold Benzonase buffer (50mM Tris-HCl pH 8.0, 100mM NaCl, 1.5mM MgCl2, 10% glycerol, freshly added protease and phosphatase inhibitors), subjected to snap freezing, followed by Benzonase digestion (50U/ml, Sigma, E1014). The nuclear solubilized fraction was collected by centrifugation and supernatants were used for immunoprecipitation as follows. 10ml $Rif1^{FH/FH}$ and $Rif1^{+/+}$ extracts were incubated with 8μl/ml anti-HA and anti Flag antibodies overnight at 4°C. 1% Input was collected prior antibody addition. Extracts were incubated with 100μl/ml Protein G Dynabeads (Invitrogen 100-03D) for 2 hours, subsequently 1% flowthrough (FT) was collected. Beads were washed three times in IP-Buffer (50mM Tris-HCl pH 8.0, 150mM NaCl, 1.5mM MgCl2, 10% glycerol, 0.1% Triton X-100 freshly added protease and phosphatase inhibitors) and finally immunoprecipitated proteins (IP) were eluted by boiling beads 10 min. in 2x Laemmli buffer. Input, IP and FT were run on 5% SDS-PAGE.

Antibodies

The anti-mouse Rif1 antibody #1240 has been described in (Buonomo et al., 2009); anti-Smc1 (Bethyl, #A300-055A) 1:10000; anti-phospho Ser345 Chk1 (Cell Signaling

Technology#2348) 1:1000; anti-Chk1 G4 (Santa Cruz #sc-8408) 1:400; anti-Oct3/4 C10 (Santa Cruz #sc5279) 1:200; anti-Nanog (Abcam ab80892) 1:1000; anti-p21 (Santa Cruz #6246) 1:200; anti-HA (Covance monoclonal HA.11 clone 16B12 #MMS-101R) 1:1000 for western blotting, 1:3000 for immunofluorescence; anti-HA ChIP and immunoprecipitation (Roche 11867423001); Anti-Flag ChIP and immunoprecipitation (Biozol 637302); rat IgG (Santa Cruz 2026); anti-lamin B1 (Abcam #ab16048) 1:1000 for both western blotting and immunofluorescence; anti β tubulin (Cell Signaling #2146) 1:1000.

3D FISH and analysis

3D FISH protocol is based on (Solovei et al., 2002) with the following changes. Slides were equilibrated in 20% glycerol in PBS for 1 hour and subsequently frozen in liquid nitrogen and thawed at room temperature. This step was repeated three times. For long-term storage, slides were kept in 70% ethanol at -20°C. Before use, slides where treated with 0.1mg/ml RNase A in 2x SSC for 1 hour at 37°C. BAC probes RP23-355H20 and RP23-209P21 (Source Bioscience) were labeled for 50 min. using Roche Nick translation kits (DIG 11745816910 and Biotin 11745824910), precipitated in a ratio 1:1 and resuspended in 70% formammide (Sigma F9037), 2x SSC and 10% dextran sulfate. Denaturation was performed for 8 min. at 85°C and hybridization was kept overnight. Post-hybridization washes were done with 0.1x SSC pre-warmed at 61°C. Blocking was carried out for 30 min. with 3% BSA, 4x SSC, 0.1% Tween 20. Anti-Dig-FITCH (Roche 11207741-910) and Strepavidin-Alexa546 (Invitrogen S-11225) were incubated for 1 hour at 37°C at a dilution 1:200 in 1% BSA, 4x SSC and 0.1% Tween 20. Acquisition was performed on a Confocal Leica TCS SP5 using a 63X oil immersion objective with

an extra optical magnification of 4x and optical sections separated by 0.25 μm. Cells with signals from both alleles were analyzed by Imaris Bitplane. During the statistical analysis of the measurements of the distance between the two FISH signals, values exceeding 2.5 the standard deviations were excluded.

Supplemental references

Bryja, V., Bonilla, S., and Arenas, E. (2006). Derivation of mouse embryonic stem cells. Nat Protoc *1*, 2082-2087.

Bulut-Karslioglu, A., Perrera, V., Scaranaro, M., de la Rosa-Velazquez, I.A., van de Nobelen, S., Shukeir, N., Popow, J., Gerle, B., Opravil, S., Pagani, M.*, et al.* (2012). A transcription factor-based mechanism for mouse heterochromatin formation. Nature structural & molecular biology *19*, 1023-1030.

Buonomo, S.B., Wu, Y., Ferguson, D., and de Lange, T. (2009). Mammalian Rif1 contributes to replication stress survival and homology-directed repair. The Journal of cell biology *187*, 385-398.

Carpenter, A.E., Jones, T.R., Lamprecht, M.R., Clarke, C., Kang, I.H., Friman, O., Guertin, D.A., Chang, J.H., Lindquist, R.A., Moffat, J.*, et al.* (2006). CellProfiler: image analysis software for identifying and quantifying cell phenotypes. Genome Biol *7*, R100.

Cayrou, C., Coulombe, P., Puy, A., Rialle, S., Kaplan, N., Segal, E., and Mechali, M. (2012). New insights into replication origin characteristics in metazoans. Cell cycle (Georgetown, Tex *11*, 658-667.

Cayrou, C., Coulombe, P., Vigneron, A., Stanojcic, S., Ganier, O., Peiffer, I., Rivals, E., Puy, A., Laurent-Chabalier, S., Desprat, R.*, et al.* (2011). Genome-scale analysis of metazoan replication origins reveals their organization in specific but flexible sites defined by conserved features. Genome Res *21*, 1438-1449.

Cornacchia, D., Dileep, V., Quivy, J.P., Foti, R., Tili, F., Santarella-Mellwig, R., Antony, C., Almouzni, G., Gilbert, D.M., and Buonomo, S.B. (2012). Mouse Rif1 is a key

regulator of the replication-timing programme in mammalian cells. The EMBO journal *31*, 3678-3690.

Dan, J., Liu, Y., Liu, N., Chiourea, M., Okuka, M., Wu, T., Ye, X., Mou, C., Wang, L., Wang, L., *et al*. (2014). Rif1 Maintains Telomere Length Homeostasis of ESCs by Mediating Heterochromatin Silencing. Dev Cell *29*, 7-19.

Dixon, J.R., Selvaraj, S., Yue, F., Kim, A., Li, Y., Shen, Y., Hu, M., Liu, J.S., and Ren, B. (2012). Topological domains in mammalian genomes identified by analysis of chromatin interactions. Nature *485*, 376-380.

Gao, F., Luo, H., and Zhang, C.T. (2012). DeOri: a database of eukaryotic DNA replication origins. Bioinformatics (Oxford, England) *28*, 1551-1552.

Hagege, H., Klous, P., Braem, C., Splinter, E., Dekker, J., Cathala, G., de Laat, W., and Forne, T. (2007). Quantitative analysis of chromosome conformation capture assays (3C-qPCR). Nat Protoc *2*, 1722-1733.

https://pypi.python.org/pypi/MACS2.

Langmead, B., and Salzberg, S.L. (2012). Fast gapped-read alignment with Bowtie 2. Nat Methods *9*, 357-359.

Lehnertz, B., Ueda, Y., Derijck, A.A., Braunschweig, U., Perez-Burgos, L., Kubicek, S., Chen, T., Li, E., Jenuwein, T., and Peters, A.H. (2003). Suv39h-mediated histone H3 lysine 9 methylation directs DNA methylation to major satellite repeats at pericentric heterochromatin. Curr Biol *13*, 1192-1200.

Lund, E., Oldenburg, A.R., and Collas, P. (2014). Enriched domain detector: a program for detection of wide genomic enrichment domains robust against local variations. Nucleic acids research *42*, e92.

Meuleman, W., Peric-Hupkes, D., Kind, J., Beaudry, J.B., Pagie, L., Kellis, M., Reinders, M., Wessels, L., and van Steensel, B. (2013). Constitutive nuclear lamina-genome interactions are highly conserved and associated with A/T-rich sequence. Genome Res *23*, 270-280.

Peric-Hupkes, D., Meuleman, W., Pagie, L., Bruggeman, S.W., Solovei, I., Brugman, W., Graf, S., Flicek, P., Kerkhoven, R.M., van Lohuizen, M.*, et al.* (2010). Molecular maps of the reorganization of genome-nuclear lamina interactions during differentiation. Molecular cell *38*, 603-613.

Ramirez, F., Dundar, F., Diehl, S., Gruning, B.A., and Manke, T. (2014). deepTools: a flexible platform for exploring deep-sequencing data. Nucleic acids research.

Ryba, T., Battaglia, D., Pope, B.D., Hiratani, I., and Gilbert, D.M. (2011). Genome-scale analysis of replication timing: from bench to bioinformatics. Nat Protoc *6*, 870-895.

Smyth, G.K. (2004). Linear models and empirical bayes methods for assessing differential expression in microarray experiments. Stat Appl Genet Mol Biol *3*, Article3.

Solovei, I., Cavallo, A., Schermelleh, L., Jaunin, F., Scasselati, C., Cmarko, D., Cremer, C., Fakan, S., and Cremer, T. (2002). Spatial preservation of nuclear chromatin architecture during three-dimensional fluorescence in situ hybridization (3D-FISH). Experimental cell research *276*, 10-23.

Splinter, E., de Wit, E., van de Werken, H.J., Klous, P., and de Laat, W. (2012). Determining long-range chromatin interactions for selected genomic sites using 4C-seq technology: from fixation to computation. Methods *58*, 221-230.

Stadhouders, R., Kolovos, P., Brouwer, R., Zuin, J., van den Heuvel, A., Kockx, C., Palstra, R.J., Wendt, K.S., Grosveld, F., van Ijcken, W.*, et al.* (2013). Multiplexed

chromosome conformation capture sequencing for rapid genome-scale high-resolution detection of long-range chromatin interactions. Nat Protoc *8*, 509-524.

Takebayashi, S., Dileep, V., Ryba, T., Dennis, J.H., and Gilbert, D.M. (2012). Chromatin-interaction compartment switch at developmentally regulated chromosomal domains reveals an unusual principle of chromatin folding. Proceedings of the National Academy of Sciences of the United States of America *109*, 12574-12579.

Thongjuea, S., Stadhouders, R., Grosveld, F.G., Soler, E., and Lenhard, B. (2013). r3Cseq: an R/Bioconductor package for the discovery of long-range genomic interactions from chromosome conformation capture and next-generation sequencing data. Nucleic acids research *41*, e132.
