# Supplementary material for: Nuclear Architecture Organized by Rif1 Underpins the Replication-Timing Program
Source: Mol Cell. 2016 Jan 21;61(2):260–73. doi: 10.1016/j.molcel.2015.12.001 (PMC4724237; doi:10.1016/j.molcel.2015.12.001)
Supplement: Document S2. Article plus Supplemental Information [file mmc2.pdf]

Article

Molecular Cell

Nuclear Architecture Organized by Rif1 Underpins the Replication-Timing Program

Graphical Abstract

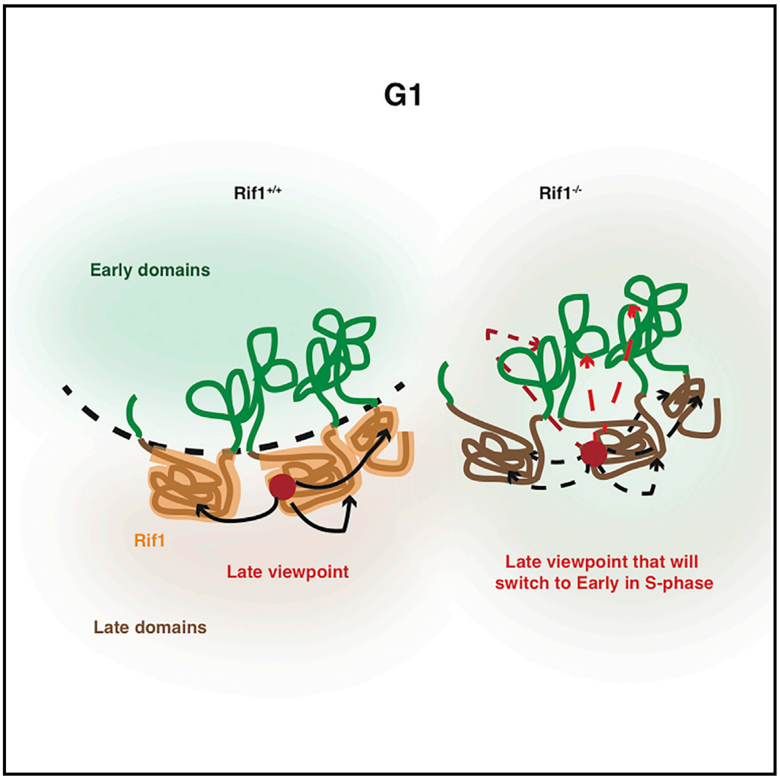

Authors

Rossana Foti, Stefano Gnan, Daniela Cornacchia, ..., Thomas Manke, Thomas Jenuwein, Sara C.B. Buonomo

Correspondence

sara.buonomo@ed.ac.uk

In Brief

At replication-timing establishment, chromatin domains are forced to interact only with regions sharing the same replication timing. Foti et al. demonstrate that Rif1 is responsible for this constraint because Rif1 deletion leads to loss of spatial limitations followed by replication-timing program disruption. Therefore, Rif1 links nuclear architecture and replication-timing establishment.

Highlights

- Most late-replicating regions are marked by Rif1 (RADs)
- Late replication in RADs is differentially regulated where Lamin B1 is stably bound
- Rif1 constrains inter-domain contacts within the same replication timing in G1
- Rif1 coordinates the replication timing of interacting domains

Foti et al., 2016, Molecular Cell *61*, 260–273
January 21, 2016 ©2016 The Authors
http://dx.doi.org/10.1016/j.molcel.2015.12.001

Molecular Cell
Article

Nuclear Architecture Organized by Rif1 Underpins the Replication-Timing Program

Rossana Foti,[1] Stefano Gnan,[1] Daniela Cornacchia,[1,8] Vishnu Dileep,[2] Aydan Bulut-Karslioglu,[3,9] Sarah Diehl,[3] Andreas Buness,[1] Felix A. Klein,[7] Wolfgang Huber,[7] Ewan Johnstone,[4] Remco Loos,[4] Paul Bertone,[4,5,6] David M. Gilbert,[2] Thomas Manke,[3] Thomas Jenuwein,[3] and Sara C.B. Buonomo[1,10,*]
[1]Mouse Biology Unit, EMBL Monterotondo, Via Ramarini 32, 00015 Monterotondo, Italy
[2]Department of Biological Science, Florida State University, Tallahassee, FL 32306, USA
[3]Max Planck Institute of Immunbiology and Epigenetics, Stubeweg 51, 79108 Freiburg, Germany
[4]European Molecular Biology Laboratory, European Bioinformatics Institute, Wellcome Trust Genome Campus, Cambridge CB10 1SD, UK
[5]Genome Biology and Developmental Biology Units, European Molecular Biology Laboratory, Meyerhofstrasse 1, 69117 Heidelberg, Germany
[6]Wellcome Trust–Medical Research Council Stem Cell Institute, University of Cambridge, Tennis Court Road, Cambridge CB2 1QR, UK
[7]Genome Biology Unit, European Molecular Biology Laboratory, Meyerhofstrasse 1, 69117 Heidelberg, Germany
[8]Present address: Lorenz Studer Group, Center for Stem Cell Biology, Memorial Sloan Kettering Cancer Center, Rockefeller Research Laboratories, 430 East 67th Street, New York, NY 10065, USA
[9]Present address: Eli and Edythe Broad Center of Regeneration Medicine and Stem Cell Research, Department of Obstetrics and Gynecology and Center for Reproductive Sciences, University of California, San Francisco, 35 Medical Center Way, San Francisco, CA 94143, USA
[10]Present address: School of Biological Sciences, University of Edinburgh, Roger Land Building, Alexander Crum Brown Road, Edinburgh EH9 3FF, UK
*Correspondence: sara.buonomo@ed.ac.uk
http://dx.doi.org/10.1016/j.molcel.2015.12.001
This is an open access article under the CC BY license (http://creativecommons.org/licenses/by/4.0/).

SUMMARY

DNA replication is temporally and spatially organized in all eukaryotes, yet the molecular control and biological function of the replication-timing program are unclear. Rif1 is required for normal genome-wide regulation of replication timing, but its molecular function is poorly understood. Here we show that in mouse embryonic stem cells, Rif1 coats late-replicating domains and, with Lamin B1, identifies most of the late-replicating genome. Rif1 is an essential determinant of replication timing of non-Lamin B1-bound late domains. We further demonstrate that Rif1 defines and restricts the interactions between replication-timing domains during the G1 phase, thereby revealing a function of Rif1 as organizer of nuclear architecture. Rif1 loss affects both number and replication-timing specificity of the interactions between replication-timing domains. In addition, during the S phase, Rif1 ensures that replication of interacting domains is temporally coordinated. In summary, our study identifies Rif1 as the molecular link between nuclear architecture and replication-timing establishment in mammals.

INTRODUCTION

The eukaryotic genome is organized into domains whose replication follows a cell-type distinctive temporal order that is defined when the associated replication origins are activated during the S phase (reviewed in Rhind and Gilbert, 2013). In yeast, several origin-binding DNA replication factors are available in limiting amounts. Their interaction either promotes or antagonizes the activation of the loaded helicases at each origin and determines the probability, and thus the order, of firing (Mantiero et al., 2011; Patel et al., 2006; Tanaka et al., 2011; Wu and Nurse, 2009). These findings demonstrate that the execution of the DNA replication-timing (RT) program is controlled at the level of individual origins during the S phase. In contrast, the establishment of the RT program is arranged in mammalian cells in the early G1 phase before the specification of the origins, during the timing decision point (TDP) (Dimitrova and Gilbert, 1999). The TDP coincides with the completion of three-dimensional (3D) chromatin reorganization, after mitosis, suggesting a role for higher-order chromatin organization in defining the temporal DNA replication program (Dileep et al., 2015). Genome-wide analysis of DNA replication domain distribution highlights a striking coincidence with the 3D organization of the chromatin domains (Pope et al., 2014; Ryba et al., 2010; Yaffe et al., 2010). For instance, replication domains precisely overlap with topologically associating domains (TADs), chromatin units defined by high a frequency of interactions, which provides a method of replication domain identification independent of RT (Pope et al., 2014). In addition, changes in RT generally coincide with spatial re-localization of genomic loci relative to the nuclear periphery (Hiratani et al., 2008) and re-organization of chromatin contacts with neighboring loci, allowing maintenance of preferential interactions between domains displaying the same RT (Takebayashi et al., 2012). The establishment of the RT program is therefore independent of the individual

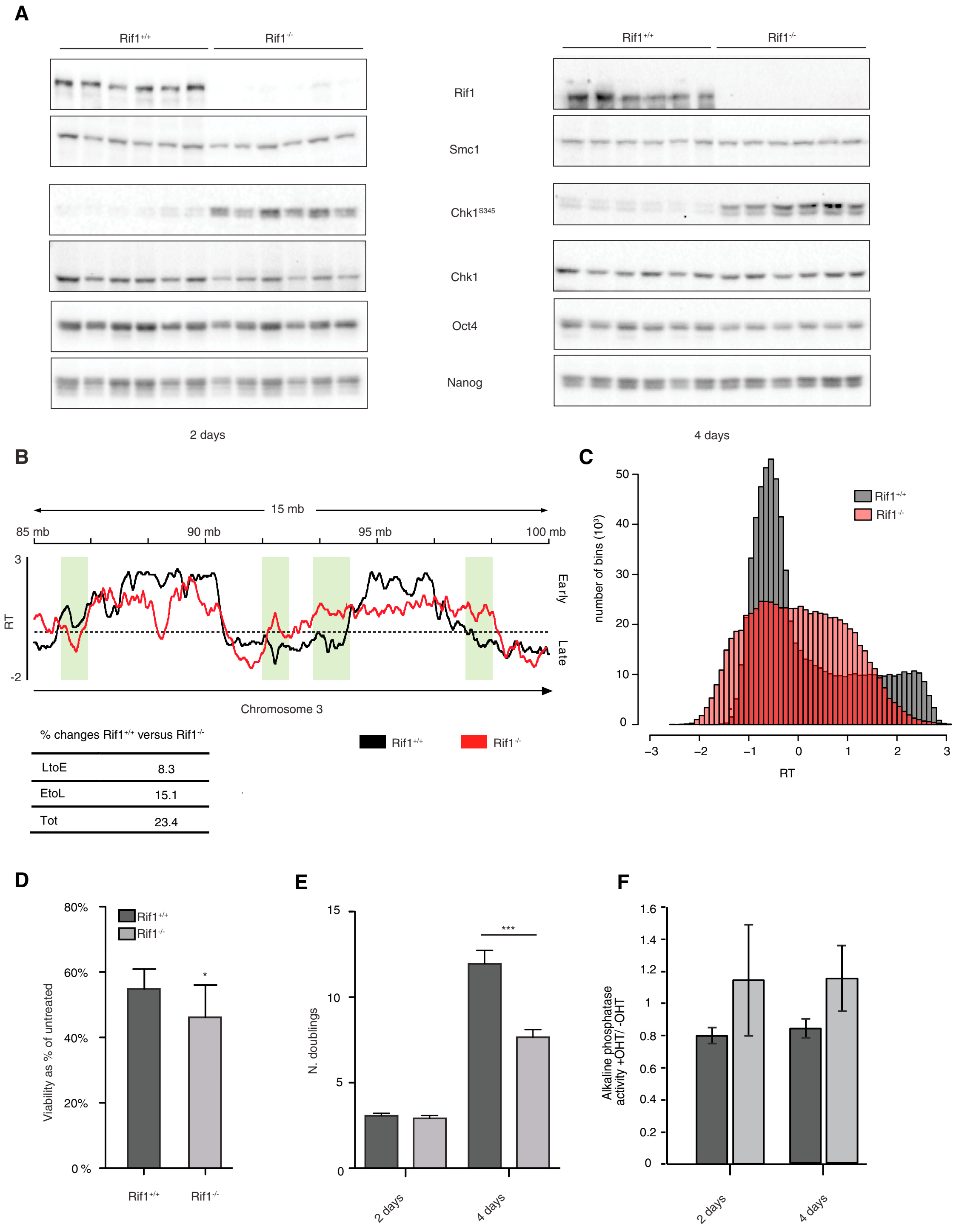

Figure 1. Cell Cycle Responses to Rif1 Deficiency in ESCs

(A) Western blot analysis of the Rif1 deletion time course in six independent Rif1$^{-/-}$ and Rif1$^{+/+}$ cells lines. Left and right panels show 2 and 4 days, respectively, after Cre induction. Smc1 is the loading control.

(B) Loess smoothed representative RT profiles averaged from two Rif1$^{+/+}$ and four Rif1$^{-/-}$ ESC lines. RT = $\log_2$(early/late). Regions showing RT switches are shadowed in green. The table summarizes the percentage of RT changes.

(C) Using tiles of 60 bp, the genome-wide distribution of the RT scores is shown for averages of two Rif1$^{+/+}$ and four Rif1$^{-/-}$ lines in ESCs.

(D) The 3-(4,5-dimethylthiazol-2-yl)-2,5-diphenyltetrazolium bromide (viability) assay 4 days after Cre induction. Shown are the averages from triplicates of six independent Rif1$^{+/+}$ versus Rif1$^{-/-}$ ESCs from three experiments. Error bars indicate SDs, and p values were calculated by t test.

(legend continued on next page)

origins and may be linked to the spatial organization of the chromatin in the nucleus.

Little is known about the molecular components involved in the establishment of the RT program. In budding yeast, Fhk1/2 influence genome-wide RT by controlling replication origin clustering (Knott et al., 2012). Taz1 in fission yeast counteracts the activation of about half of the late chromosomal origins (Tazumi et al., 2012), while in human cells, polymerase Θ is involved in RT by a yet-unknown mechanism (Fernandez-Vidal et al., 2014). We and others have recently shown that Rif1 is a genome-wide regulator of RT across evolution (Cornacchia et al., 2012; Davé et al., 2014; Hayano et al., 2012; Hiraga et al., 2014; Lian et al., 2011; Mattarocci et al., 2014; Peace et al., 2014; Yamazaki et al., 2012). Rif1 was originally discovered in budding yeast as a negative regulator of telomere length (Hardy et al., 1992), although this role is not conserved in mammals (Buonomo et al., 2009). The telomere-length and RT regulatory functions of *S. cerevisiae* Rif1 are likely connected. Suddenly shortening the telomere induces switching of their late RT to the early S phase (Bianchi and Shore, 2007). Recently, Rif1 was found to cooperate with the protein phosphatase 1 (PP1) to control RT in budding and fission yeast by counteracting origin activation by Dbf4-dependent kinase (Davé et al., 2014; Hiraga et al., 2014; Mattarocci et al., 2014). However, the exact mechanism of this function is still unclear, because Rif1 could not be detected at the origins of replication.

Because RT can be envisaged as a two-stage program, the genome-wide alterations that we observed following Rif1 knockout in fibroblasts (Cornacchia et al., 2012) could reflect defective establishment, an execution step, or both. In this study, we set out to elucidate at what stage and how Rif1 controls the RT program. Because the establishment takes place over large chromosomal regions while the execution occurs at the level of individual origins, analyzing Rif1 genome-wide distribution could provide an important indication of the stage at which it performs its function. We show that Rif1 coats the late-replicating genome, forming large Rif1-associated domains (RADs) with a substantial degree of overlap with Lamin B1-associated domains (LADs). Much evidence associates Rif1 and the lamina (this work; Cornacchia et al., 2012; Yamazaki et al., 2013), a structure with a well-documented role in the organization of chromatin architecture (reviewed in Shimi et al., 2010). We hypothesized that Rif1 could be an organizer of nuclear architecture, linking the establishment of RT with chromatin organization within the nuclear volume. In this study, we provide evidence that Rif1 coordinates inter-domain interactions before S phase and that its loss results first in disorganization of inter-domain chromatin contacts, followed by loss of a stable and reproducible RT program.

RESULTS

Rif1 Controls RT across Different Cell Types

Mouse embryonic stem cells (ESCs) are an ideal model system for the genome-wide study of RT control because of the high percentage of S-phase cells that permit isolation of sufficient material to study the mechanism of Rif1 function and the possible role in nuclear architecture organization. Cre-mediated deletion of Rif1 in ESCs (Figure 1A) induces genome-wide, bidirectional RT switches (Figure 1B), with loss of the typical early/late bi-modal distribution (Figure 1C). The unimodal distribution of genome-wide RT in Rif1$^{-/-}$ cells, centered at zero, suggests loss of temporal resolution of origin firing or, more extremely, that most genomic positions analyzed have the same probability of replicating in the early or late fraction. These data suggest the loss of a stable and reproducible program. In addition, as in primary mouse embryonic fibroblasts (pMEFs) (Cornacchia et al., 2012), deletion of Rif1 induces high levels of fragmentation of the larger replication domains (Figure S1A).

We have previously shown that Rif1 deletion in pMEFs induces the G1/S checkpoint (Cornacchia et al., 2012) and arrests proliferation (Buonomo et al., 2009) because of checkpoint activation (Figure S1B). In contrast, in ESCs, Rif1 deficiency activates the DNA replication checkpoint response (phosphorylation of Chk1 on Ser345; Figure 1A; Figure S1C), decreasing cell viability (Figure 1D) but not arresting proliferation (Figure 1E; Figure S1D).

Because chronic exposure to DNA damage induces differentiation of ESCs (Lin et al., 2005; Qin et al., 2007), we assayed the pluripotency state of Rif1$^{+/+}$ and Rif1$^{-/-}$ ESCs at various stages after Rif1 deletion. We did not detect significant alterations in the levels of expression of the pluripotency markers Oct4 and Nanog (Figure 1A) or in alkaline phosphatase activity (Figure 1F) within the time frame of our experiment (Figures S1E–S1G). In summary, our data indicate that the core function of Rif1 in RT regulation is conserved across different cell types (i.e., ESCs and MEFs), although the cell-type-specific response to its deletion can vary depending on the checkpoint activated.

Rif1-Bound Domains Identify the Late-Replicating Genome

Having validated that the core function of Rif1 is conserved in ESCs, we analyzed its genome-wide occupancy. Rif1's distribution overlaps extensively with late-replicating regions, while it is generally depleted from early replicating domains (Figures 2A and 2B; Figures S2A and S2B). Because late-replicating regions associate with the nuclear lamina (Hansen et al., 2010; Peric-Hupkes et al., 2010), RADs largely correspond to genomic regions associated with the nuclear lamina (LADs) (Figure 2A) (Peric-Hupkes et al., 2010) and, more specifically, overlap with LADs that are invariant among cell types (cLADs) (Figure S2B) (Meuleman et al., 2013). We confirmed the association of Rif1

(E) Cell proliferation measured as the averages from triplicates of six independent Rif1$^{+/+}$ versus Rif1$^{-/-}$ ESCs from three experiments (paired t test, ***$p < 0.0001$). Error bars indicate SDs.

(F) Results 2 and 4 days after Cre-induction cells were assayed for alkaline phosphatase activity. +OHT, Rif1$^{+/+}$ or Rif1$^{F/F}$ ESCs treated with 4-hydroxytamoxifen for the indicated duration; −OHT, untreated cells. The average of two biological replicas assayed in triplicates is shown. The error bars indicate SDs. The t test reveals no significant difference.

See also Figure S1.

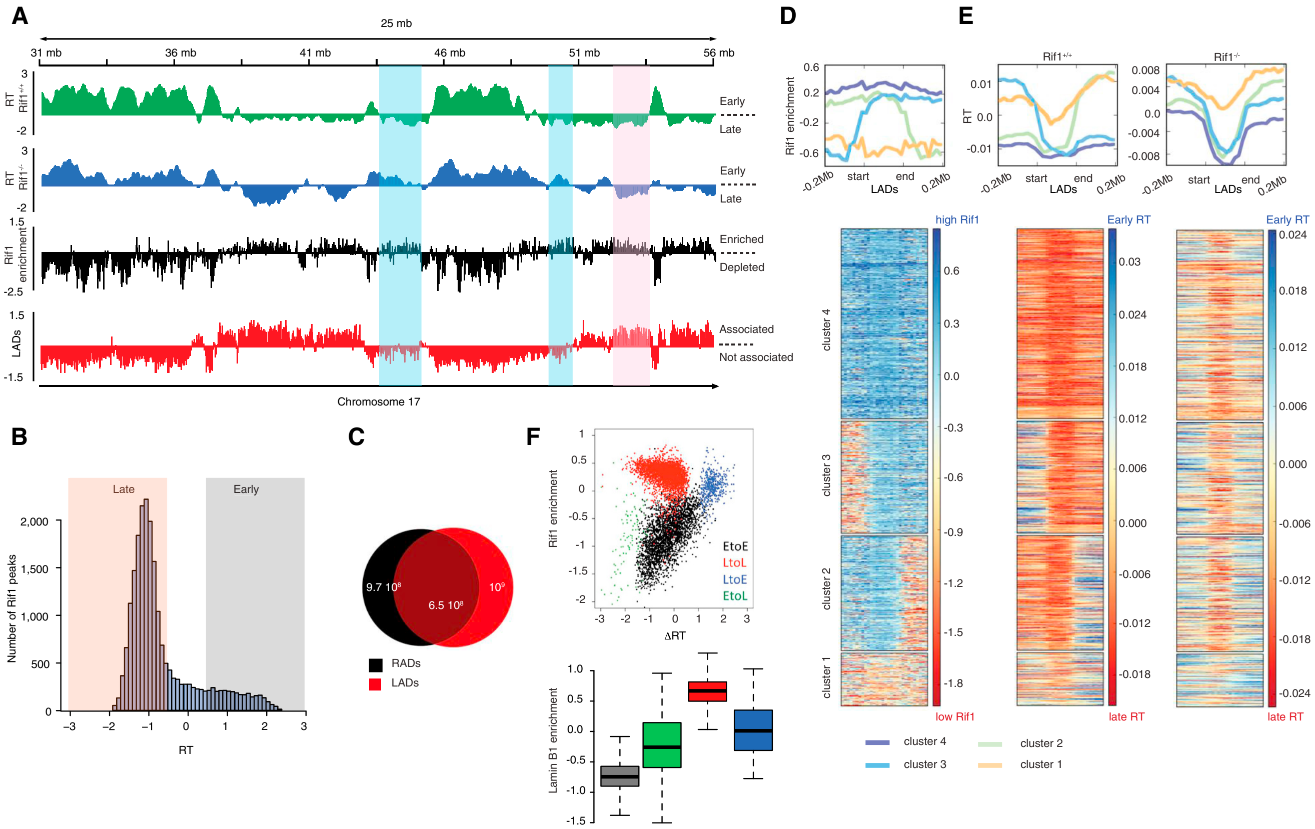

Figure 2. ChIP-Seq Analysis of Genome-wide Rif1 Occupancy in ESCs

(A) Representative profile from chromosome 17, comparing RT (RT = $\log_2$(early/late)) averaged from two Rif1$^{+/+}$ and four Rif1$^{-/-}$ ESCs, with Rif1 distribution from one representative out of three replicas (enrichment = $\log_2$(ChIP/input)) and LADs. Shadowed in blue are late-replicating RAD-LB^{-}, switching to early replication in Rif1$^{-/-}$. In red is highlighted one example of late-replicating RAD-LB^{+}, where Rif1 deletion does not affect RT.

(B) Distribution of the RT score over the Rif1 binding profile in one representative ESC line out of three. Late-replicating domains (RT $\leq$ 0.5) are shadowed pink, and early ones are in gray (RT $\geq$ 0.5).

(C) Venn diagram indicating the overlap in base pairs between LADs and RADs, as defined by the EDD algorithm. One representative out of two independent cell lines analyzed is shown.

(D) Meta-analysis of Rif1 distribution over LADs. Flanking regions of ±0.2 Mb (non-LADs) were included around the start and end of each LAD. ChIP-seq data were obtained and analyzed from three independent ESC lines. The results presented are from one representative line. The heatmap shows four classes of LADs that were obtained from unsupervised clustering of the Rif1 data and correspond to the different distributions of Rif1 around the LAD boundaries.

(E) Replication status of LADs is shown for Rif1$^{+/+}$ and Rif1$^{-/-}$ ESCs. The LADs are ordered in the same way as the cluster solution of Rif1 enrichment in (D).

(F) Scatter plot showing Rif1 enrichment relative to the RT changes (ΔRT = Rif1$^{-/-}$ – Rif1$^{+/+}$) and, boxplot showing Lamin B1 association for regions switching (EtoL and LtoE) or not switching (EtoE and LtoL) their RT upon Rif1 deletion.

See also Figure S2.

and the nuclear lamina by immunofluorescence, where Rif1 is enriched at the nuclear periphery in proximity with Lamin B1 (Figure S2C), and by co-immunoprecipitation of Rif1 and Lamin B1 (Figure S2D). The overlap between RADs and LADs is extensive (Figure 2C). Together, RADs and LADs constitute 73% of all late-replicating regions (Figure S2E), distinguishing two types of late-replicating domains: those that are bound concomitantly by Rif1 and Lamin B1 (RAD-LB^{+}) and those that are mostly bound by Rif1 alone (RAD-LB^{-}). In contrast, domains marked by Lamin B1 alone tend to have a less defined timing of replication and therefore cannot be strictly classified as late domains (cluster 1 in Figures 2D and 2E). Among the RAD-LB^{+}, late RT is independent of Rif1, or is controlled either redundantly or independently of both Rif1 and Lamin B1, because these regions maintain their RT in Rif1 null cells (Figures 2A, 2D, and 2E; late to late [LtoL] in Figure 2F; Figures S2F and S2G). By contrast, RAD-LB^{-} constitute most of the late-replicating regions that switch to early replication in Rif1 null cells (Figures 2A, 2D, and 2E; late to early [LtoE] in Figure 2F; Figures S2F and S2G). Finally we hypothesize that the RT changes that occur within the Rif1-devoided early domains in response to Rif1 deletion (Figure 2A; early to late [EtoL] in Figure 2F) are indirect consequences of the increased competition for limiting S-phase promoting factors due to the earlier replication of RAD-LB^{-} domains, in agreement with what was recently reported for yeast Sir2 (Yoshida et al., 2014). In summary, our data show that Rif1 resides on large domains, remarkably coinciding with late-replicating genomic regions and LADs. This type of distribution suggests a possible

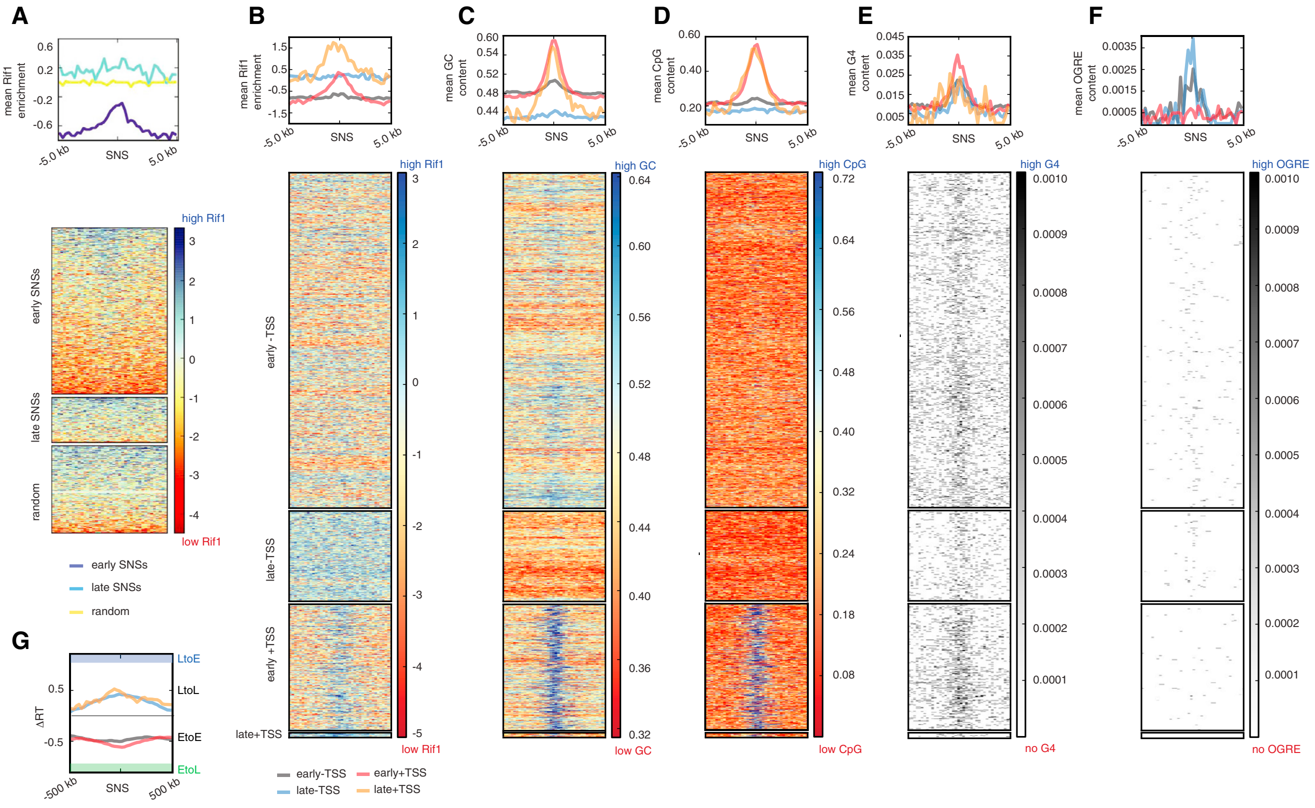

Figure 3. Rif1-Bound Early SNSs Are Not Enriched in EtoL Regions

(A) Representative heatmap showing the distribution of the Rif1 signal around SNSs on chromosome 11 for one out of three ESC lines analyzed. For comparison, a random set of loci was chosen from the same region of chromosome 11.

(B–F) Analysis of Rif1 enrichment, GC and CpG content, and G4 and OGRE association of early and late SNSs stratified by their overlap with TSSs for one representative out of three ESC lines analyzed. The order of SNSs is identical for all heatmaps. The SNSs were classified as early or late depending on the average score of their replication domains (late: RT < −0.5, early: RT > 0.5, 200 kb bins).

(B) Rif1 enrichment (enrichment = $\log_2$(ChIP/input)) ±5 kb at the center of SNSs. Rif1 presence depends on SNS association with TSSs in early domains and for a very small number of late-replicating, overlapping SNSs and TSSs.

(C and D) Analysis of SNSs' GC and CpG content. TSS-associated SNSs (same cluster displaying Rif1 enrichment) feature high CG (C) and CpG (D) content. Mean CpG content = $CpG/(GC/2)^2$.

(E) G4 motif instances are plotted as black lines in a discretized matrix at the center of SNSs. The heatmap and average profile reveal that SNSs show a prevalence of G4 motifs in all groups, independent of their replication status or overlap with TSSs.

(F) As in (E), but for the less abundant OGRE motif. There is no clear association with SNSs, but there is on average a slight preference for origins without TSSs.

(G) Changes of RT ($\Delta RT = Rif1^{-/-} - Rif1^{+/+}$) within 500 kb at the center of SNSs. Regions that upon Rif1 deletion change their RT of more than ±1 (LtoE and EtoL; $\Delta RT > +1$ and $\Delta RT < -1$) are considered switching while the others ($-1 < \Delta RT < +1$) are not (EtoE and LtoL). TSS-associated SNSs (same cluster displaying Rif1 enrichment) have the same ΔRT as SNSs that are not associated with TSSs.

role for Rif1 in the 3D organization of the mouse late-replicating genome.

A Small Fraction of Rif1 Is Focally Enriched at CpG-Rich TSSs

While most Rif1 is found in RADs, a fraction of Rif1 is distributed in the form of sharp enrichments in both early and late-replicating regions, as determined by MACS (see Supplemental Information). The sharp peaks formed by mouse Rif1 could represent a fraction of the protein specifically bound to the origins of replication. We therefore compared Rif1 binding to the 2,405 potential DNA replication origins (small nascent strands [SNSs]) mapped on a section of chromosome 11 (Cayrou et al., 2011). Rif1 sharp peaks are associated with 303 (12.6%) SNSs, showing a focal increase with respect to a depleted background in early domains and a broad enrichment in late domains (Figure 3A). This is distinct from fission yeast, where although not bound to, Rif1 tends to be closer to late origins (Hayano et al., 2012). To inspect the association of Rif1 with mouse origins more thoroughly, we integrated Rif1 sharp signals with additional genomic features that have been associated with mammalian origins of replications, such as transcription start sites (TSSs) (Cadoret et al., 2008; Sequeira-Mendes et al., 2009), GC/CpG content, and sequence motifs, such as G quadruplexes (G4s) (Besnard et al., 2012) and the origin G-rich repeated elements (OGREs) (Cayrou et al., 2012). Rif1 is enriched in correspondence with TSS-associated SNSs (Figure 3B), GC rich (Figure 3C), and CpG rich (Figure 3D). The presence of G4s (Figure 3E) or OGREs

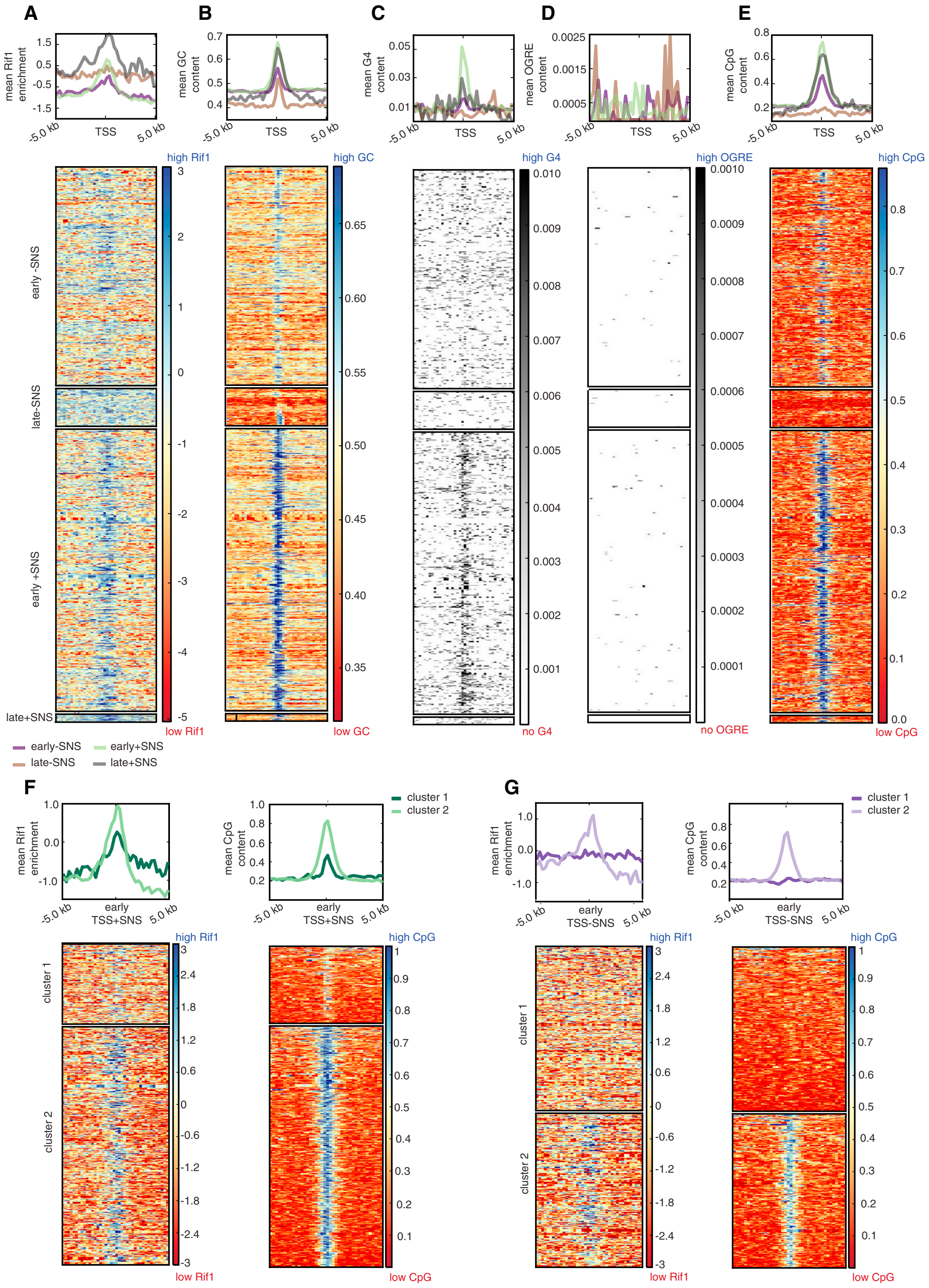

Figure 4. Rif1 Is Associated with CpG-Rich TSSs

(A–E) Rif1-bound TSSs in the region of chromosome 11, where SNSs have been mapped, were subdivided based on their overlap with SNSs or lack thereof. Data from one representative ESC line out of three analyzed are shown.

(A) The mean Rif1's enrichment at early TSSs is independent of their association with SNSs. However, SNS presence contributes to better enrichment.

(legend continued on next page)

(Figure 3F) seems instead to be only generically related with presence of SNSs (the former) or TSS-free SNSs (the latter). To investigate whether the preference of Rif1 for TSS-associated, GC/CpG-rich origins is due to a general tendency of Rif1 to bind TSSs, we also performed the inverse analysis. We considered Rif1 distribution around the TSSs on the region of chromosome 11 where SNSs were mapped and stratified them depending on their replication status and association with SNSs. We found that early TSSs in general and late TSSs overlapping with SNSs (a very small number) show an increase of Rif1 (Figure 4A). Rif1's enrichment is correlated with the GC content (Figure 4B) and, to a lesser extent, with the presence of G4 motifs (Figure 4C), while the presence of OGRE motifs does not show any correspondence (Figure 4D). Most importantly, CpG content seems to be a good predictor of Rif1 enrichment, especially in early replicating regions (Figure 4E). More detailed analysis of the correlation between Rif1 enrichment levels and CpG content revealed that, irrespective of the association with SNSs, CpG-rich TSSs are enriched for Rif1 (Figures 4F and 4G). These are often highly transcribed regions more prone to ChIP artifacts (Teytelman et al., 2013). It would therefore be important to associate Rif1 presence on this small and specific subset of origins with a function. However, Rif1 binding to these SNSs before Cre induction bears no consequences for the RT changes induced by Rif1 deletion in the region. The probability to switch to late replication in Rif1 null cells for SNSs associated or not to TSSs (and Rif1) is indistinguishable (Figure 3G).

In summary, most base pairs covered by Rif1 is in the shape of late domains (Figures 2A and 2B) that are mostly depleted of mapped SNSs. The bulk of mapped origins resides in early domains and is depleted of Rif1, except for a small subset of CpG-rich TSSs-associated SNSs. However, this subgroup does not show any specific response to Rif1 deletion, and Rif1 peaks in early domains do not fit the functional model drawn based on the yeast data. Because our analysis was limited to a section of chromosome 11, we cannot formally exclude that Rif1 could bind to a specific subclass of replication origins. However, overall, our data argue against the idea that Rif1's control of RT is exerted primarily at the level of individual origins.

Long-Term Proliferation in Absence of Rif1 Leads to Gene Expression Changes

Rif1 association to a large set of TSSs (Figure S3A; Table S1) could alternatively hint at a function during gene expression regulation. We have therefore analyzed the effect of Rif1 loss on an ESC's gene expression profile, revealing a progressive mild deregulation (Figures S3B and S3C). However, our analysis could not identify any specific link between Rif1 distribution and deregulated genes or their function (Figures S3D and S3E). Because Rif1 deletion in pMEFs does not affect gene expression (Cornacchia et al., 2012), these data suggest that Rif1 is not directly essential for the control of gene expression.

The explanation for such a mild and generic effect on gene expression (and possibly on RT) could be that Rif1 deficiency could induce epigenetic alterations that, in turn, would independently affect both processes. We have therefore analyzed the genome-wide profile of some histone modifications that have been linked to gene expression regulation (H3K4me3, H3K27me3, and H3K9me3), to heterochromatin assembly (H3K9me3 and H4K20me3), or potentially to origins activity (H3K4me3, especially for early origins; H3K9me3 for late origins [reviewed in Rivera et al., 2014]; and H4K20me3 [Beck et al., 2012]) 2 days after Rif1 deletion. As in pMEFs (Cornacchia et al., 2012), we found no effect of Rif1 deficiency on total levels of modified histones in ESCs (data not shown). In addition, their genome-wide distribution (Figures S4A–S4D) or amount localized to specific regions like TSSs (Figures S4E–S4G), SNSs (Figure S5A), or major satellites (Figures S5B and S5C) is unchanged. In summary, we have found no evidence that the effects of Rif1 deficiency could be immediately attributable to an impact on the epigenetic landscape of ESCs.

The effect of Rif1 deficiency on gene expression is apparent only in cell types that, like HeLa (Yamazaki et al., 2012) or ESCs (this work), do not respond to its deletion by p21 upregulation or cell growth arrest. Accordingly, large T antigen-mediated inhibition of the p21-mediated growth arrest in Rif1-deficient MEFs also mildly alters the transcriptome composition (Figure S5D), suggesting that changes in gene expression are secondary to proliferation. In summary, although the correlation between the presence of the few detectable Rif1 sharp peaks and that of several TSSs raises intriguing questions about the relationship between the regulation of DNA replication and transcription, we could not identify at this stage any general functional requirement for Rif1 in the transcriptional control of Rif1-bound TSSs.

Rif1 Organizes Inter-RT Domain Contacts

To test whether Rif1 could be an organizer of nuclear 3D architecture, we compared nuclear organization of replication domain interactions in Rif1$^{+/+}$ and Rif1$^{-/-}$ ESCs by circularized chromosome conformation capture sequencing (4C-seq) (Figure 5A). We chose five viewpoints and probed their genome-wide contacts. Three of them are located in regions displaying both RT and transcriptional changes upon Rif1 deletion, while the remaining two represent loci that show either only RT or only gene expression changes (Figure 5B). In each case, Rif1 deficiency significantly increased the total number of positions found in spatial proximity to the viewpoint (contacts) (Figures 5C

(B and E) GC content and CpG ratio surrounding TSSs, respectively. Both features are largely independent of RT of the TSSs and their association with SNSs (apart from the group of late TSSs without SNSs).

(C and D) Motif content for G4s and OGREs surrounding TSSs, respectively. A clear enrichment of G4s can be observed around early TSSs with SNSs, while the OGRE motif does not correlate with any of the predefined groups.

(F and G) Unsupervised clustering of the two largest TSS groups from the investigated region of chromosome 11, based on the CpG ratio.

(F) Early TSSs overlapping with SNSs form two clusters differing in their CpG content. Rif1's enrichment in each cluster is proportional to the corresponding CpG ratio.

(G) Early TSSs not associated to SNSs are clearly divided in CpG-rich and no-CpG clusters. Rif1 is enriched only at CpG-rich TSSs.

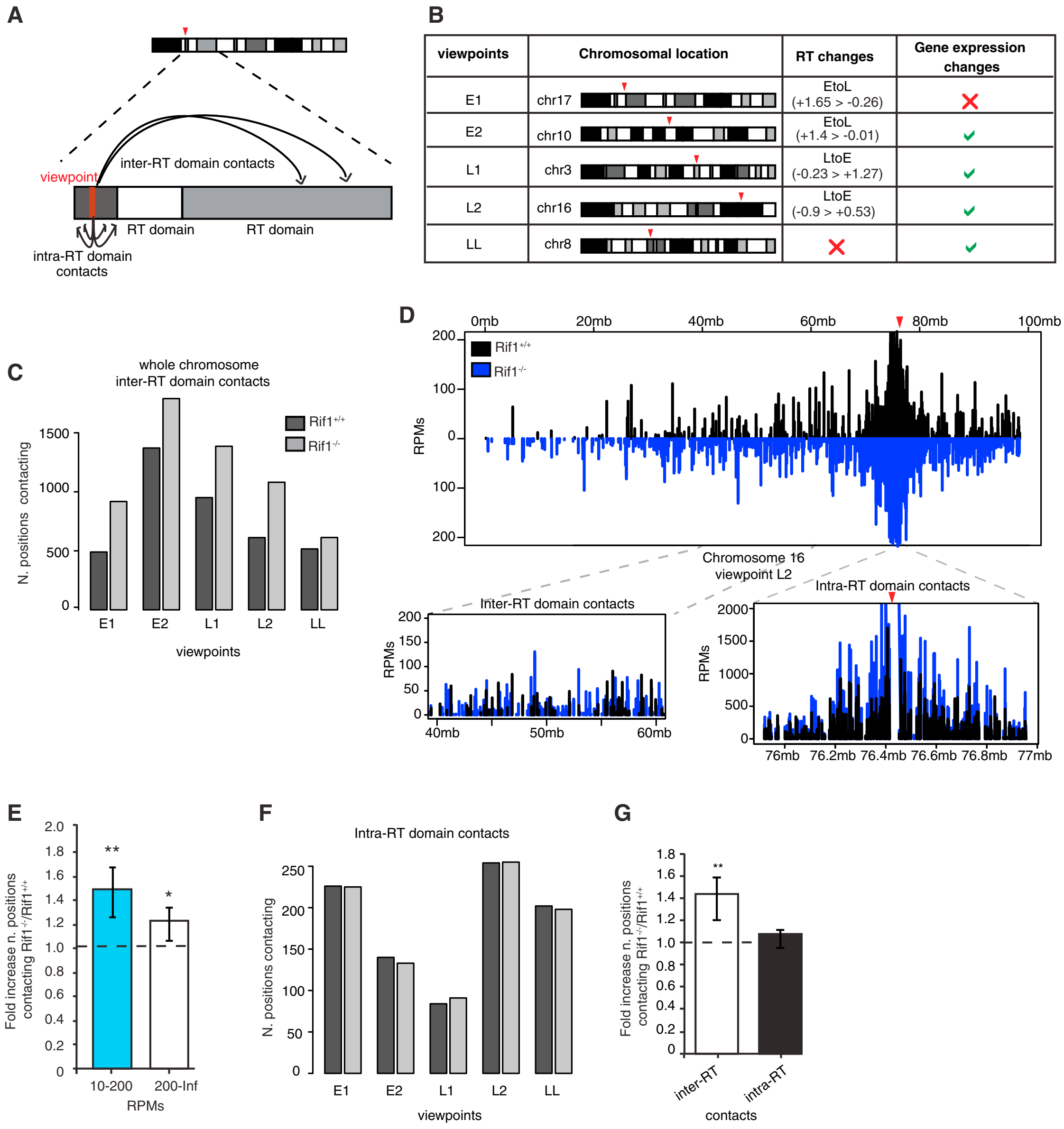

Figure 5. Rif1 Deficiency Affects Inter-RT Domain Interactions in ESCs

(A) Schematic representation of the chromatin contacts, highlighting the distinction between inter- and intra-RT domain interactions. Contacts are positions consistently identified by the r3Cseq software package analysis of 4C-seq data in the two replicates for each Rif1$^{+/+}$ and Rif1$^{-/-}$ ESC line.

(B) Chromosomal location of each viewpoint and associated properties: RT, region associated with RT switches; gene expression changes, region within 1 Mb of a gene whose expression is affected by Rif1 deletion.

(C) Plots showing the total number of same-chromosome contacts per viewpoint.

(D) Contacts for the viewpoint L2 (red arrowhead). The whole of chromosome 16 is shown, with the insets displaying zoom-in views of the RT domain around the viewpoint (intra-RT domain interactions) and of a more distal region (inter-RT domain interactions).

(E) Ratio (fold increase) between the total of positions, with the number of RPMs indicated on the x axis in Rif1$^{-/-}$ versus Rif1$^{+/+}$ (dashed line), averaged over all viewpoints. Positions are grouped by the supporting number of RPMs as indicated on the x axis. The increase of the number of interactions in both the mid- to low-RPM range (10–200) and the high range (200–information [Inf]) in Rif1$^{-/-}$ is significant, as determined by paired t test (**p = 0.006, *p = 0.03). The error bars indicate SDs.

(F) Plots showing the total number of interactions per viewpoint inside the corresponding RT domain.

(G) Ratio (fold increase) between the number of interactions averaged over all viewpoints, as shown in (C) and (F), in Rif1$^{-/-}$ over Rif1$^{+/+}$ ESCs (dashed line), taking into consideration the whole genome or only the interactions taking place within the RT domain (paired t test, **p = 0.006). Error bars indicate SDs.

See also Figures S5 and S6.

and 5D). We reasoned that a loss of structured chromatin contacts could result in an increase of random or quasi-random lower-frequency interactions. Compared to the controls, in Rif1 null ESCs, the viewpoints established additional contacts both at high-frequency (200–information) and at low- to mid-frequency (10–200) RPMs (reads per million), where RPM expresses the calibrated number of reads per position and is therefore roughly proportional to the frequency of identification of each contact within the library (Figure 5E; Figure S5E). However, in agreement with our prediction, the gains were particularly significant within the low- to mid-frequency range.

To explore whether Rif1's role in chromatin organization could be involved in defining the boundaries of a single RT domain, we analyzed whether the 3D organization of the single RT domain is affected by Rif1 deletion. We identified the boundaries of the RT domains encompassing the viewpoints by comparison of RT profiles derived from different cell types (see Supplemental Experimental Procedures). Unlike the number of positions interacting with the viewpoint over the length of the chromosome, contacts within the replication domain are not affected (Figures 5D and 5F). The different outcome of Rif1 deficiency on the total versus the intra-domain contacts (Figures 5D and 5G) suggests that the definition of the replication domain remains unaffected. Instead, the organization of inter-domain interactions has been lost. To independently validate the 4C data and appreciate their qualitative behavior, we performed three further analyses. First, we validated several contact frequency increases by 3C-qPCR (Figure S5F). Second, we used 3D fluorescence in situ hybridization (FISH) to validate the increased proximity of one of the contacts (Figure S5G). Third, an independent analysis of the 4C-seq data with FourCSeq (Figure S6) (see Supplemental Experimental Procedures) (Klein et al., 2015), confirmed a consistent difference between the two conditions. Altogether, these results establish Rif1 as a spatial organizer of chromatin.

Rif1 Controls 3D Chromatin Organization in the G1 Phase

The loosening of spatial control of chromatin interactions observed in Rif1$^{-/-}$ ESCs could be a direct consequence of Rif1 deficiency or a secondary effect of RT changes. To discriminate between these two possibilities, chromatin architecture must be analyzed in the first G1 phase after Rif1 deletion before the first round of replication and the onset of RT deregulation. This experiment is not feasible in ESCs, because they cannot be arrested at any cell cycle stage long enough to obtain sufficient levels of Rif1 deletion, which requires approximately 2 days. We had previously used pMEFs synchronized and deleted in G0 phase to show that failure to re-express Rif1 upon re-entry into G1 phase induces RT deregulation during the first S phase (Figure S7A) (Cornacchia et al., 2012). We therefore employed the same system to try to understand whether the observed altered chromatin organization caused by Rif1 deletion follows or precedes RT deregulation (Figure S7B). Analogous to ESCs, we chose five 4C-seq viewpoints (Figure 6A). Remarkably, we found that Rif1$^{-/-}$ pMEFs, like cycling ESCs, show an increased number of chromosomal positions contacting the viewpoint at a low to medium frequency (10–200 RPMs) and this is already observable during the first G1 phase after deletion (Figures 6B and 6C; Figure S7C).

This synchronization strategy enables us to uncouple the effect of Rif1 deletion on nuclear architecture from its effects on the timing of DNA replication, allowing us to analyze the relation between the changes of chromatin contacts caused in G1 phase by Rif1 deletion and the changes of RT in the following S phase. To this end, we first grouped into TADs the positions interacting with the viewpoint in G1 phase, identified by 4C-seq in synchronized pMEFs. By this classification, we can understand whether the increase of chromatin contacts that we observe in Rif1$^{-/-}$ cells in G1 phase is limited within the same units (TAD) already interacting with the viewpoint in Rif1$^{+/+}$ cells. Alternatively, it could be accompanied by the creation of contacts between the viewpoint and the additional TADs and, if so, we can analyze their RT compared to the viewpoint. Chromatin contacts are normally established among regions (Takebayashi et al., 2012) and more specifically between TADs (Pope et al., 2014) with the same RT. Our analysis reveals that a subset of the specific contacts established in the Rif1$^{-/-}$ cells reflects the creations of novel interactions with additional TADs, while others are common to Rif1$^{+/+}$ and null cells. The RT of the TADs shared between Rif1$^{+/+}$ and Rif1$^{-/-}$ cells is the same as the RT of the viewpoint (Figure 6F, inter-domain RT interactions; Figure S7D, black boxplots), and it shifts toward zero only as Rif1$^{-/-}$ cells undergo S phase (Figure S7D, gray boxplots). This suggests that during the first S phase, the coordination of RT between the TAD containing the viewpoint and its interactors is lost, with some TADs shifting RT like the viewpoint while others do not. However, because the RT of the TADs that specifically interact with the viewpoint only in Rif1$^{-/-}$ cells is already shifted toward zero in the G1 phase (Figure 6D, black bar), the new contacts formed before S phase must be random. These results show that loss of definition of the organization of chromatin contacts induced by Rif1 deficiency precedes the RT changes. More importantly, the newly acquired interactions do not conform to the RT of the viewpoint (Figure 6F, red lines), linking the change of contacts with the following change of RT. Our data support the hypothesis that Rif1-dependent stable and compartmentalized chromatin contacts in G1 phase could instruct a functional RT program in S phase. In addition, the 3D nuclear substructures defined by Rif1 in the G1 phase will undergo a coordinated replication in the following S phase.

Consistent with ESCs, the pMEFs showed no significant change in the number of positions interacting with the viewpoint within the boundaries of the replication domain (Figures 6E and 6F, intra-RT domain interactions), indicating that Rif1 deficiency mostly affects the organization of inter-domain contacts (Figure 6G). In agreement with this conclusion, we observed that RT switches in Rif1 null pMEFs take place in correspondence with the RT domain borders as developmentally defined by the alignment of RT profiles of multiple cell lineages (Figure S7E). This suggests that Rif1 deficiency does not affect the identity of the minimal RT unit.

In summary, by employing this synchronization strategy, we have been able to demonstrate that Rif1 is necessary for the determination of chromatin architecture; i.e., it limits the contacts between RT domains (Figure 6H) in G1 phase,

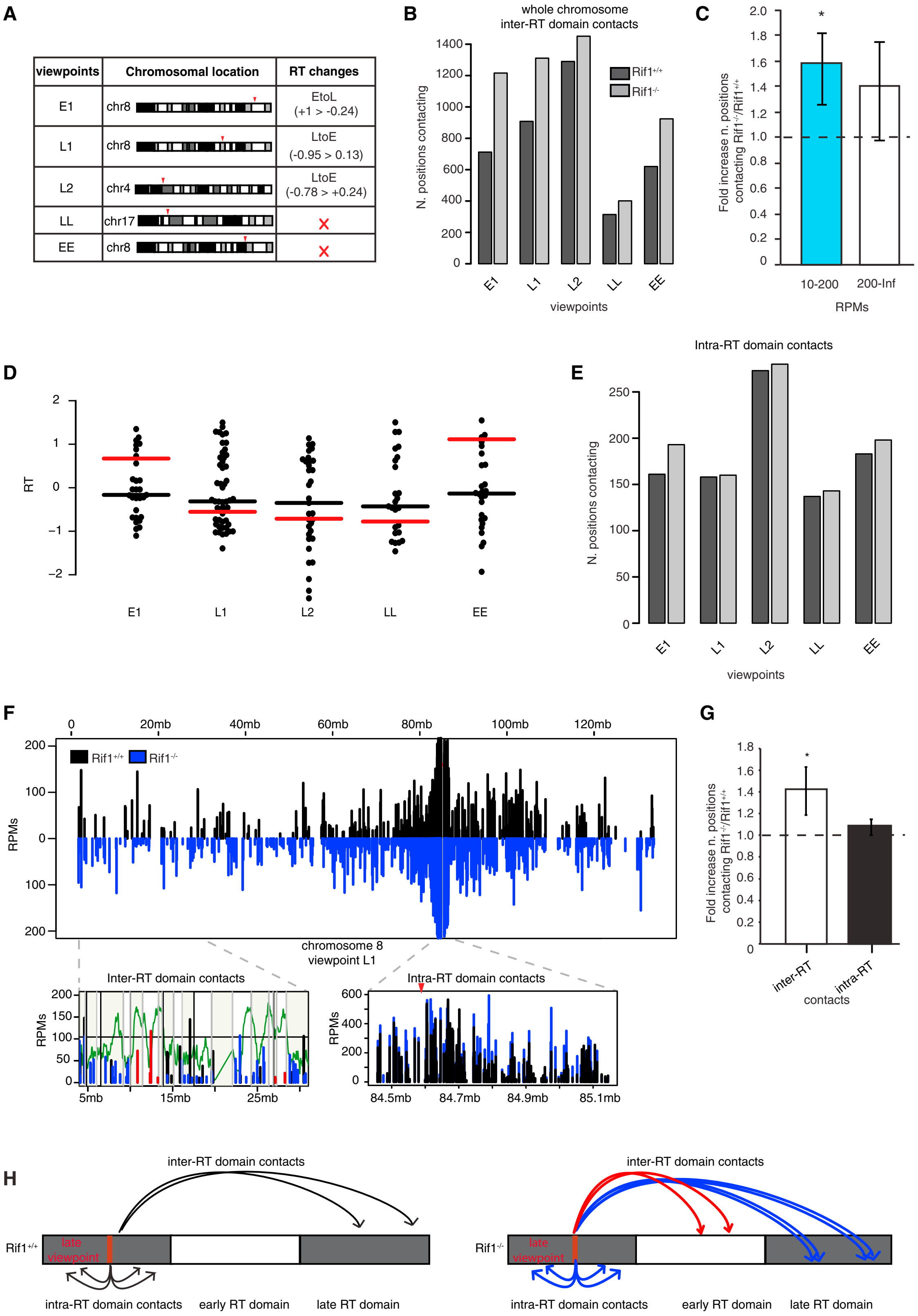

(legend on next page)

independent of replication. These results suggest the possibility that Rif1's earliest function is concurrent with the establishment of RT.

DISCUSSION

Although the temporal organization of the initiation of DNA replication was first reported 57 years ago (Taylor, 1958, 1960), the genetic and molecular control underlying this process remains unclear. Consequently, its biological significance remains undetermined. At the genomic level, RT is dictated by a regional compartmentalization into chromatin domains that replicate simultaneously, known as RT domains (Pope et al., 2014). Here we sought to understand Rif1's molecular function and interrogated its possible involvement in bridging RT and nuclear architecture. Chromatin immunoprecipitation sequencing (ChIP-seq) analysis of Rif1 genome-wide distribution reveals that in ESCs Rif1 displays a domain type of binding (RAD), covering large (~1 Mb), late-replicating genomic regions, which largely overlap with LADs (RAD-LB$^+$). Accordingly, Rif1 both co-localizes and co-immunoprecipitates with Lamin B1. Altogether, these data suggest a possible chromatin-organizing function of Rif1 during RT definition. In agreement with this hypothesis, using 4C-seq analysis, we show here that deletion of Rif1 affects chromatin contacts between different replication domains. This is not an indirect result of cell proliferation in the presence of deregulated RT but rather an immediate consequence of the absence of Rif1. The effect of Rif1 deficiency on nuclear architecture is already evident in synchronized pMEFs in the first G1 phase after deletion, and thus before changes of RT are enacted. Our data indicate that Rif1's effect on RT control ensues from its function of defining chromatin interactions during the G1 phase. Rif1 couples stable nuclear sub-compartments with specific RT. The first consequence of Rif1 deletion is the weakening of nuclear subcompartmentalization, followed by a loss of coordinated replication (e.g., fragmentation of larger replication domains) and the acquisition of new and unstable RT, at least for a fraction of the genome. Within the RADs, we could distinguish two subtypes of late-replicating domains, set apart by different levels of Lamin B1 binding. Rif1 is essential for late replication only in RAD-LB$^-$. These data indicate that additional levels of control, in the context of the structural subnuclear unit defined by the LADs, render RT in these regions resistant to the changes induced by Rif1 deficiency. What distinguishes EtoE (early-to-early) from EtoL domains remains unclear. However, the reproducibility of the early domains switching to late replication suggests the existence of underlying yet unknown regional features.

Recently, it has been shown that Rif1's function during RT control in yeast is mediated through its interaction with PP1 (Davé et al., 2014; Hiraga et al., 2014; Mattarocci et al., 2014). Because mammalian Rif1 also harbors two potential PP1-interaction motifs (Sreesankar et al., 2012) and was identified among PP1α interactors (Trinkle-Mulcahy et al., 2006), it will be interesting to determine whether this interaction has a function in chromatin 3D organization and/or determine the timing of origin firing in mammalian cells. If mammalian Rif1 functions mainly as PP1 adaptor, we can envisage two molecular mechanisms by which Rif1 could link 3D nuclear organization to RT control. Rif1 could be a molecular hub that couples nuclear architecture and RT by affecting both processes in parallel, for example, by targeting PP1 to substrates independently involved in these two processes. Alternatively, Rif1 could instruct RT at two levels. First, by organizing the architecture of early and late domains in the G1 phase, Rif1 could set one level of RT control through 3D compartmentalization of origins. Subsequently, it could schedule their firing by PP1-mediated MCM4 de-phosphorylation. Although more work is needed to clarify these molecular aspects, our data support the latter view, because Rif1 deficiency affects the RT specificity of chromatin contacts before

Figure 6. Rif1 Deletion Affects Nuclear Architecture during the G1 Phase

Contacts are positions consistently identified by the r3Cseq software package analysis of 4C-seq data in the two replicates for each Rif1$^{+/+}$ and Rif1$^{-/-}$ pMEF line in the first G1 phase after deletion. One representative experiment out of two is shown.

(A) Summary of the chromosomal location of each viewpoint and associated features. RT , region associated with RT switches. In pMEFs, there are no gene expression changes induced by Rif1 deletion.

(B) Plots showing the total number of same-chromosome interactions per viewpoint.

(C) Ratio (fold increase) between the total of positions in Rif1$^{-/-}$ versus Rif1$^{+/+}$ (dashed line). Positions are divided in two classes depending on the number of supporting RPMs. The increase of the number of positions in the low- to mid-RPM range (10–200) in Rif1$^{-/-}$ is significant, as determined by paired t test (*p = 0.02). Error bars indicate the SDs.

(D) Distribution of RT (RT = $\log_2$(early/late)) of the 4C-seq contacts within the TADs that are interacting with the indicated viewpoints, specifically in Rif1$^{-/-}$ pMEFs in the G1 phase. The black line indicates their median RT. The red line is the median RT of the TADs that interact with the viewpoint in both synchronized Rif1$^{+/+}$ and Rif1$^{-/-}$ pMEFs in Figure S7D and is placed as a reference to appreciate the difference.

(E) Plots showing the total number of contacts per viewpoint inside the corresponding RT domain.

(F) Contacts for the viewpoint L1 (red arrowhead). The whole of chromosome 8 is shown, with the insets displaying zoom-in views of the RT domain around the viewpoint (right) or a more distal region (left). The insets also show the distributions of TADs in the same regions (gray lines and alternate green shadowing). The RT domain (right) is fully enclosed in a single TAD. In the inset showing the distal region (left), the contacts mapping in TADs that selectively interact with the viewpoint in Rif1$^{-/-}$ cells are shown in red. In green, the RT profile of synchronous Rif1$^{+/+}$ pMEFs is shown.

(G) Ratio between the number of interactions averaged over all viewpoints, as shown in (B) and (E), in Rif1$^{-/-}$ over Rif1$^{+/+}$ pMEFs (dashed line) calculated for the chromosome hosting the viewpoint (in cis) or only inside the RT domain (paired t test, *p = 0.01). Error bars indicate SDs.

(H) Schematic interpretation of the data in (B), (E), and (F), illustrating the gain of inter-RT domain interactions (arrows) and the loss of RT specificity of some acquired contacts in Rif1$^{-/-}$ pMEFs for a putative viewpoint (red). The interactions established by the viewpoint in Rif1$^{+/+}$ are represented by black arrows; the ones established by Rif1$^{-/-}$ cells that fall into TADs shared with Rif1$^{+/+}$ are blue. The new contacts established by the viewpoint exclusively in Rif1$^{-/-}$ cells and that fall into gained TADs are represented by red arrows.

See also Figure S7.

S phase. In our view, this is suggestive of a hierarchical, rather than a parallel, independent effect of Rif1 on nuclear architecture and RT. We propose that Rif1 could define in 3D the late domains at the time of RT establishment in G1 phase and then translate this organization into a threshold for origin initiation in S phase by controlling a regional recruitment of high levels of PP1, as the yeast data also suggest, within the non-LAD late regions.

Overall, our data point to Rif1 being the molecular link between chromatin 3D organization and RT determination. This view is backed by the partial information available concerning its structure. It was recently shown that budding yeast Rif1 can tetramerize (Shi et al., 2013). Multimerization and direct DNA binding have also been shown for the mammalian protein (Sukackaite et al., 2014; Xu et al., 2010; S.C.B.B., unpublished data), suggesting that Rif1 could form a nuclear network organizing chromatin loops and their reciprocal positioning in the nuclear volume. Rif1 has also been shown to function during DNA repair (Buonomo et al., 2009; Chapman et al., 2013; Di Virgilio et al., 2013; Feng et al., 2013; Silverman et al., 2004; Wang et al., 2009; Xu et al., 2010; Zimmermann et al., 2013). Our findings could reconcile these apparently diverse functions attributed to Rif1, as controlling and limiting the number and spatial distribution of chromatin interactions could be part of how Rif1 contributes to regulating repair.

Replication Timing and Transcription: Two Sides of the Same Coin

In the recent years, it has been shown that the organization of DNA RT is a cell-type-specific signature as unique as the gene expression profile and that it undergoes profound remodeling during development. These features parallel the epigenetic and gene expression regulations, but a direct, universal link among these three biological pathways has yet to be found. Although the paradigm of "expressed gene equals early replicating domain" while "silenced gene equals late-replicating domain" is generally true, genome-wide studies have shown that there are many significant exceptions (Hiratani et al., 2010; Rivera-Mulia et al., 2015). Changing the transcriptional status does not always imply a change in RT, and vice versa. Here we show that the establishment of chromatin organization during the G1 phase affects the order of replication of different domains. Changing nuclear architecture induces alterations of RT but in time also translates into changes of the gene expression profile, in agreement with subnuclear positioning having a well-established role in modulating gene expression (Andrulis et al., 1998; Finlan et al., 2008; Mattout et al., 2011; Peric-Hupkes et al., 2010; Reddy et al., 2008; Zullo et al., 2012). Our data also indicate that these changes can take place with the epigenetic landscape hardly being affected, at least in the short term. Nuclear architecture is therefore the common determinant for both gene expression and RT. This finding is an important step toward an understanding of the complex and confusing relationship between the two processes that are linked, though not by causality. Our data imply that RT regulation and nuclear architecture are intricately connected to the extent that it could render difficult to uncouple the question of the biological significance of the RT program from the role of gene expression in establishing cell identity. For future studies addressing this fundamental issue, our work identifies the architectural organization of the Rif1-sensitive fraction of the genome (RAD-LB^{-}) as a uniquely dynamic component in which gene expression regulation and timing of replication probably integrate, converging to the determination of cell identity.

EXPERIMENTAL PROCEDURES

Rif1 ChIP

Rif1$^{FH/FH}$ and Rif1$^{+/+}$ ESCs have been cross-linked first with 2 mM disuccinimidyl glutarate for 45 min and then in 1% formaldehyde (FA) for 10 min. Immunoprecipitation was performed using the Roche anti-hemagglutinin antibody. See Supplemental Experimental Procedures for full details.

ChIP-Seq for Histone Modifications

Rif1$^{-/-}$ and Rif1$^{+/+}$ ESCs have been cross-linked in 1% FA for 10 min. Immunoprecipitation was performed using anti-H3K4me3, anti-H3K9me3, anti-H3K27me3, or anti-H4K20me3 antibodies. See Supplemental Experimental Procedures for full details.

4C-Seq

ESCs or pMEFs were cross-linked in 2% FA. Primary restriction digest was performed by incubation with HindIII, and secondary with was performed by incubation DpnII. Libraries have been sequenced in 100 bp single-end mode. See Supplemental Experimental Procedures for full details.

ACCESSION NUMBERS

The accession number for the chromosome conformation capture data from Rif1 conditional knockout and Rif1 wild-type mouse ESCs reported in this paper is Array Express: E-MTAB-3500. The accession number for the chromosome conformation capture from synchronized Rif1 pMEFs reported in this paper is Array Express: E-MTAB-3505. The accession number for the gene expression profiles from Rif1 conditional knockout and Rif1 wild-type large T immortalized MEFs reported in this paper is Array Express: E-MTAB-3501. The accession number for the gene expression profiles from Rif1 conditional knockout and Rif1 wild-type mouse ESCs reported in this paper is Array Express: E-MTAB-3503. The accession number for the genome-wide occupancy (ChIP-seq) data from Rif1^{FH} in mouse ESCs reported in this paper is Array Express: E-MTAB-3502. The accession number for the RT profiles from Rif1 conditional knockout and Rif1 wild-type mouse ESCs reported in this paper is Array Express: E-MTAB-3506. The accession number for the genome-wide distribution (ChIP-seq) of histone modifications from Rif1 conditional knockout and Rif1 wild-type mouse ESCs reported in this paper is Array Express: E-MTAB-3743.

SUPPLEMENTAL INFORMATION

Supplemental Information includes Supplemental Experimental Procedures, seven figures, and four tables and can be found with this article online at http://dx.doi.org/10.1016/j.molcel.2015.12.001.

AUTHOR CONTRIBUTIONS

R.F. performed most of the experiments and the 4C-seq analysis; S.G. performed 3D FISH, ChIP-qPCR, and ChIP-seq; D.C. contributed to ESC derivation and performed ChIP-seq and immunoprecipitation; V.D. performed the RT experiment and alignment of profiles, helped designing the 4C-seq primers, and analyzed Rif1 distribution in relation to RT switches; A.B.-K. taught the way to perform the ChIP-seq; E.J. and R.L. advised on the 4C-seq analysis; A.B., F.A.K., and W.H. performed the FourCSeq analysis; T.M. and S.D. analyzed the ChIP-seq data; D.M.G. critically read the manuscript and contributed with scientific advice; and T.J. and P.B. hosted members of the S.C.B.B. lab during training. S.C.B.B. designed the experiments and wrote the manuscript.

ACKNOWLEDGMENTS

We would like to acknowledge Violetta Parimbeni for mouse husbandry, Melanie Leuener and Claudia Valeri for technical support, Donal O'Carroll (EMBL Monterotondo) for critically reading the manuscript, and Phil Avner (EMBL Monterotondo) for his support. We would like also to thank Catherine Green and Daniela Morelli (Wellcome Trust Centre for Human Genetics, University of Oxford) for advice on 3D FISH and Vladimir Benes and the Genomic Core Facility and Christian Tischer (Advanced Light Microscopy Facility at EMBL Heidelberg), William Mansfield (Wellcome Trust Centre for Stem Cell Research, University of Cambridge), and Fatima Cavaleri for training in ESCs derivation. We acknowledge Fidel Ramirez for help with deepTools software. This work was technically supported by EMBL's Monterotondo fluorescence-activated cell sorting and microscopy facilities. R.F. was funded by the EMBL Interdisciplinary Postdoc (EIPOD) fellowship under Marie Curie Actions (COFUND). S.C.B.B. thanks the EpiGeneSys Network of Excellence. E.J. is funded by the UK Biotechnology and Biological Sciences Research Council (BBSRC). F.A.K. and W.H. acknowledge support by the European Commission's FP7 project RADIANT. T.J. is supported by the Max Planck Society, and T.J. and T.M. are funded by the German Research Foundation (DFG) concerted research consortium CRC992 "Medical Epigenetics" and the Federal Ministry of Education and Research (BMBF) under the DEEP consortium. This work was supported by PO1 GM085354 to D.M.G.

Received: April 16, 2015
Revised: July 22, 2015
Accepted: November 13, 2015
Published: December 24, 2015

REFERENCES

Andrulis, E.D., Neiman, A.M., Zappulla, D.C., and Sternglanz, R. (1998). Perinuclear localization of chromatin facilitates transcriptional silencing. Nature *394*, 592–595.

Beck, D.B., Burton, A., Oda, H., Ziegler-Birling, C., Torres-Padilla, M.E., and Reinberg, D. (2012). The role of PR-Set7 in replication licensing depends on Suv4-20h. Genes Dev. *26*, 2580–2589.

Besnard, E., Babled, A., Lapasset, L., Milhavet, O., Parrinello, H., Dantec, C., Marin, J.M., and Lemaitre, J.M. (2012). Unraveling cell type-specific and reprogrammable human replication origin signatures associated with G-quadruplex consensus motifs. Nat. Struct. Mol. Biol. *19*, 837–844.

Bianchi, A., and Shore, D. (2007). Early replication of short telomeres in budding yeast. Cell *128*, 1051–1062.

Buonomo, S.B., Wu, Y., Ferguson, D., and de Lange, T. (2009). Mammalian Rif1 contributes to replication stress survival and homology-directed repair. J. Cell Biol. *187*, 385–398.

Cadoret, J.C., Meisch, F., Hassan-Zadeh, V., Luyten, I., Guillet, C., Duret, L., Quesneville, H., and Prioleau, M.N. (2008). Genome-wide studies highlight indirect links between human replication origins and gene regulation. Proc. Natl. Acad. Sci. USA *105*, 15837–15842.

Cayrou, C., Coulombe, P., Vigneron, A., Stanojcic, S., Ganier, O., Peiffer, I., Rivals, E., Puy, A., Laurent-Chabalier, S., Desprat, R., and Méchali, M. (2011). Genome-scale analysis of metazoan replication origins reveals their organization in specific but flexible sites defined by conserved features. Genome Res. *21*, 1438–1449.

Cayrou, C., Coulombe, P., Puy, A., Rialle, S., Kaplan, N., Segal, E., and Méchali, M. (2012). New insights into replication origin characteristics in metazoans. Cell Cycle *11*, 658–667.

Chapman, J.R., Barral, P., Vannier, J.B., Borel, V., Steger, M., Tomas-Loba, A., Sartori, A.A., Adams, I.R., Batista, F.D., and Boulton, S.J. (2013). RIF1 is essential for 53BP1-dependent nonhomologous end joining and suppression of DNA double-strand break resection. Mol. Cell *49*, 858–871.

Cornacchia, D., Dileep, V., Quivy, J.P., Foti, R., Tili, F., Santarella-Mellwig, R., Antony, C., Almouzni, G., Gilbert, D.M., and Buonomo, S.B. (2012). Mouse Rif1 is a key regulator of the replication-timing programme in mammalian cells. EMBO J. *31*, 3678–3690.

Davé, A., Cooley, C., Garg, M., and Bianchi, A. (2014). Protein phosphatase 1 recruitment by Rif1 regulates DNA replication origin firing by counteracting DDK activity. Cell Rep. *7*, 53–61.

Di Virgilio, M., Callen, E., Yamane, A., Zhang, W., Jankovic, M., Gitlin, A.D., Feldhahn, N., Resch, W., Oliveira, T.Y., Chait, B.T., et al. (2013). Rif1 prevents resection of DNA breaks and promotes immunoglobulin class switching. Science *339*, 711–715.

Dileep, V., Ay, F., Sima, J., Vera, D.L., Noble, W.S., and Gilbert, D.M. (2015). Topologically associating domains and their long-range contacts are established during early G1 coincident with the establishment of the replication-timing program. Genome Res. *25*, 1104–1113.

Dimitrova, D.S., and Gilbert, D.M. (1999). The spatial position and replication timing of chromosomal domains are both established in early G1 phase. Mol. Cell *4*, 983–993.

Feng, L., Fong, K.W., Wang, J., Wang, W., and Chen, J. (2013). RIF1 counteracts BRCA1-mediated end resection during DNA repair. J. Biol. Chem. *288*, 11135–11143.

Fernandez-Vidal, A., Guitton-Sert, L., Cadoret, J.C., Drac, M., Schwob, E., Baldacci, G., Cazaux, C., and Hoffmann, J.S. (2014). A role for DNA polymerase θ in the timing of DNA replication. Nat. Commun. *5*, 4285.

Finlan, L.E., Sproul, D., Thomson, I., Boyle, S., Kerr, E., Perry, P., Ylstra, B., Chubb, J.R., and Bickmore, W.A. (2008). Recruitment to the nuclear periphery can alter expression of genes in human cells. PLoS Genet. *4*, e1000039.

Hansen, R.S., Thomas, S., Sandstrom, R., Canfield, T.K., Thurman, R.E., Weaver, M., Dorschner, M.O., Gartler, S.M., and Stamatoyannopoulos, J.A. (2010). Sequencing newly replicated DNA reveals widespread plasticity in human replication timing. Proc. Natl. Acad. Sci. USA *107*, 139–144.

Hardy, C.F., Sussel, L., and Shore, D. (1992). A RAP1-interacting protein involved in transcriptional silencing and telomere length regulation. Genes Dev. *6*, 801–814.

Hayano, M., Kanoh, Y., Matsumoto, S., Renard-Guillet, C., Shirahige, K., and Masai, H. (2012). Rif1 is a global regulator of timing of replication origin firing in fission yeast. Genes Dev. *26*, 137–150.

Hiraga, S., Alvino, G.M., Chang, F., Lian, H.Y., Sridhar, A., Kubota, T., Brewer, B.J., Weinreich, M., Raghuraman, M.K., and Donaldson, A.D. (2014). Rif1 controls DNA replication by directing Protein Phosphatase 1 to reverse Cdc7-mediated phosphorylation of the MCM complex. Genes Dev. *28*, 372–383.

Hiratani, I., Ryba, T., Itoh, M., Yokochi, T., Schwaiger, M., Chang, C.W., Lyou, Y., Townes, T.M., Schübeler, D., and Gilbert, D.M. (2008). Global reorganization of replication domains during embryonic stem cell differentiation. PLoS Biol. *6*, e245.

Hiratani, I., Ryba, T., Itoh, M., Rathjen, J., Kulik, M., Papp, B., Fussner, E., Bazett-Jones, D.P., Plath, K., Dalton, S., et al. (2010). Genome-wide dynamics of replication timing revealed by in vitro models of mouse embryogenesis. Genome Res. *20*, 155–169.

Klein, F.A., Pakozdi, T., Anders, S., Ghavi-Helm, Y., Furlong, E.E., and Huber, W. (2015). FourCSeq: analysis of 4C sequencing data. Bioinformatics *31*, 3085–3091.

Knott, S.R., Peace, J.M., Ostrow, A.Z., Gan, Y., Rex, A.E., Viggiani, C.J., Tavaré, S., and Aparicio, O.M. (2012). Forkhead transcription factors establish origin timing and long-range clustering in *S. cerevisiae*. Cell *148*, 99–111.

Lian, H.Y., Robertson, E.D., Hiraga, S., Alvino, G.M., Collingwood, D., McCune, H.J., Sridhar, A., Brewer, B.J., Raghuraman, M.K., and Donaldson, A.D. (2011). The effect of Ku on telomere replication time is mediated by telomere length but is independent of histone tail acetylation. Mol. Biol. Cell *22*, 1753–1765.

Lin, T., Chao, C., Saito, S., Mazur, S.J., Murphy, M.E., Appella, E., and Xu, Y. (2005). p53 induces differentiation of mouse embryonic stem cells by suppressing Nanog expression. Nat. Cell Biol. *7*, 165–171.

Mantiero, D., Mackenzie, A., Donaldson, A., and Zegerman, P. (2011). Limiting replication initiation factors execute the temporal programme of origin firing in budding yeast. EMBO J. *30*, 4805–4814.

Mattarocci, S., Shyian, M., Lemmens, L., Damay, P., Altintas, D.M., Shi, T., Bartholomew, C.R., Thomä, N.H., Hardy, C.F., and Shore, D. (2014). Rif1 controls DNA replication timing in yeast through the PP1 phosphatase Glc7. Cell Rep. *7*, 62–69.

Mattout, A., Pike, B.L., Towbin, B.D., Bank, E.M., Gonzalez-Sandoval, A., Stadler, M.B., Meister, P., Gruenbaum, Y., and Gasser, S.M. (2011). An EDMD mutation in *C. elegans* lamin blocks muscle-specific gene relocation and compromises muscle integrity. Curr. Biol. *21*, 1603–1614.

Meuleman, W., Peric-Hupkes, D., Kind, J., Beaudry, J.B., Pagie, L., Kellis, M., Reinders, M., Wessels, L., and van Steensel, B. (2013). Constitutive nuclear lamina-genome interactions are highly conserved and associated with A/T-rich sequence. Genome Res. *23*, 270–280.

Patel, P.K., Arcangioli, B., Baker, S.P., Bensimon, A., and Rhind, N. (2006). DNA replication origins fire stochastically in fission yeast. Mol. Biol. Cell *17*, 308–316.

Peace, J.M., Ter-Zakarian, A., and Aparicio, O.M. (2014). Rif1 regulates initiation timing of late replication origins throughout the *S. cerevisiae* genome. PLoS ONE *9*, e98501.

Peric-Hupkes, D., Meuleman, W., Pagie, L., Bruggeman, S.W., Solovei, I., Brugman, W., Gräf, S., Flicek, P., Kerkhoven, R.M., van Lohuizen, M., et al. (2010). Molecular maps of the reorganization of genome-nuclear lamina interactions during differentiation. Mol. Cell *38*, 603–613.

Pope, B.D., Ryba, T., Dileep, V., Yue, F., Wu, W., Denas, O., Vera, D.L., Wang, Y., Hansen, R.S., Canfield, T.K., et al. (2014). Topologically associating domains are stable units of replication-timing regulation. Nature *515*, 402–405.

Qin, H., Yu, T., Qing, T., Liu, Y., Zhao, Y., Cai, J., Li, J., Song, Z., Qu, X., Zhou, P., et al. (2007). Regulation of apoptosis and differentiation by p53 in human embryonic stem cells. J. Biol. Chem. *282*, 5842–5852.

Reddy, K.L., Zullo, J.M., Bertolino, E., and Singh, H. (2008). Transcriptional repression mediated by repositioning of genes to the nuclear lamina. Nature *452*, 243–247.

Rhind, N., and Gilbert, D.M. (2013). DNA replication timing. Cold Spring Harb. Perspect. Biol. *5*, a010132.

Rivera, C., Gurard-Levin, Z.A., Almouzni, G., and Loyola, A. (2014). Histone lysine methylation and chromatin replication. Biochim. Biophys. Acta *1839*, 1433–1439.

Rivera-Mulia, J.C., Buckley, Q., Sasaki, T., Zimmerman, J., Didier, R.A., Nazor, K., Loring, J.F., Lian, Z., Weissman, S., Robins, A.J., et al. (2015). Dynamic changes in replication timing and gene expression during lineage specification of human pluripotent stem cells. Genome Res. *25*, 1091–1103.

Ryba, T., Hiratani, I., Lu, J., Itoh, M., Kulik, M., Zhang, J., Schulz, T.C., Robins, A.J., Dalton, S., and Gilbert, D.M. (2010). Evolutionarily conserved replication timing profiles predict long-range chromatin interactions and distinguish closely related cell types. Genome Res. *20*, 761–770.

Sequeira-Mendes, J., Díaz-Uriarte, R., Apedaile, A., Huntley, D., Brockdorff, N., and Gómez, M. (2009). Transcription initiation activity sets replication origin efficiency in mammalian cells. PLoS Genet. *5*, e1000446.

Shi, T., Bunker, R.D., Mattarocci, S., Ribeyre, C., Faty, M., Gut, H., Scrima, A., Rass, U., Rubin, S.M., Shore, D., and Thomä, N.H. (2013). Rif1 and Rif2 shape telomere function and architecture through multivalent Rap1 interactions. Cell *153*, 1340–1353.

Shimi, T., Butin-Israeli, V., Adam, S.A., and Goldman, R.D. (2010). Nuclear lamins in cell regulation and disease. Cold Spring Harb. Symp. Quant. Biol. *75*, 525–531.

Silverman, J., Takai, H., Buonomo, S.B., Eisenhaber, F., and de Lange, T. (2004). Human Rif1, ortholog of a yeast telomeric protein, is regulated by ATM and 53BP1 and functions in the S-phase checkpoint. Genes Dev. *18*, 2108–2119.

Sreesankar, E., Senthilkumar, R., Bharathi, V., Mishra, R.K., and Mishra, K. (2012). Functional diversification of yeast telomere associated protein, Rif1, in higher eukaryotes. BMC Genomics *13*, 255.

Sukackaite, R., Jensen, M.R., Mas, P.J., Blackledge, M., Buonomo, S.B., and Hart, D.J. (2014). Structural and biophysical characterization of murine rif1 C terminus reveals high specificity for DNA cruciform structures. J. Biol. Chem. *289*, 13903–13911.

Takebayashi, S., Dileep, V., Ryba, T., Dennis, J.H., and Gilbert, D.M. (2012). Chromatin-interaction compartment switch at developmentally regulated chromosomal domains reveals an unusual principle of chromatin folding. Proc. Natl. Acad. Sci. USA *109*, 12574–12579.

Tanaka, S., Nakato, R., Katou, Y., Shirahige, K., and Araki, H. (2011). Origin association of Sld3, Sld7, and Cdc45 proteins is a key step for determination of origin-firing timing. Curr. Biol. *21*, 2055–2063.

Taylor, J.H. (1958). The mode of chromosome duplication in *Crepis capillaris*. Exp. Cell Res. *15*, 350–357.

Taylor, J.H. (1960). Asynchronous duplication of chromosomes in cultured cells of Chinese hamster. J. Biophys. Biochem. Cytol. *7*, 455–464.

Tazumi, A., Fukuura, M., Nakato, R., Kishimoto, A., Takenaka, T., Ogawa, S., Song, J.H., Takahashi, T.S., Nakagawa, T., Shirahige, K., and Masukata, H. (2012). Telomere-binding protein Taz1 controls global replication timing through its localization near late replication origins in fission yeast. Genes Dev. *26*, 2050–2062.

Teytelman, L., Thurtle, D.M., Rine, J., and van Oudenaarden, A. (2013). Highly expressed loci are vulnerable to misleading ChIP localization of multiple unrelated proteins. Proc. Natl. Acad. Sci. USA *110*, 18602–18607.

Trinkle-Mulcahy, L., Andersen, J., Lam, Y.W., Moorhead, G., Mann, M., and Lamond, A.I. (2006). Repo-Man recruits PP1 gamma to chromatin and is essential for cell viability. J. Cell Biol. *172*, 679–692.

Wang, H., Zhao, A., Chen, L., Zhong, X., Liao, J., Gao, M., Cai, M., Lee, D.H., Li, J., Chowdhury, D., et al. (2009). Human RIF1 encodes an anti-apoptotic factor required for DNA repair. Carcinogenesis *30*, 1314–1319.

Wu, P.Y., and Nurse, P. (2009). Establishing the program of origin firing during S phase in fission Yeast. Cell *136*, 852–864.

Xu, D., Muniandy, P., Leo, E., Yin, J., Thangavel, S., Shen, X., Ii, M., Agama, K., Guo, R., Fox, D., 3rd, et al. (2010). Rif1 provides a new DNA-binding interface for the Bloom syndrome complex to maintain normal replication. EMBO J. *29*, 3140–3155.

Yaffe, E., Farkash-Amar, S., Polten, A., Yakhini, Z., Tanay, A., and Simon, I. (2010). Comparative analysis of DNA replication timing reveals conserved large-scale chromosomal architecture. PLoS Genet. *6*, e1001011.

Yamazaki, S., Ishii, A., Kanoh, Y., Oda, M., Nishito, Y., and Masai, H. (2012). Rif1 regulates the replication timing domains on the human genome. EMBO J. *31*, 3667–3677.

Yamazaki, S., Hayano, M., and Masai, H. (2013). Replication timing regulation of eukaryotic replicons: Rif1 as a global regulator of replication timing. Trends Genet. *29*, 449–460.

Yoshida, K., Bacal, J., Desmarais, D., Padioleau, I., Tsaponina, O., Chabes, A., Pantesco, V., Dubois, E., Parrinello, H., Skrzypczak, M., et al. (2014). The histone deacetylases sir2 and rpd3 act on ribosomal DNA to control the replication program in budding yeast. Mol. Cell *54*, 691–697.

Zimmermann, M., Lottersberger, F., Buonomo, S.B., Sfeir, A., and de Lange, T. (2013). 53BP1 regulates DSB repair using Rif1 to control 5′ end resection. Science *339*, 700–704.

Zullo, J.M., Demarco, I.A., Piqué-Regi, R., Gaffney, D.J., Epstein, C.B., Spooner, C.J., Luperchio, T.R., Bernstein, B.E., Pritchard, J.K., Reddy, K.L., and Singh, H. (2012). DNA sequence-dependent compartmentalization and silencing of chromatin at the nuclear lamina. Cell *149*, 1474–1487.

Molecular Cell

Supplemental Information

Nuclear Architecture Organized by Rif1 Underpins the Replication-Timing Program

Rossana Foti, Stefano Gnan, Daniela Cornacchia, Vishnu Dileep, Aydan Bulut-Karslioglu, Sarah Diehl, Andreas Buness, Felix A. Klein, Wolfgang Huber, Ewan Johnstone, Remco Loos, Paul Bertone, David M. Gilbert, Thomas Manke, Thomas Jenuwein, and Sara C.B. Buonomo

Fig. S1

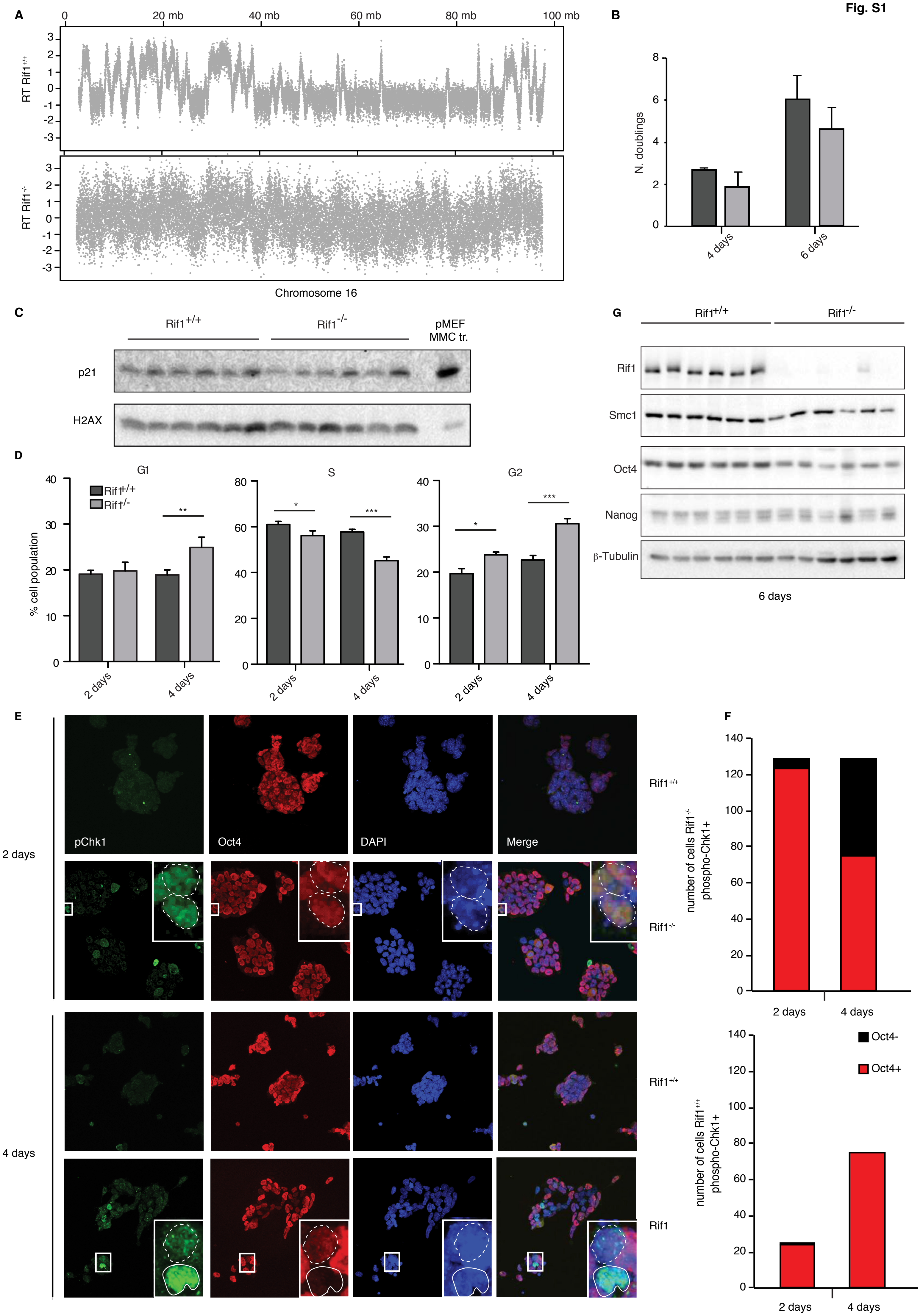
A
0
20 mb
40 mb
60 mb
80 mb
100 mb
RT Rif1+/+
RT Rif1-/-
Chromosome 16
B
N. doublings
4 days
6 days
C
Rif1+/+
Rif1-/-
pMEF
MMC tr.
p21
H2AX
D
G1
S
G2
Rif1+/+
Rif1-/-
% cell population
2 days
4 days
G
Rif1+/+
Rif1-/-
Rif1
Smc1
Oct4
Nanog
β-Tubulin
6 days
E
2 days
4 days
pChk1
Oct4
DAPI
Merge
Rif1+/+
Rif1-/-
Rif1
F
number of cells Rif1-/- phospho-Chk1+
number of cells Rif1+/+ phospho-Chk1+
Oct4-
Oct4+
2 days
4 days

Fig. S2

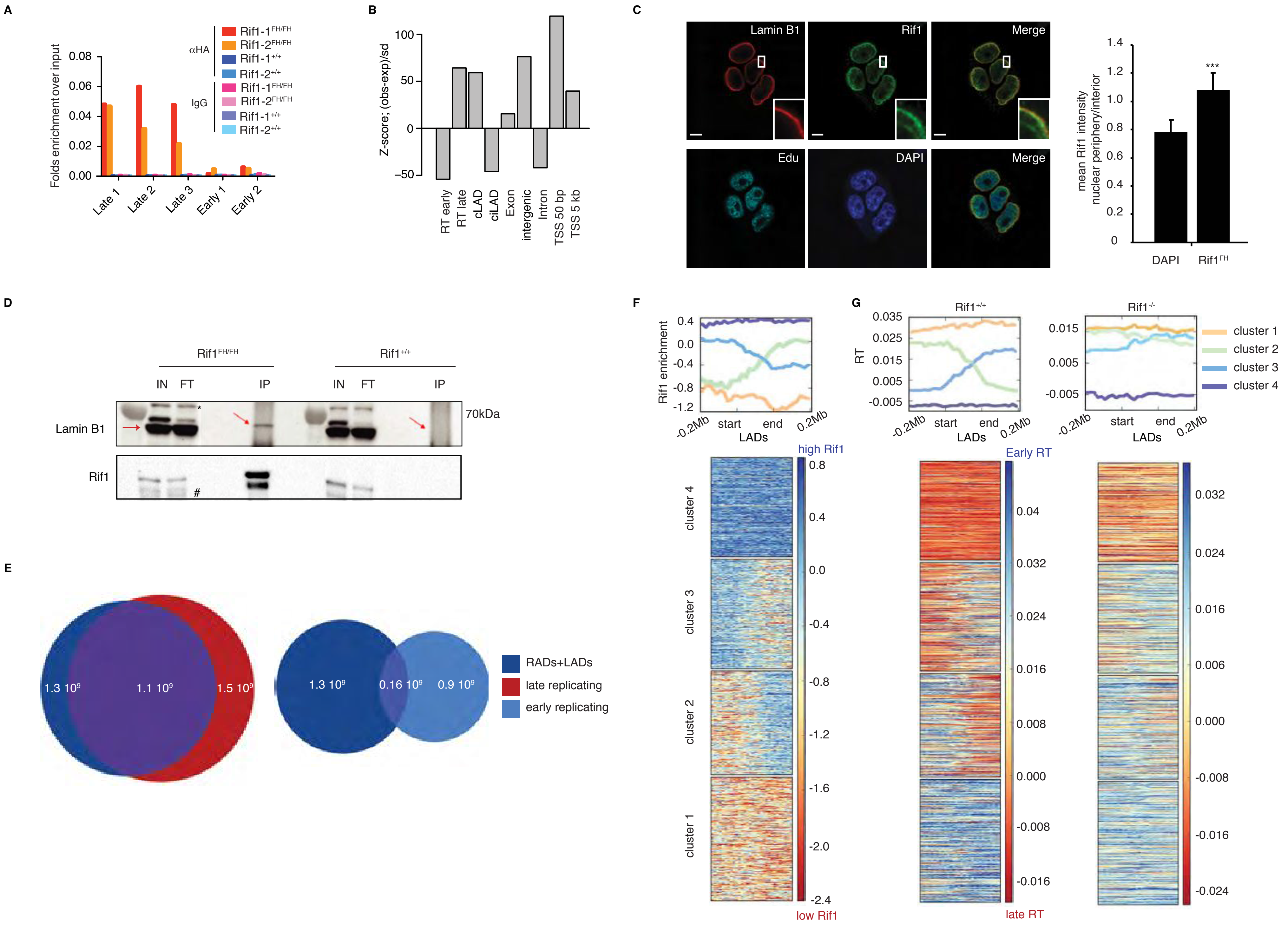

A
Folds enrichment over input
0.08
0.06
0.04
0.02
0.00
Late 1
Late 2
Late 3
Early 1
Early 2
αHA
IgG
Rif1-1 FH/FH
Rif1-2 FH/FH
Rif1-1 +/+
Rif1-2 +/+
B
Z-score; (obs-exp)/sd
100
50
0
−50
RT early
RT late
cLAD
ciLAD
Exon
intergenic
Intron
TSS 50 bp
TSS 5 kb
C
Lamin B1
Rif1
Merge
Edu
DAPI
mean Rif1 intensity nuclear periphery/interior
1.4
1.2
1.0
0.8
0.6
0.4
0.2
0
***
DAPI
Rif1 FH
D
Rif1 FH/FH
Rif1 +/+
IN
FT
IP
Lamin B1
Rif1
70kDa
*
#
E
1.3 10⁹
1.1 10⁹
1.5 10⁹
0.16 10⁹
0.9 10⁹
RADs+LADs
late replicating
early replicating
F
Rif1 enrichment
0.4
0.0
-0.4
-0.8
-1.2
-0.2Mb
start
end
0.2Mb
LADs
high Rif1
low Rif1
cluster 4
cluster 3
cluster 2
cluster 1
0.8
-1.6
-2.0
-2.4
G
Rif1 +/+
Rif1 -/-
RT
0.035
0.025
0.015
0.005
-0.005
cluster 1
cluster 2
cluster 3
cluster 4
Early RT
late RT
0.04
0.032
0.024
0.016
0.008
0.000
-0.008
-0.016
-0.024
0.006

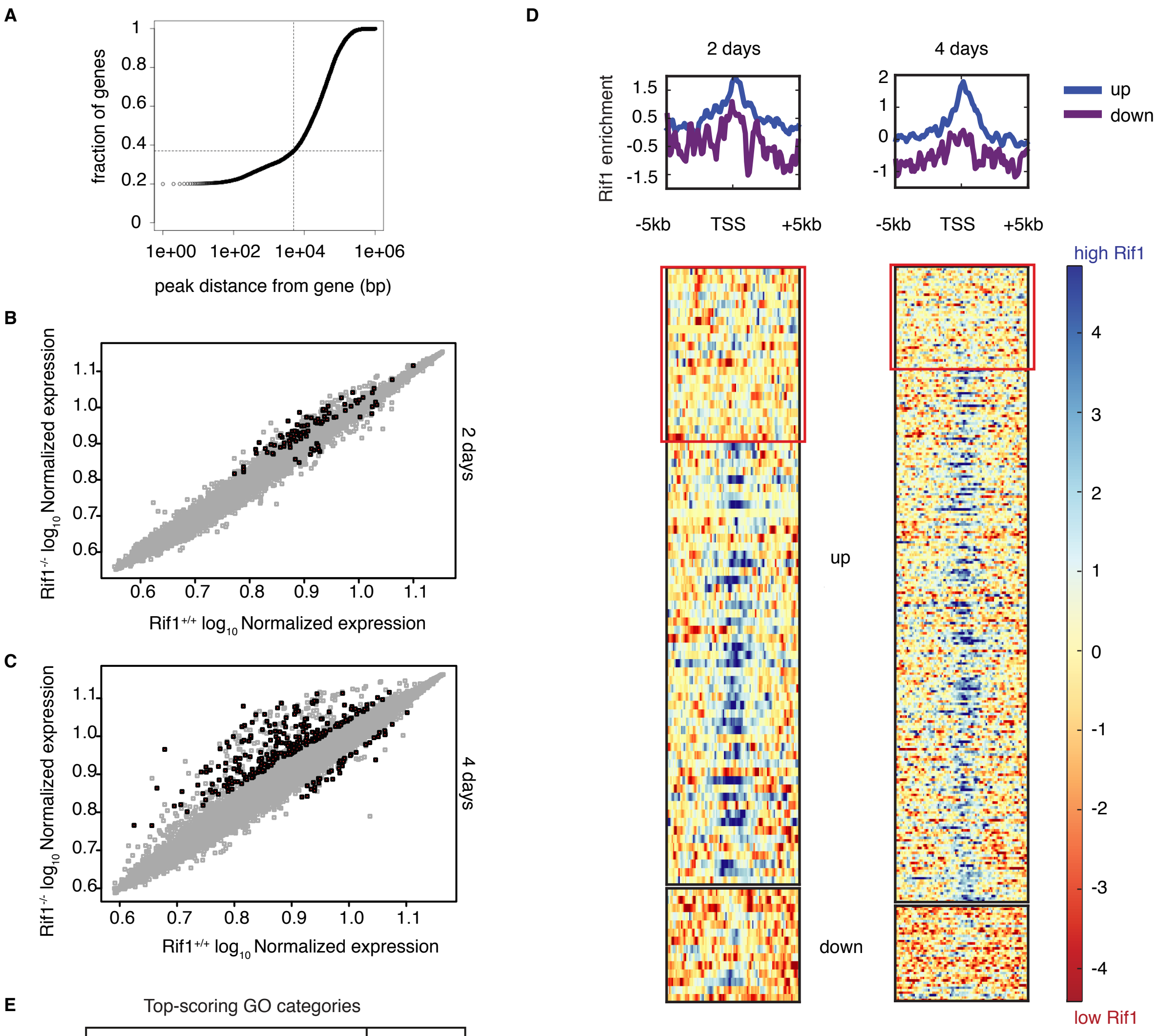

E

Top-scoring GO categories

GO categories	P
Metabolic processes	0.09
mRNA splicing	0.4
RNA processing	0.7
Developmental processes	1.00

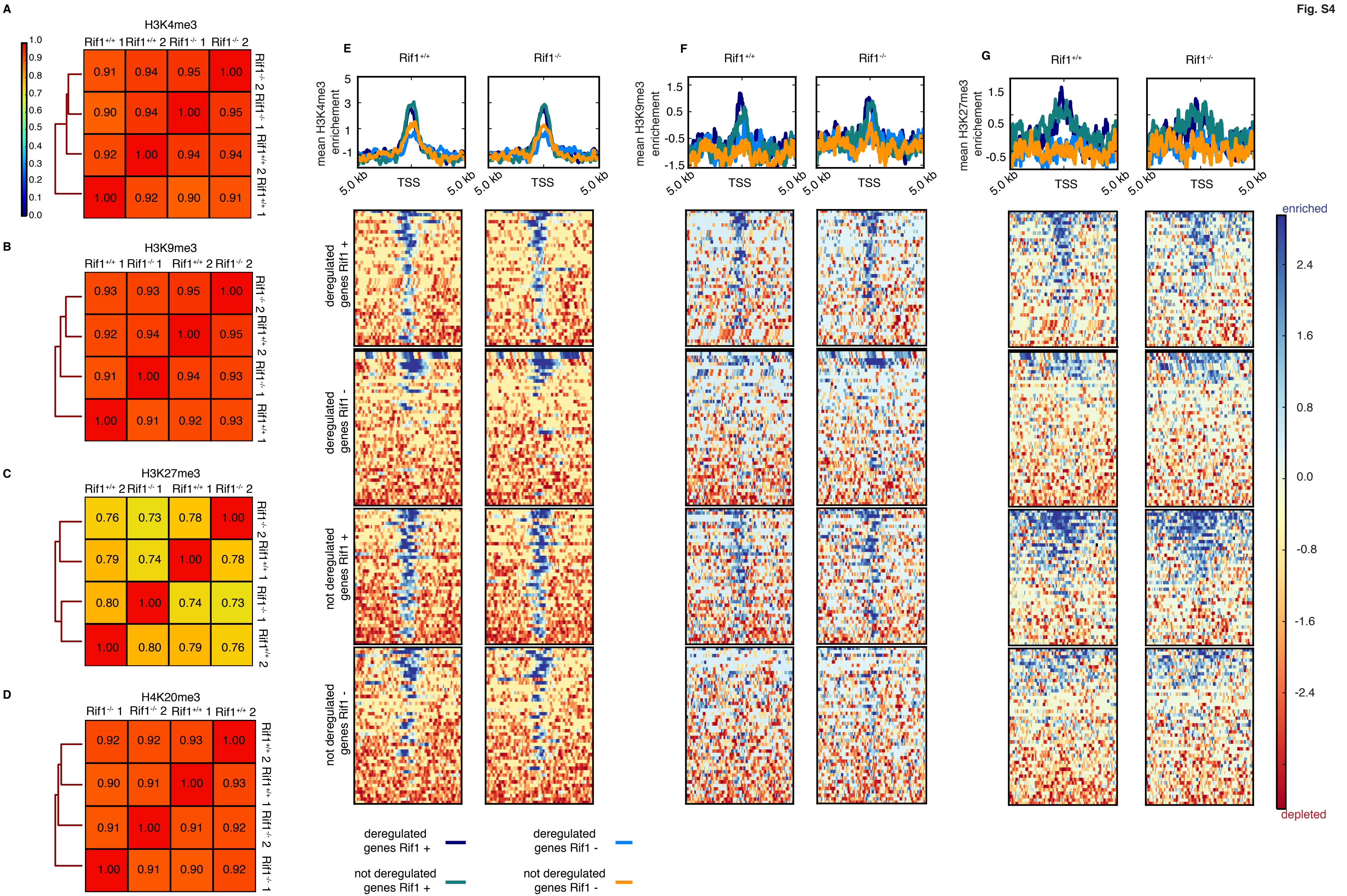
A
H3K4me3
Rif1+/+ 1 Rif1+/+ 2 Rif1-/- 1 Rif1-/- 2
B
H3K9me3
Rif1+/+ 1 Rif1-/- 1 Rif1+/+ 2 Rif1-/- 2
C
H3K27me3
Rif1+/+ 2 Rif1-/- 1 Rif1+/+ 1 Rif1-/- 2
D
H4K20me3
Rif1-/- 1 Rif1-/- 2 Rif1+/+ 1 Rif1+/+ 2
E
F
G
Rif1+/+
Rif1-/-
mean H3K4me3 enrichement
mean H3K9me3 enrichement
mean H3K27me3 enrichement
5.0 kb
TSS
deregulated genes Rif1 +
deregulated genes Rif1 -
not deregulated genes Rif1 +
not deregulated genes Rif1 -
enriched
depleted

Fig. S5

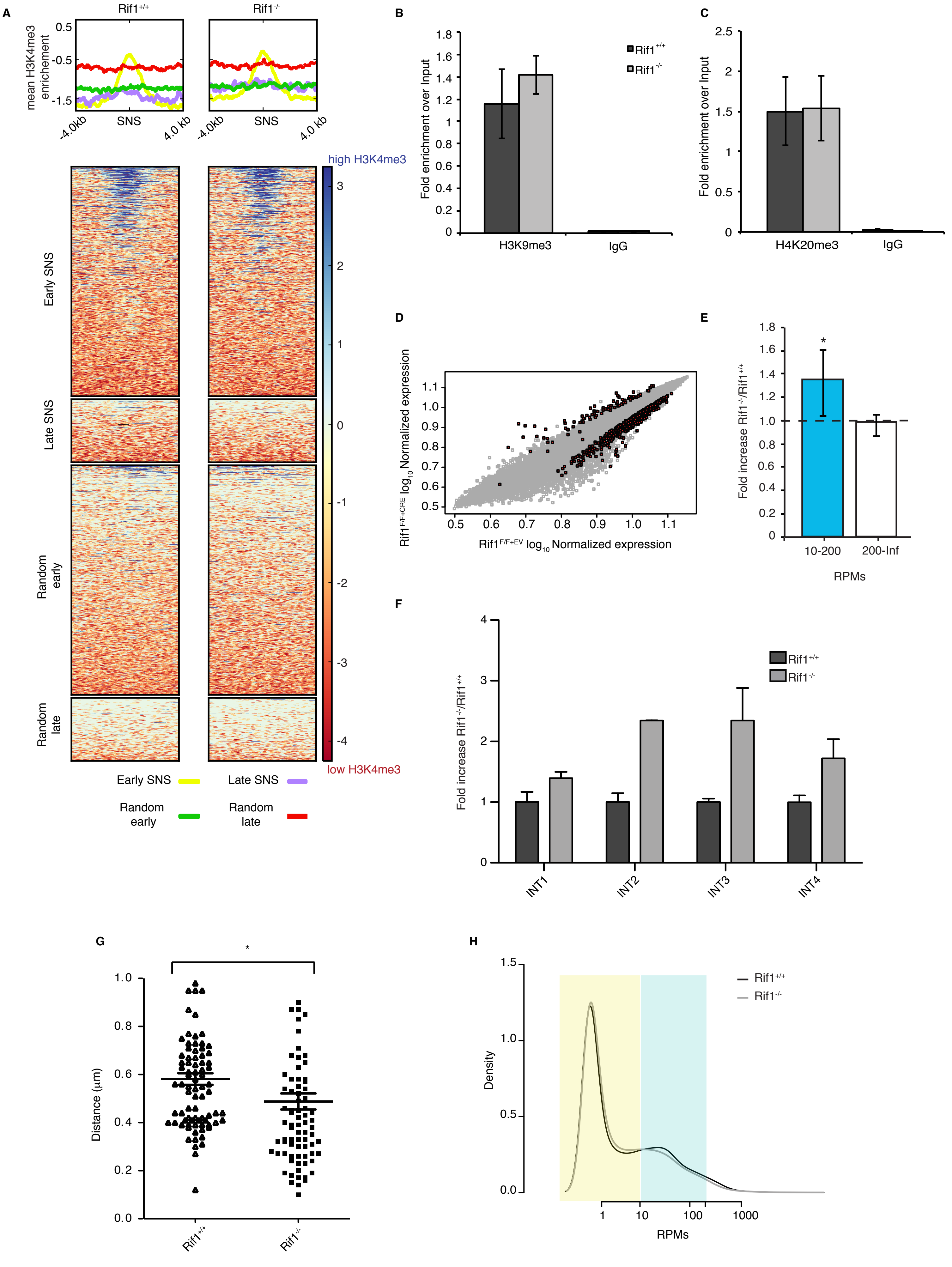

A
Rif1+/+
Rif1-/-
mean H3K4me3 enrichement
SNS
-4.0kb
4.0 kb
high H3K4me3
low H3K4me3
Early SNS
Late SNS
Random early
Random late
B
Fold enrichment over Input
H3K9me3
IgG
Rif1+/+
Rif1-/-
C
Fold enrichment over Input
H4K20me3
IgG
D
Rif1F/F+CRE log10 Normalized expression
Rif1F/F+EV log10 Normalized expression
E
Fold increase Rif1-/-/Rif1+/+
10-200
200-Inf
RPMs
F
Fold increase Rif1-/-/Rif1+/+
Rif1+/+
Rif1-/-
INT1
INT2
INT3
INT4
G
Distance (μm)
Rif1+/+
Rif1-/-
H
Density
RPMs
Rif1+/+
Rif1-/-

Fig. S6

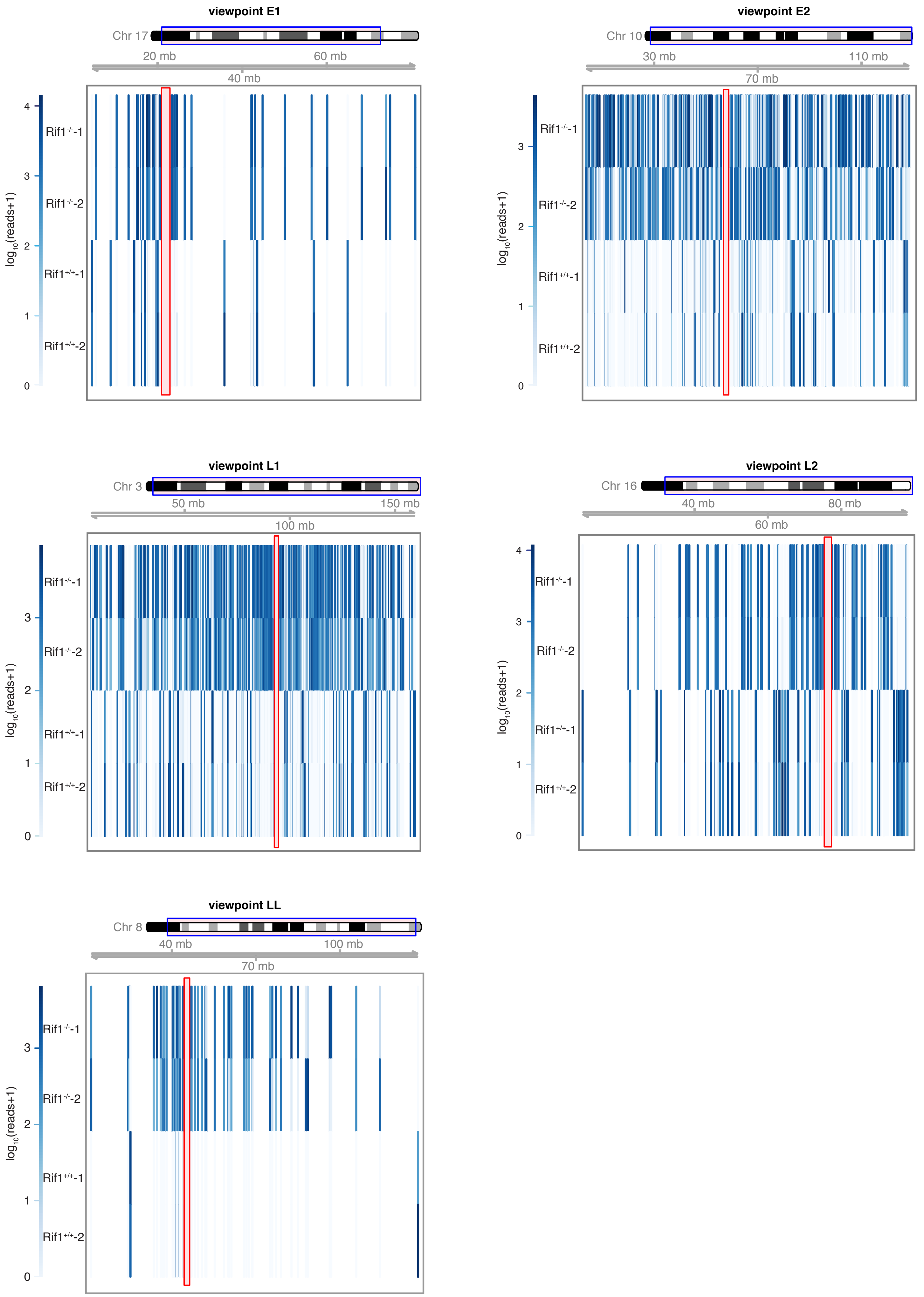
viewpoint E1
Chr 17
20 mb
60 mb
40 mb
log10(reads+1)
Rif1-/--1
Rif1-/--2
Rif1+/+-1
Rif1+/+-2
viewpoint E2
Chr 10
30 mb
110 mb
70 mb
viewpoint L1
Chr 3
50 mb
150 mb
100 mb
viewpoint L2
Chr 16
40 mb
80 mb
60 mb
viewpoint LL
Chr 8
40 mb
100 mb
70 mb

Fig. S7

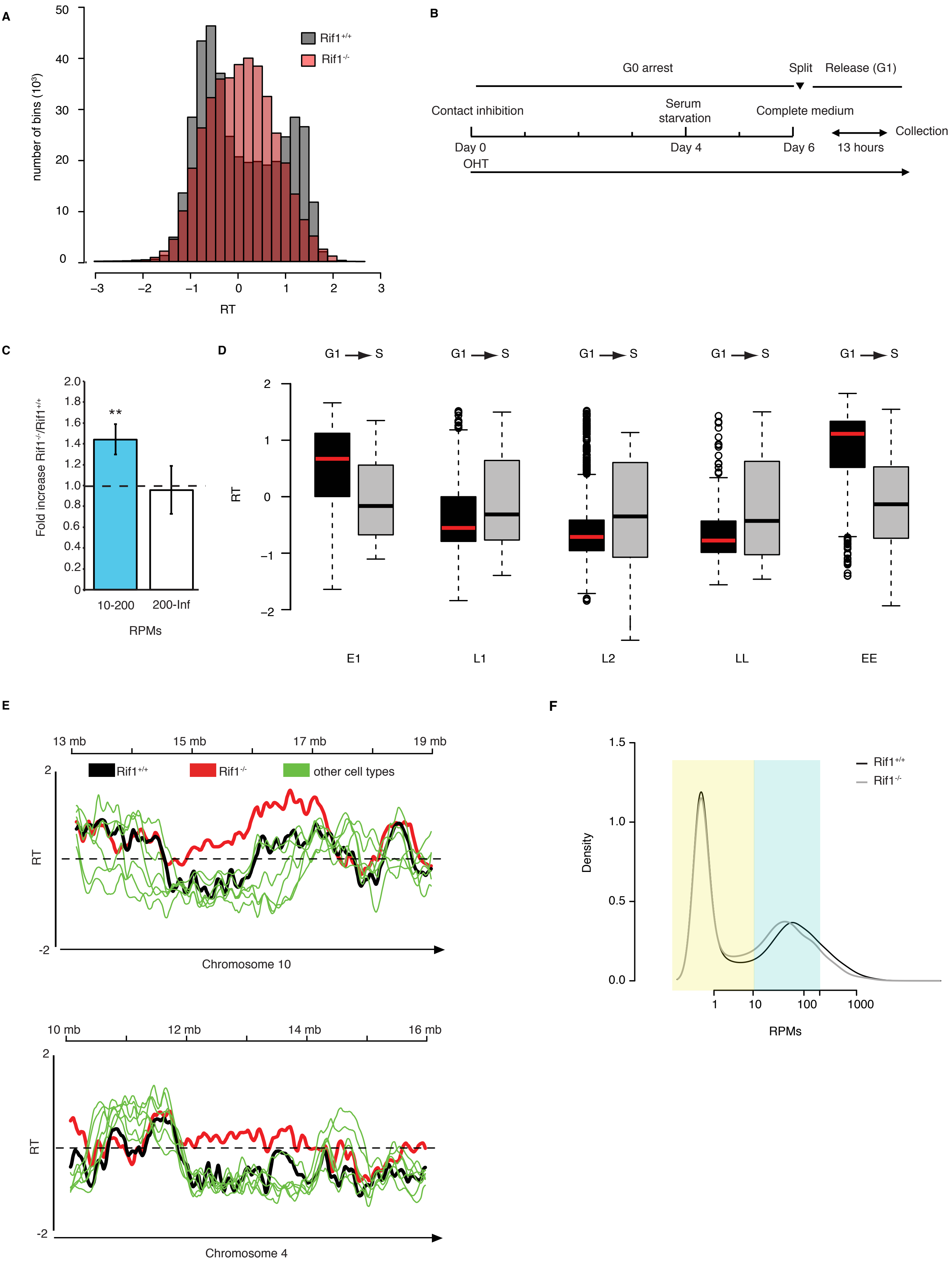
A
number of bins (10³)
Rif1+/+
Rif1-/-
RT
B
G0 arrest
Split
Release (G1)
Contact inhibition
Serum starvation
Complete medium
Collection
Day 0
Day 4
Day 6
13 hours
OHT
C
Fold increase Rif1-/-/Rif1+/+
**
10-200
200-Inf
RPMs
D
G1 → S
RT
E1
L1
L2
LL
EE
E
13 mb
15 mb
17 mb
19 mb
Rif1+/+
Rif1-/-
other cell types
RT
Chromosome 10
10 mb
12 mb
14 mb
16 mb
RT
Chromosome 4
F
Density
Rif1+/+
Rif1-/-
RPMs

Supplemental Figure Legends

Figure S1: Rif1 null ESCs and LT-MEFs do not arrest upon Rif1 deletion and ESCs start differentiating upon a sustained DNA damage response. Related to Fig. 1. A Replication-timing plot of normalized, non-smoothed data from one representative Rif1$^{+/+}$ and one Rif1$^{-/-}$ ESC line. **B** To determine if the G1/S checkpoint function is responsible for the decreased proliferation of pMEFs in response to Rif1 deletion, we infected pMEFs with SV40 large-T antigen (LT) to abolish this checkpoint. Rif1$^{+/+}$ or Rif1 conditional (Rif1$^{F/F}$) cells infected with an empty (EV) or Cre-encoding (Cre) vector were compared. Shown are the averages from triplicates of three independent Rif1$^{+/+}$ versus Rif1$^{-/-}$ LT-MEFs, from one representative experiment out of two. Error bars indicate standard deviations. LT-immortalization alleviates the proliferation arrest of Rif1 deficient pMEF but instead activates the DNA replication checkpoint, as seen in ESCs (Buonomo et al., 2009). **C** Western blot showing p21 levels upon Rif1 deletion in six independent Rif1$^{-/-}$ and Rif1$^{+/+}$ cells lines four days after Cre induction. H2AX is used as loading control. pMEF treated with Mitomycin C (MMC) are used as positive control of p21 induction. Rif1 deletion in ESCs does not induce activation of the G1/S checkpoint, as monitored by p21 upregulation. **D** Analysis of cell cycle distribution from six independent Rif1$^{+/+}$ versus Rif1$^{-/-}$ ESCs. The results of three independent experiments are shown. Cells were pulsed for 1 hour with EdU, fixed and analyzed by FACS. Error bars represent standard deviations. t-test was used to calculate P values (**P<0.001; *** P<0.0001). **E** Two and four days after Cre-induction cells were stained for Chk1^{Ser345} phosphorylated (green) and Oct4 (red). Insets show examples of single cells. Four days after Cre-induction Rif1$^{-/-}$ ESCs with higher levels of damage start down-regulating Oct4. **F** Distribution of the

Oct4 positive and negative ESCs among Chk1^{Ser345} phosphorylated positive population. 130 cells showing Chk1^{Ser345} phosphorylated signal were counted for Rif1$^{-/-}$ ESCs. For Rif1$^{+/+}$ the entire population of cells positive for Chk1^{Ser345} phosphorylated signal detectable on the slide was counted. **G** Western blot analysis of Oct4 and Nanog levels six days after Rif1 deletion in six independent Rif1$^{-/-}$ and Rif1$^{+/+}$ ESC lines shows that the loss of pluripotency becomes evident in the population at this later stage. These results explain the loss of pluripotency previously reported in long-term Rif1 knock-down ESCs (Dan et al., 2014).

Figure S2: Rif1 genome-wide distribution and lamin B1. Related to Fig. 2. A qPCR confirmation of selected Rif1 binding sites on two independent Rif1$^{FH/FH}$ and Rif1$^{+/+}$ ESC lines. The enrichment of the signal in the sample immunoprecipitated either by anti-HA antibody or by IgG control is normalized over the input. The primers were designed over three regions that showed enrichment of Rif1 in the ChIP-seq (Late 1 to 3) and two regions that did not (Early 1 and 2). **B** Enrichment analysis of Rif1 peaks in the indicated regions for one representative ESC line out of three analyzed. The expected values were calculated based on shuffled peaks. **C** Immunofluorescence for lamin B1 and Rif1. In Cyan EdU staining identifies S-phase cells. DAPI in blue. Scale bar=5μm. The bar chart shows the quantification of Rif1 enrichment at the nuclear periphery versus the interior. Quantification of the DAPI distribution has been used as negative control. The error bars indicate standard deviations. (****P*<0.0001 was calculated by t-test). Ten different fields have been analyzed. **D** Rif1 immunoprecipitates lamin B1. IN=input, 1% of total extract; FT=flow through; IP=immunoprecipitated proteins. *=cross-reacting band. #= Rif1 degradation product. The red arrow indicates lamin B1 band. **E** Venn diagram indicating

the overlap in base pairs between LADs+RADs and late or early replicating regions. **F** Meta-analysis of Rif1 distribution (Rif1 enrichment=log_2(Rif1 enrichment/input)) with respect to randomized regions (LADs shuffled over the genome). As for the LADs (Fig. 2D), an unsupervised clustering yields 4 distinct classes of random regions (with high or low Rif1 signal and the respective boundaries). However, compared to Fig. 2D, the cluster sizes are inverted, pointing to the preferential association of LADs with strong Rif1 signal. Notice that the RT-behavior of random regions is also markedly different from LADs. **G** Replication timing distribution (RT=log_2(early/late)) is shown for Rif1$^{+/+}$ and Rif1$^{-/-}$ ESCs, with respect to the randomized regions and in the same order as in **F**.

Figure S3: Rif1 does not control gene expression directly. Related to Fig. 4. A For each UCSC gene the Rif1 signal with the shortest distance was determined. This plot shows the cumulative distribution of these distances. Notice that 20% of all TSS overlap directly with a peak (distance=0). Data from one representative out of three ESC lines analyzed. **B and C** Time course of gene expression changes upon Rif1 deletion in ESCs. Microarray analysis of total RNA two (**B**) and four days (**C**) after the induction of Rif1 deletion. Scatter plots show normalized expression values of RNA between four Rif1$^{-/-}$ and four Rif1$^{+/+}$ cell lines. Each of the red squares represents a transcript whose level varies more then two folds (log fold change >1) with an associated $P<0.05$. Only 0.7% and 2.5% of the Rif1-bound TSSs are associated with genes whose expression is affected by Rif1 deletion after two and four days of Cre-treatment respectively. **D** Heatmaps showing the distribution of Rif1-signal in a 10kb region around the TSSs of those genes whose expression is up-regulated (up) or down-regulated (down) upon Rif1 deletion (two and four days after Cre induction). The genes are sorted from the most (top) to the least

deregulated (bottom). Note that the top-deregulated genes indicated by the red boxes do not display Rif1 associated to the TSS. **E** Statistical analysis of the distribution of deregulated genes among GO categories from PANTHER. Table summarizing the GO categories most enriched among the differentially expressed genes. We could not identify any gene ontology (GO) category-specific enrichment suggestive of a link between gene expression profile changes and the DNA damage response or differentiation processes.

Figure S4: Rif1 deletion does not immediately impact on the epigenetic landscape. Related to Fig. 4.

A-D Spearmann correlation plots of the genome-wide distribution of H3K4me3 (**A**), H3K9me3 (**B**), H3K27me3 (**C**), H4K20me3 (**D**) calculated comparing data from two $Rif1^{+/+}$ and two $Rif1^{-/-}$ lines. **E-G** Heatmaps showing +/- 5kb around TSSs the distribution for H3K4me3 (**E**), H3K9me3 (**F**) and H3K27me3 (**G**) respectively in one representative $Rif1^{+/+}$ and $Rif1^{-/-}$ ESC lines out of two analyzed. TSSs are stratified accordingly to Rif1 presence and clustered on the basis of the association with genes differentially affected upon Rif1 deletion.

Figure S5: Rif1 deletion does not affect H3K4me3 distribution around SNSs or H3K9me3 and H4K20me3 enrichments at major satellites, but increases the number of chromatin contacts. Related to Fig. 4, Fig. S3 and Fig. 5. A Heatmaps showing H3K4me3 distribution +/- 5kb around SNSs mapped on chromosome 11 in one representative $Rif1^{+/+}$ and $Rif1^{-/-}$ line out of two analyzed. SNSs were clustered accordingly to their replication timing. **B and C**. ChIP-qPCR quantification of H3K9me3 (**B**) and H4K20me3 (**C**) levels at major satellites in $Rif1^{-/-}$ and $Rif1^{+/+}$ ESCs. The average of three ESC lines per genotype in two independent experiments is shown. The error bars

represent standard deviations. **D** LT-MEF deficient for Rif1 function display mildly de-regulated gene expression. Microarray analysis of total RNA from three independent Rif1$^{F/F}$ lines infected with either Cre-encoding or empty vectors. The scatter plot shows normalized expression values of RNA. Each red square represents a transcript whose level varies more then two folds (log fold change >1) with an associated P<0.05 between Rif1 wild type (Rif1$^{F/F}$+EV) and null (Rif1$^{F/F}$+Cre) cells. **E** Ratio (fold increase) of the average number of fragments containing the indicated RPM's ranges in two individually considered Rif1$^{-/-}$ versus two Rif1$^{+/+}$ (dashed line) ESCs calculated over all the viewpoints. The increase of fragments containing 10-200 RPMs in Rif1$^{-/-}$ is statistically significant as calculated by two ways ANOVA test based on conditions and viewpoints (*P=0.04). The error bars indicate standard deviations. In this figure all fragments which belong to either class (10-200 or above 200RPMs) are used for the calculation, while Fig. 5E is based on those interactions consistently identified in all replicates within a condition as calculated by the r3Cseq software package. **F** 3C-qPCR confirmation of four selected interactions identified by 4C-Seq for two viewpoints. In each case Rif1$^{-/-}$ ESCs show increased contacts, confirming the 4C-Seq results. The data were obtained from two independent Rif1$^{+/+}$ and Rif1$^{-/-}$ ESC lines. The Rif1$^{-/-}$ enrichment normalized over the input is expressed as fold increase over the normalized Rif1$^{+/+}$ enrichment. **G** Analysis by 3D FISH of the average distance between the two probes indicated in the Supplemental Experimental Procedures, one on the viewpoint E1 on chromosome 17 and the other on one of its most frequent interactions gained in Rif1$^{-/-}$ ESCs (*P=0.0125). Analysis of the average distance between these two probes in Rif1$^{-/-}$ versus Rif1$^{+/+}$ ESCs has confirmed the results obtained by 4C-Seq, showing a decreased average distance between the

viewpoint E1 and the distal interacting point. The analysis was performed blindly and the error bars indicate the standard error median. Wilcoxon test was used to calculate the P value. **H** Relative distribution of the fragments containing the indicated RPMs obtained by combining all ESCs $Rif1^{+/+}$ (black line) and $Rif1^{-/-}$ (grey line) reads, over all the viewpoints, normalizing the library size to 1 million. The bimodal distribution allows the identification of 10 RPMs as cutoff to exclude interactions falling within the noise range (yellow shadowing). The blue shadowing corresponds to the 10-200 RPMs interval shown in blue in **B**.

Figure S6: Rif1 deletion increases the number of chromatin contacts. Related to Fig. 5. Heat maps showing the $\log_{10}$(reads+1) of the positions (blue lines) identified as significantly different between two $Rif1^{+/+}$ and two $Rif1^{-/-}$ ESC lines from the FourCSeq software (FDR < 0.1 and p-value < 0.001, cf. supplemental materials). The 2Mb region around the viewpoint is indicated in pink.

Figure S7: Failure to re-express Rif1 in G1 leads to nuclear architectural changes prior to S-phase. Related to Fig. 6. A Average genome-wide distribution of replication timing, RT score=$\log_2$(early/late), from Nimblegen tiling arrays with average tile size of 50bp. Shown is the distribution for $Rif1^{+/+}$ and $Rif1^{-/-}$ in pMEFs during the first S-phase after Rif1 deletion. **B** Scheme of the experimental setting employed. OHT=4-hydroxytamoxifen. **C** Ratio (fold increase) of the average number of fragments containing the indicated RPM's ranges in two individually-considered $Rif1^{-/-}$ versus two $Rif1^{+/+}$ (dashed line) pMEFs calculated over all the viewpoints. The increase of fragments containing 10-200 RPMs in $Rif1^{-/-}$ is statistically significant as calculated by two ways ANOVA test based on conditions and viewpoints (**P=0.009). The error bars indicate

standard deviations. In this figure all fragments which belong to either class (10-200 or above 200 RPMs) are used for the calculation, while Fig. 6C is based on those interactions consistently identified in all replicates within a condition as calculated by the r3Cseq software package. **D** Boxplot showing the distribution of replication-timing values of the interactions within the TADs shared between Rif1$^{+/+}$ and Rif1$^{-/-}$ for the indicated viewpoints. The black boxplots show the distribution of the replication timing of the shared chromatin interactions plotted against the Rif1$^{+/+}$ replication timing. As expected, these interactions fall in the same replication timing as the viewpoint. The gray boxplots show the distribution of the replication timing of the shared chromatin interactions plotted against the Rif1$^{-/-}$ replication timing. The shift of the median indicates that the switch of replication timing of the viewpoint in the first S-phase after Rif1 deletion is concomitant with replication timing shifts of only a portion of the interacting TADs. **E** Exemplary plots of replication-timing changes (RT=$\log_2$(early/late)) between one Rif1$^{+/+}$ and one Rif1$^{-/-}$ pMEFs during the first cell cycle (Cornacchia et al., 2012) along with corresponding replication-timing profiles in other published cell types (http://www.replicationdomain.com/). **F** Relative distribution of the fragments containing the indicated RPMs obtained by combining all pMEFs Rif1$^{+/+}$ (black line) and Rif1$^{-/-}$ (grey line) reads, over all the viewpoints, normalizing the library size to 1 million. The bimodal distribution allows the identification of 10 RPMs as cutoff to exclude interactions falling within the noise range (yellow shadowing). The blue shadowing corresponds to the 10-200 RPMs interval shown in blue in **C**.

Table S1

	Early	Late	Undefined RT	TOTAL
**TSS/no Rif1 peak**	15,131	7,297	1,777	24,205
**TSS/overlapping Rif1 peak**	3,171	2,522	328	6,021
**TSS**	18,302	9,819	2,105	30,226

Table S2

Primer	Sequence 5' – 3'	Location (mm9)	Description
P1 fwd	ACTTAGGGCAGCCCAATTCC	chr5:45840960-45841085	Late 1
P1 rev	TAGGTGTTCCCTAGGCCTCC		
P2 fwd	AGAACTGGAGGAACGCCTTG	chr3:93247689-93247796	Late 2
P2 rev	TCCTGCCTCTCTTGCCTTTG		
P3 fwd	TGCTCTCGCTTGGTGACATT	chr6:21164727-21164854	Late 3
P3 rev	AGGTTAGGCAAAGGCGTCTC		
D1 fwr	TCCAAAGGGCTTGCATCACT	chr5:31465642-31465751	Early 1
D1 rev	ATGCAGTGCTATGGGGTCAC		
D2 fwr	GACCACGTTTCTTGCCGTTC		Early 2
D2 rev	GCAGTGATGTCCACAAGGGA	chr2:119252415-119252507	

Major satellite Primers	Sequence 5' – 3'
MajSatFwd	GACGACTTGAAAAATGACGAAATC
MajSatRev	CATATTCCAGGTCCTTCAGTGTGC

Table S3

Illumina adaptors:

P5: 5'-AATGATACGGCGACCACCGAGATCTACACTCTTTCCCTACACGACGCTCTTCCGATCT-3'

P7: 5'-CAAGCAGAAGACGGCATACGAGATCGGTCTCGGCATTCCTGCTGAACCGCTCTTCCGATCT-3'

**ESC viewpoints**	**HindIII**	**DpnII**
E1	**P5-CCGTAT** AGCTTCTTGCTGTCAAGCTT	**P7-**TGGAGTGCTGATGAAAGTCA
E2	**P5-TGAGTG** GTGTTCTTGGTTGGAAGCTT	**P7-**GCCCAGCACCATAGAAACTG
L1	**P5-CATTCA** TCCCTCGCTCTCACAAGCTT	**P7-**CCCTCATAGAAGGTGCCATT
L2	**P5-ATTATA** AAACCCAACTTGCCAAGCTT	**P7-**CACGATGTGCTTCCTGTCAT
LL	**P5-GGCCAC** TGGATAGTGGTCTAAAGCTT	**P7-**GGCTCCAGTGACATCCATG

**pMEF viewpoints**	**HindIII**	**DpnII**
E1	**P5-GGCCAC** GCCCAGCTTGTTCGAAGCTT	**P7**-CGTAAGGGTTGGTGAGATGG
L1	**P5-CATTCA** TGTGGCATGTATGGAAGCTT	**P7**-CTCAGTGGAGCCAAGAAACC
L2	**P5-GAGAGT** CCAGAAGGTTCTTACAAAGCTT	**P7**-CTCAGTGGAGCCAAGAAACC
LL	**P5-CCGTAT** AGCTTCTTGCTGTCAAGCTT	**P7**-TGGAGTGCTGATGAAAGTCA
EE	**P5-TGAGTG** CAGTTAGCAAGGACCAAGCTT	**P7**-CCTGGTGGCAGAGAACTTTA

Table S4

Primer	Sequence 5'- 3'	Location interaction (mm9)	Description
Int. 1 fwd	TGTTTATAAGCCCTCAGGTACATA	chr17:21762323 -21772199	Viewpoint E1
Int. 1 rev	GACAAATCACAACAGGAGAAGTAATAG		
Int. 2 fwd	ATTCCTATGTTTATAAGCCCTCAG	chr17:21714258 -21717665	Viewpoint E1
Int. 2 rev	AGCGGTCCTTGCCAATTATG		
Int. 3 fwd	ATTCCTATGTTTATAAGCCCTCAG	chr17:21096804 -21100021	Viewpoint E1
Int. 3 rev	AGCTAGAGCTATTTCAGTGGAAAC		
Int. 4 fwd	TCAGGAGACAACTTCGTGGAG	chr16:77299025 -77301113	Viewpoint L2
Int. 4 rev	TGAAAAGATGAAGAGGCAAAGG		
Rnr2-1*	TAGGGATAACAGCGCAATCC		Gen. DNA quant.
Rnr2-2*	GACTTTAATCGTTGAACAAACGAAC		
XBP1-1**	GCCCTCCCTGAAAATAAGGA		Ligation control
XBP1-2**	GACTTCTCACCTGGGCCTACA		
HindIII-neg fwd	CCCATTCCTGAGGGTTAGCA		Gen. DNA quant.
HindIII-neg rev	GAAGGAGTGAGAACCGTGCA		
HindIII fwd	GTCATGCTGCCACAGTCTCT		Digestion efficiency
HindIII rev	CCTTCAAGGTTCAGGGGACT		

Taqman probes	Sequence
E1	5'FAM-AAGCTTGACAGCAAGAAGCTCTGGTTT-3'TAMRA
L2	5'FAM-AAGCTTGGCAAGTTGGGTTTAATCTCC-3'TAMRA
RNR2*	5'FAM-CGACCTCGATGTTGGATCAGGACATCC-3'TAMRA
XBP**	5'FAM-AAAGCTTGCACCCTGCTTTAGTGGCC-3'TAMRA

BAC Viewpoint	BAC Interaction
E1: RP23-355H20	1: RP23-234E12
E1: RP23-355H20	2: RP23-234E12
E1: RP23-355H20	3: RP23-209P21
L2: RP23-323D13	4: RP23-325L9

*: Medvedovic, J. *et al*. Flexible long-range loops in the VH gene region of the Igh locus facilitate the generation of a diverse antibody repertoire. *Immunity* **39**, 229-244 (2013).

: Splinter, E. *et al*. The inactive X chromosome adopts a unique three-dimensional conformation that is dependent on Xist RNA. *Genes & development* **25, 1371-1383 (2011).

Supplemental Table Legends

Table S1: Related to Fig. S3. Contingency table for occurrences of 30,226 TSS in replication-timing domains, with/without overlapping Rif1 signal in one representative out of three lines analyzed. We observe 25% (2,522/9,819) of TSS embedded in late replicating domains and 17.3%. (3,171/18,302) in early being covered by a Rif1 signal. Therefore the association of Rif1 to late TSSs shows a slight but significant enrichment ($P<10^{-34}$) compared to the genome-wide expectation of 20%. Similarly, the fraction of TSS with a peak is higher in late domains (41.9%=2,522/6,021) compared to the expectation (32.4%=9,819/3,0226).

Table S2: Related to Fig. S2. List of the primers used for the ChIP validation by qPCR. Primers were designed on region with different Rif1 intensities and non-repetitive. The primers for the qPCR validation of the modified histones ChIPs are from (Lehnertz et al., 2003).

Table S3: Related to Experimental Procedures. List of the primers used for 4C-Seq library preparation. Sequences of the standard Illumina primers are reported (P5 and P7) and are the same for each of the viewpoints specific primers. The tables summarize the sequences of the viewpoint specific part of the primers. In bold is the barcode employed for multiplexing during sequencing. HindIII=forward primers; DpnII=reverse primers.

Table S4: Related to Experimental Procedures and Fig. S5. List of the primers and Taqman probes used for the 3C-qPCR. Primers were designed on the selected interactions indicated. List of BACs used to build control libraries for testing the primers.

Supplemental Experimental Procedures

Mouse lines

$Rif1^F$ and $Rif1^{FH}$ mouse lines were described respectively in (Buonomo et al., 2009; Cornacchia et al., 2012). The $Rif1^F$ mice were crossed with the strain B6;129 gt(ROSA)26Sor$^{tm1(cre/Esr1)Nat}$/J (Jackson laboratory) to introduce the inducible Cre.

Derivation of embryonic stem cell lines

Derivation of ESCs was carried out according to the protocol described by (Bryja et al., 2006) with the following changes. Blastocyst outgrowth was cultured on passage 1 MMC-treated pMEFs (feeders) plated at a density of about 350,000 cells/cm^2. During the first passages disaggregation was performed with 0.25% trypsin. Once established, ESCs colonies were expanded using 0.05% trypsin, on feeders plated at a density of about 175,000 cells/cm^2 in ES medium=Knockout-DMEM (Gibco 10829-018), containing 15% heat-inactivated fetal bovine serum, 1% Penicillin/Streptomycin (Gibco 15070063), 1% L-Glutamine (Gibco 25030-081), 1% non-essential amino acids (Gibco 11140-050), 0.1mM 2-Mercaptoethanol (Gibco 31350-010) and supplemented with 20 ng/ml leukemia inhibitory factor (LIF, EMBL Protein Expression and Purification core facility). Gelatin adaptation was performed by thawing ESCs frozen with feeders directly onto gelatinized plate (0.1% bovine gelatin Sigma G9391 in PBS) in ES medium, supplemented with 1µM MEK inhibitor PD0325901 and 3µM GSK3 inhibitor CH99021 (The University of Dundee, Dept. of Biochemistry, Medical Sciences Institute). ESCs were then cultured until stable growth was achieved. Cells were cultured at 37°C in 7.5% CO_2.

Cell manipulations

Derivation of pMEFs and synchronization experiments were carried out according to (Cornacchia et al., 2012). Immortalization by Large T antigen, infection with retroviruses carrying empty vector or Cre and growth curves were all performed as described in (Buonomo et al., 2009). Time courses of Rif1 deletion by Cre induction in ESCs were done as follows. Gelatin-adapted ESCs were freshly thawed and cultured for two passages prior to deletion. At the third splitting, 200nM 4-hydroxytamoxifen (OHT, Sigma H7904) was added to the medium. Medium was then replaced every two days with freshly added OHT. ESCs proliferation was evaluated by dividing the number of cells collected from duplicate wells for each of the six clones two and four days after plating by the number of cells plated at day 0. The experiment was repeated three times. LT-MEFs growth was evaluated by dividing the number of cells collected in triplicate wells at four and six days after plating by the number of cells plated at day 0. Two Rif1$^{+/+}$ and three Rif1$^{F/F}$ LT-MEF lines infected by a Cre-encoding retrovirus were used. The MTT assay (Roche 1465007) was performed according to the manufacturer instructions, plating 500 cells/well of a 96 well plate in quadruplicate for all six clones. The quantitative alkaline phosphatase assay (Millipore SCR066) was performed according to the manufacturer instructions, plating 20,000 cells/reaction in triplicate for two clones. The experiment was performed once.

Replication timing

The analysis was performed as described in (Ryba et al., 2011). Briefly, asynchronously cycling cells were pulse labeled with the nucleotide analog 5-bromo-2-deoxyuridine (BrdU). The cells were sorted into early and late S-phase fractions on the basis of DNA content using flow cytometry. BrdU-labeled DNA from each fraction was

immunoprecipitated with an anti-BrdU antibody, amplified, differentially labeled and co-hybridized to a whole-genome comparative genomic hybridization microarray. The data from each channel was normalized using Limma package (Smyth, 2004). The $\log_2$(early/late) value was smoothed and plotted against the position along the chromosome to generate RT profiles. Comparison of replication-timing profiles derived from different cell types allows the identification of replication-timing domains defined as the unit of regulation of replication timing (400-800 kb) (Takebayashi et al., 2012). In this comparison replication-timing domains will coincide with the unit of replication-timing changes between cell types. This also highlights that when the changes in replication timing resulting from Rif1 deletion encompass more than a megabase, likely multiple adjacent smaller domains with similar replication timing are being affected. Percentage of replication timing changes in Fig. 1B is calculated as the number of probes on the array that change by a factor more then 1 versus the total number of probes. The genome-wide distributions of replication timing in Fig. 1C and Fig. S7A were calculated directly from the RT-scores on each tile of the array. For ES cells the tiles have a size of 60bp, for pMEF cells the average size is 50bp. Replication timing data are available in the ArrayExpress database (www.ebi.ac.uk/arrayexpress) under accession number E-MTAB-3506.

Transcriptome analysis

Total RNA was isolated using the TRIzol reagent (Invitrogen #15596018) following the manufacturer's instructions and treated with DNase (Promega RQ1 RNase-Free DNase: M6101). RNA quality was assessed on the Agilent 2100 Bioanalyzer (Agilent Technologies) using the RNA6000 Nanokit (#50671511 Nano), and RNA quantity was

measured with ND-1000 NanoDrop spectrophotometer. 1μg of RNA sample was used for microarray analysis on Affymetrix Mouse Gene 1.0ST array (Affymetrix). Robust multi-array average (RMA) normalization was applied. Normalized data were then filtered based on the Affymetrix detection call so that only probes that had a Present call in at least one of the arrays were retained. The transcriptome analysis of LT-MEF was then performed as in (Cornacchia et al., 2012). In brief, cells were infected four times every 12 hours with either a Cre encoding retrovirus or empty vector, then selected for four days with hygromycin 90 μg/mL and collected for analysis. Deregulated genes whose expression is changed as a consequence of Cre expression rather then Rif1 deletion have been excluded by parallel microarray analysis of RNA from three $Rif1^{+/+}$ lines infected with either Cre-encoding (Cre) or empty vectors (EV). CEL files were imported in GeneSpringGX 11.5 software and expression values were filtered by percentile (20-100). t-test with an asymptotic P value and no correction was applied for statistical analysis. ESCs transcriptome analysis was performed in R using Limma package (Smyth, 2004). In both cases, a fold change cutoff of 2 was applied to detect the significantly differentially expressed genes. Microarray data are available in the ArrayExpress database (www.ebi.ac.uk/arrayexpress) under accession number E-MTAB-3503 for the ES cells and accession number E-MTAB-3501 for LT-MEF.

ChIP

Chromatin immunoprecipitation was performed accordingly to (Bulut-Karslioglu et al., 2012) with the following modifications. For Rif1 ChIP, $Rif1^{FH/FH}$ and $Rif1^{+/+}$ ESCs were harvested from four 15cm dishes at 80% confluency. Cells were first cross-linked using 2mM disuccinimidyl glutarate (DSG Synchem UG & Co. KG # BC366) in PBS for 45

min. at room temperature while rotating, washed twice in PBS, followed by 10 min. of additional crosslinking in 1% formaldehyde. After 5 min. quenching in 0.125M glycine at room temperature, chromatin fragmentation was performed using either a Covaris S2 or Vibrocell VCX 400 to produce a distribution of fragments enriched between 200 and 600bp. Chromatin was quantified by ND-1000 NanoDrop spectrophotometer. Immunoprecipitation was performed with anti-HA antibody (Roche monoclonal 3F10 # 11867423001) using 0.5μg of anti-HA antibody and 50μl of Dynabeads protein G (Invitrogen 100.03D) per chromatin corresponding to 25μg of DNA. The reason the anti-FLAG antibody was not used in parallel is because the double crosslinking process renders the epitope unrecognizable for the antibody.

For modified histone's ChIP-seq 2 $Rif1^{-/-}$ and 2 $Rif1^{+/+}$ ESC lines were harvested from one 15cm dishes 2 days after Rif1 deletion was induced. Cells were only cross-linked in 1% formaldehyde. Chromatin fragmentation was performed using either a Covaris S220 to produce a distribution of fragments enriched between 200 and 500bp. Chromatin was quantified by Qbit (Life Technologies Q32851). Immunoprecipitation was performed by incubating 5μg of chromatin over night in the cold with either anti-H3K4me3 (0.2μg), or anti-H3K9me3 (2.4μg), or anti-H3K27me3 (6μg), or anti-H4K20me3 (0.4μg) antibodies or IgG and 30μl of Dynabeads protein G (Invitrogen 100.03D) per ChIP. Retrieved DNA was quantified by Qbit (Life Technologies Q32851). From the control immunoprecipitation ($Rif1^{+/+}$ ESCs for Rif1 and IgG for histone ChIPs) was not possible to retrieve enough DNA to produce a library. The primers used for qPCR validation of the ChIP data are indicated in the Table S2 (Lehnertz et al., 2003).

ChIP sequencing and analysis

ChIP-seq libraries were prepared with NEBNext ChIP-Seq Library Prep Master Mix Set for Illumina (NEB#E6240S/L), except that bead purification was employed (Agencourt AMPure XP beads, Beckman Coulter A63881). Size selection was performed on 2% agarose gels (E-Gel Invitrogen G6610-02). For Rif1 ChIPs, generally 13 PCR cycles for 10ng starting material were applied. We titrated the number of cycles in order to obtain the best quality libraries and set on 13 because is the number that gave the least amount of PCR artifacts (duplication rate). Libraries were barcoded with the NEBNext Multiplex Oligos for Illumina (Index Primers Set 1, NEB#E7335S/L).

The paired-end reads of all ChIP-seq samples were mapped with bowtie2 ((Langmead and Salzberg, 2012) version 2.0.5) against the mouse genome mm8 with a maximum fragment length of 1000bp. MACS (https://pypi.python.org/pypi/MACS2) (with the --broad option) was used to define specifically enriched regions for Rif1. To correlate the Rif1 peaks with other genomic annotations, we determined the number of enriched regions overlapping with replication domains, cLADs and ciLADs (Meuleman et al., 2013), exons and intergenic regions obtained from the UCSC table browser. The expected overlap and the variance for a given annotation were estimated from 50 random sets of shuffled regions with the same number of features and the same extent. A standard Z-score was calculated; Z=(expected-observed)/(standard deviations). Since the peak calling alone does not fully reflect regions of broad Rif1-enrichment, we also utilized the EDD tool (Lund et al., 2014) with default parameters to detect larger domains. Independently of peak and domain calling, we calculated genome-wide profiles of Rif1 enrichment, $\log_2$(ChIP/Input), for windows of 50bp and normalized with respect to

different sequencing depths. These profiles provide the basis for visualization and downstream analysis with deepTools (Ramirez et al., 2014) to determine the behavior of Rif1-enrichement around selected genomic features, such as the origins of replications.

Rif1 ChIP-seq data are available in the ArrayExpress database (www.ebi.ac.uk/arrayexpress) under accession number E-MTAB-3502. Modified histone's ChIP-seq data are available under accession number E-MTAB-3743.

Models for Origin of Replications

The small nascent strands (SNSs) were obtained from the deori database ((Gao et al., 2012) version 2.1), which includes all 2405 SNSs on chromosome 11 from the study by Mechali et al. (56613063-117015900; mm8) (Cayrou et al., 2011). Based on the domain prediction from replication timing data, the SNSs were classified into 1873 early-replicating SNSs (RT>0.5) and 532 late-replicating SNSs (RT<-0.5). 1000 random regions were chosen as control. To supplement the analysis of origins with a motif-based analysis, we obtained the annotation of two complementary models (G4s and OGREs) from the Mechali's group (personal communication, (Cayrou et al., 2012)). We identified all the motif instances and examined their occurrence in flanking region of +/- 4kb around TSSs and SNSs. DeepTools (Ramirez et al., 2014) were used to visualize and summarize Rif1 data and other features around TSSs and SNSs. We generally chose flanking regions of +/-5 kb, except for replication-timing data that are defined for much larger domain. We therefore chose in this case much larger flanking region of +/- 500kb. In most cases we use predefined groups of feature depending on the replication-timing

status, but in Fig 5F and G we used unsupervised clustering (kmeans with k=2) to distinguish CpG-high and CpG-low regions.

LAD

The Lamin B1 associated domains (LADs) for mouse ESC were obtained from the supplementary material of (Peric-Hupkes et al., 2010). For randomized analysis we also generated a shuffled version of these locations with an identical size distribution and excluding original instances of LADs. Using DeepTools ((Ramirez et al., 2014)) we generated genome-wide profiles and heatmaps as for other genomic features (SNSs, G4), but here we added a flanking region of +/- 0.2 Mb. The unsupervised clustering of the heatmaps was done based on the Rif1-data and the replication time was shown in the same order.

Rif1 ChIP and RT change analysis

Replication timing and Rif1 ChIP data were averaged into 200kb windows and LAD score were calculated for the windows by using the predict function in R. Windows that have RT above 0.5 were defined as Early (E) and below -0.5 were defined as Late (L). Rif1 ChIP enrichment was plotted against replication timing differences ($\Delta RT = Rif1^{-/-} - Rif1^{+/+}$) for windows that either remain Early/Late in both $Rif1^{+/+}$ and $Rif1^{-/-}$ ESCs (EtoE and LtoL) or for regions that switch replication timing upon conditional deletion of Rif1 (EtoL and LtoE; $\Delta RT > +1$ and $\Delta RT < -1$). Lamin B1 association distribution for EtoE, LtoL, EtoL and LtoE were represented as boxplots.

4C-Seq

Chromosome conformation capture was performed as described in (Splinter et al., 2012; Stadhouders et al., 2013) with the following changes. 1 or 2 x10^7 cells (ESC and pMEF respectively) were collected for each experiment and cross-linked in 2% formaldehyde. Cell were lysed for 20 min. at 4°C, followed by addition of 0.3% SDS. After 10 min. at 65°C, the lysates were incubated for 1 hour at 37°C. For the first restriction digest, 1000U of HindIII in total were added for two overnight, distributed as follows: 200U for 4 hours, 200U for the first overnight, 200U for 4 hours, 200U for the second, 200U for the last 4 hours. Before the ligation step, HindIII was heat-inactivated. Following steps have been performed as described in (Splinter et al., 2012). DpnII restriction enzyme was chosen as four-cutter. Synchronized MEFs DNA was digested for two over nights by DpnII. PCR reaction was performed as described in (Splinter et al., 2012), except that Takara Ex Taq DNA Polymerase Hot-start version (Takara RR006B) and 600ng of total template in three reactions of 50 µl each were used. The primers employed to build the library are indicated in the Table S3. Six samples per lane were pooled according to molarity and loaded on a HiSeq 2000 in 100bp single-end mode. Analysis of the 4C-Seq has been performed as described in (Thongjuea et al., 2013). In brief, sequence reads were mapped to the genome. Unaligned reads were tested for the restriction enzyme cutting site and if applicable trimmed and mapped again. Uniquely mapped reads were used for downstream analysis. The number of reads per restriction fragments was counted by applying the method “WholeReads” (Thongjuea et al., 2013). Regions with less then 50 reads were removed from the analysis first. The number of remaining reads was then normalized “PowerLaw fitting curve” (Thongjuea et al., 2013). The normalized number of reads is indicated as RPMs and it is a measure of how many times each interaction is

detected in the library (frequency). Each restriction fragment with at least one RPM and shared by both biological replicates was define as an interaction ("intersection method" (Thongjuea et al., 2013)). After the intersection, we have additionally eliminated from our following analysis any interaction represented by less then 10 RPMs. This cutoff was established based on the distribution of the RPMs in each individual replica and for each of the viewpoints. In Fig. S5H (ESCs) and Fig. S7F (pMEFs) the cumulative curves of RPMs distribution obtained by averaging the biological duplicates across all the viewpoints are shown as representative summary of the behavior of each individual sample. By employing this method we define the significance of the interactions by their presence in all the biological replicas, followed by their frequency above a background established on the individual samples before intersection, to increase its stringency. This approach, rather then applying a statistical threshold, allows us to take into account also lower frequency interactions. The total number of interactions is calculated as the sum of all restriction fragments showing at least 10 RPMs. The number of interactions inside the replication domain corresponding to each of the viewpoint has been calculated as the sum of restriction fragments showing at least 10 RPMs within the coordinates of the border of the replication-timing domain. A replication-timing domain is defined as a continuous chromosomal region showing the same replication timing. For most of the viewpoints this corresponds also to the replication-timing domains as defined by alignment (ENCODE database on http://genome.ucsc.edu) with exception of viewpoint L1 and E1 for ESCs and L2 in MEFs. In these regions the replication-timing alignment data are not sufficiently clear to define the presence of eventual subdomains. In these cases we therefore considered a single domain the whole region displaying the same replication

timing within 1Mb. 4C-seq data for both ES cells and LT-MEF are available in the ArrayExpress database (www.ebi.ac.uk/arrayexpress) under accession numbers E-MTAB-3500 and E-MTAB-3505 respectively.

4C-Seq and RT changes analysis

The list of topological associating domains (TADs) for mouse ESC was obtained from (Dixon et al., 2012). The interactions *in cis*, outside of the replication domain and with at least 10 RPMs were intersected with TADs. On this basis, interactions present in TADs that are common between Rif1$^{+/+}$ and Rif1$^{-/-}$ were classified as shared, irrespective of the specific fragend mapped. Each TAD contains indeed multiple fragends. TADs containing exclusively Rif1$^{-/-}$-specific interactions were classified as Rif1-null specific. RT score was then assigned to the interactions grouped as above as described in (Ryba et al., 2011).

3C-qPCR

The 3C library was prepared as for the 4C, but without DpnI digest and following ligation. The quality controls and the qPCR assays were performed according to (Hagege et al., 2007). Primers were designed over four different interactions mapped in two independent viewpoints in ESCs. The list of the primers and Taqman probes employed is in the Table S4. The BACs used to build control libraries are also listed in Table S4.

FourCSeq analysis

The 4C-Seq data were also analyzed according to the FourCSeq Bioconductor software package (see http://bioinformatics.oxfordjournals.org/content/31/19/3085). The FourCSeq software uses calibrated and variance-stabilized read count data as input for

the statistical test to identify fragments on the in cis chromosome which differ between the conditions. Sequence reads were mapped to the genome. Unaligned reads were tested for the restriction enzyme cutting site and if applicable trimmed and mapped again. Uniquely mapped reads were used for downstream analysis. In the filtering step fragments with an average read count of at less than 5 RPMs across all samples and fragments within 10000 nucleotides of the viewpoint were removed. To fit the decay trend the asymmetric monotone fit option was chosen. To determine statistically different fragments between the two conditions a threshold of 0.001 for the P-value and of 0.1 for the FDR were applied.

Immunofluorescence and image analysis

Rif1$^{FH/FH}$ ESCs were grown on gelatinized coverslips over-night. Cells were fixed in 4% paraformaldehyde in PBS for 10 min. at room temperature and stored at 4°C in PBS and Sodium Azide. For staining, coverslips were permeabilized in 0.5% Triton X-100 at room temperature for 10 min., and then processed as described in (Buonomo et al., 2009), except that the primary antibodies were incubated 90 min. Images were acquired using a Leica confocal TCS SP5 microscope with a X63 1.4 NA oil objective and run by LAS AF Software (Leica). Contrast adjustment and cropping were performed in Image J. Figures were composed in Illustrator (Adobe). The image analysis of Rif1 enrichment at the nuclear periphery was performed using the free open-source software CellProfiler (Carpenter et al., 2006). In brief, we segmented the whole-nuclear area by a manual threshold of the DAPI image and the nuclear-periphery region by a manual threshold of a top-hat filtered lamin B1 image. We then defined the nuclear-interior by removing the nuclear-periphery mask from the whole-nucleus mask. We measured the mean Rif1 and

DNA intensities in the nuclear periphery and the nuclear interior and computed the ratio. In order to control for the quality of the input data, we only took into account images were at least 95% of all the pixels in the nuclei region showed no saturation.

Protein extracts and Immunoprecipitation

For Fig. 1A, S1C and G proteins were extracted and analyzed as described in (Buonomo et al., 2009).

For Fig. S2D nuclei from Rif1$^{FH/FH}$ and Rif1$^{+/+}$ ESCs were resuspended in cold Benzonase buffer (50mM Tris-HCl pH 8.0, 100mM NaCl, 1.5mM MgCl2, 10% glycerol, freshly added protease and phosphatase inhibitors), subjected to snap freezing, followed by Benzonase digestion (50U/ml, Sigma, E1014). The nuclear solubilized fraction was collected by centrifugation and supernatants were used for immunoprecipitation as follows. 10ml Rif1$^{FH/FH}$ and Rif1$^{+/+}$ extracts were incubated with 8μl/ml anti-HA and anti Flag antibodies overnight at 4°C. 1% Input was collected prior antibody addition. Extracts were incubated with 100μl/ml Protein G Dynabeads (Invitrogen 100-03D) for 2 hours, subsequently 1% flowthrough (FT) was collected. Beads were washed three times in IP-Buffer (50mM Tris-HCl pH 8.0, 150mM NaCl, 1.5mM MgCl2, 10% glycerol, 0.1% Triton X-100 freshly added protease and phosphatase inhibitors) and finally immunoprecipitated proteins (IP) were eluted by boiling beads 10 min. in 2x Laemmli buffer. Input, IP and FT were run on 5% SDS-PAGE.

Antibodies

The anti-mouse Rif1 antibody #1240 has been described in (Buonomo et al., 2009); anti-Smc1 (Bethyl, #A300-055A) 1:10000; anti-phospho Ser345 Chk1 (Cell Signaling

Technology#2348) 1:1000; anti-Chk1 G4 (Santa Cruz #sc-8408) 1:400; anti-Oct3/4 C10 (Santa Cruz #sc5279) 1:200; anti-Nanog (Abcam ab80892) 1:1000; anti-p21 (Santa Cruz #6246) 1:200; anti-HA (Covance monoclonal HA.11 clone 16B12 #MMS-101R) 1:1000 for western blotting, 1:3000 for immunofluorescence; anti-HA ChIP and immunoprecipitation (Roche 11867423001); Anti-Flag ChIP and immunoprecipitation (Biozol 637302); rat IgG (Santa Cruz 2026); anti-lamin B1 (Abcam #ab16048) 1:1000 for both western blotting and immunofluorescence; anti β tubulin (Cell Signaling #2146) 1:1000.

3D FISH and analysis

3D FISH protocol is based on (Solovei et al., 2002) with the following changes. Slides were equilibrated in 20% glycerol in PBS for 1 hour and subsequently frozen in liquid nitrogen and thawed at room temperature. This step was repeated three times. For long-term storage, slides were kept in 70% ethanol at -20°C. Before use, slides where treated with 0.1mg/ml RNase A in 2x SSC for 1 hour at 37°C. BAC probes RP23-355H20 and RP23-209P21 (Source Bioscience) were labeled for 50 min. using Roche Nick translation kits (DIG 11745816910 and Biotin 11745824910), precipitated in a ratio 1:1 and resuspended in 70% formammide (Sigma F9037), 2x SSC and 10% dextran sulfate. Denaturation was performed for 8 min. at 85°C and hybridization was kept overnight. Post-hybridization washes were done with 0.1x SSC pre-warmed at 61°C. Blocking was carried out for 30 min. with 3% BSA, 4x SSC, 0.1% Tween 20. Anti-Dig-FITCH (Roche 11207741-910) and Strepavidin-Alexa546 (Invitrogen S-11225) were incubated for 1 hour at 37°C at a dilution 1:200 in 1% BSA, 4x SSC and 0.1% Tween 20. Acquisition was performed on a Confocal Leica TCS SP5 using a 63X oil immersion objective with

an extra optical magnification of 4x and optical sections separated by 0.25 μm. Cells with signals from both alleles were analyzed by Imaris Bitplane. During the statistical analysis of the measurements of the distance between the two FISH signals, values exceeding 2.5 the standard deviations were excluded.

Supplemental references

Bryja, V., Bonilla, S., and Arenas, E. (2006). Derivation of mouse embryonic stem cells. Nat Protoc *1*, 2082-2087.

Bulut-Karslioglu, A., Perrera, V., Scaranaro, M., de la Rosa-Velazquez, I.A., van de Nobelen, S., Shukeir, N., Popow, J., Gerle, B., Opravil, S., Pagani, M., *et al.* (2012). A transcription factor-based mechanism for mouse heterochromatin formation. Nature structural & molecular biology *19*, 1023-1030.

Buonomo, S.B., Wu, Y., Ferguson, D., and de Lange, T. (2009). Mammalian Rif1 contributes to replication stress survival and homology-directed repair. The Journal of cell biology *187*, 385-398.

Carpenter, A.E., Jones, T.R., Lamprecht, M.R., Clarke, C., Kang, I.H., Friman, O., Guertin, D.A., Chang, J.H., Lindquist, R.A., Moffat, J., *et al.* (2006). CellProfiler: image analysis software for identifying and quantifying cell phenotypes. Genome Biol *7*, R100.

Cayrou, C., Coulombe, P., Puy, A., Rialle, S., Kaplan, N., Segal, E., and Mechali, M. (2012). New insights into replication origin characteristics in metazoans. Cell cycle (Georgetown, Tex *11*, 658-667.

Cayrou, C., Coulombe, P., Vigneron, A., Stanojcic, S., Ganier, O., Peiffer, I., Rivals, E., Puy, A., Laurent-Chabalier, S., Desprat, R., *et al.* (2011). Genome-scale analysis of metazoan replication origins reveals their organization in specific but flexible sites defined by conserved features. Genome Res *21*, 1438-1449.

Cornacchia, D., Dileep, V., Quivy, J.P., Foti, R., Tili, F., Santarella-Mellwig, R., Antony, C., Almouzni, G., Gilbert, D.M., and Buonomo, S.B. (2012). Mouse Rif1 is a key

regulator of the replication-timing programme in mammalian cells. The EMBO journal *31*, 3678-3690.

Dan, J., Liu, Y., Liu, N., Chiourea, M., Okuka, M., Wu, T., Ye, X., Mou, C., Wang, L., Wang, L.*, et al*. (2014). Rif1 Maintains Telomere Length Homeostasis of ESCs by Mediating Heterochromatin Silencing. Dev Cell *29*, 7-19.

Dixon, J.R., Selvaraj, S., Yue, F., Kim, A., Li, Y., Shen, Y., Hu, M., Liu, J.S., and Ren, B. (2012). Topological domains in mammalian genomes identified by analysis of chromatin interactions. Nature *485*, 376-380.

Gao, F., Luo, H., and Zhang, C.T. (2012). DeOri: a database of eukaryotic DNA replication origins. Bioinformatics (Oxford, England) *28*, 1551-1552.

Hagege, H., Klous, P., Braem, C., Splinter, E., Dekker, J., Cathala, G., de Laat, W., and Forne, T. (2007). Quantitative analysis of chromosome conformation capture assays (3C-qPCR). Nat Protoc *2*, 1722-1733.

https://pypi.python.org/pypi/MACS2.

Langmead, B., and Salzberg, S.L. (2012). Fast gapped-read alignment with Bowtie 2. Nat Methods *9*, 357-359.

Lehnertz, B., Ueda, Y., Derijck, A.A., Braunschweig, U., Perez-Burgos, L., Kubicek, S., Chen, T., Li, E., Jenuwein, T., and Peters, A.H. (2003). Suv39h-mediated histone H3 lysine 9 methylation directs DNA methylation to major satellite repeats at pericentric heterochromatin. Curr Biol *13*, 1192-1200.

Lund, E., Oldenburg, A.R., and Collas, P. (2014). Enriched domain detector: a program for detection of wide genomic enrichment domains robust against local variations. Nucleic acids research *42*, e92.

Meuleman, W., Peric-Hupkes, D., Kind, J., Beaudry, J.B., Pagie, L., Kellis, M., Reinders, M., Wessels, L., and van Steensel, B. (2013). Constitutive nuclear lamina-genome interactions are highly conserved and associated with A/T-rich sequence. Genome Res *23*, 270-280.

Peric-Hupkes, D., Meuleman, W., Pagie, L., Bruggeman, S.W., Solovei, I., Brugman, W., Graf, S., Flicek, P., Kerkhoven, R.M., van Lohuizen, M., *et al.* (2010). Molecular maps of the reorganization of genome-nuclear lamina interactions during differentiation. Molecular cell *38*, 603-613.

Ramirez, F., Dundar, F., Diehl, S., Gruning, B.A., and Manke, T. (2014). deepTools: a flexible platform for exploring deep-sequencing data. Nucleic acids research.

Ryba, T., Battaglia, D., Pope, B.D., Hiratani, I., and Gilbert, D.M. (2011). Genome-scale analysis of replication timing: from bench to bioinformatics. Nat Protoc *6*, 870-895.

Smyth, G.K. (2004). Linear models and empirical bayes methods for assessing differential expression in microarray experiments. Stat Appl Genet Mol Biol *3*, Article3.

Solovei, I., Cavallo, A., Schermelleh, L., Jaunin, F., Scasselati, C., Cmarko, D., Cremer, C., Fakan, S., and Cremer, T. (2002). Spatial preservation of nuclear chromatin architecture during three-dimensional fluorescence in situ hybridization (3D-FISH). Experimental cell research *276*, 10-23.

Splinter, E., de Wit, E., van de Werken, H.J., Klous, P., and de Laat, W. (2012). Determining long-range chromatin interactions for selected genomic sites using 4C-seq technology: from fixation to computation. Methods *58*, 221-230.

Stadhouders, R., Kolovos, P., Brouwer, R., Zuin, J., van den Heuvel, A., Kockx, C., Palstra, R.J., Wendt, K.S., Grosveld, F., van Ijcken, W., *et al.* (2013). Multiplexed

chromosome conformation capture sequencing for rapid genome-scale high-resolution detection of long-range chromatin interactions. Nat Protoc *8*, 509-524.

Takebayashi, S., Dileep, V., Ryba, T., Dennis, J.H., and Gilbert, D.M. (2012). Chromatin-interaction compartment switch at developmentally regulated chromosomal domains reveals an unusual principle of chromatin folding. Proceedings of the National Academy of Sciences of the United States of America *109*, 12574-12579.

Thongjuea, S., Stadhouders, R., Grosveld, F.G., Soler, E., and Lenhard, B. (2013). r3Cseq: an R/Bioconductor package for the discovery of long-range genomic interactions from chromosome conformation capture and next-generation sequencing data. Nucleic acids research *41*, e132.
